# Spiropyran as Building Block in Peptide Synthesis and Modulation of Photochromic Properties

**DOI:** 10.1021/acs.orglett.4c03929

**Published:** 2024-12-02

**Authors:** André Paschold, Niclas Starke, Sven Rothemund, Wolfgang H. Binder

**Affiliations:** † Macromolecular Chemistry, Institute of Chemistry, Faculty of Natural Science II, Martin Luther University Halle Wittenberg, von-Danckelmann-Platz 4, 06120 Halle, Germany; ‡ Core Unit Peptide−Technologies, University of Leipzig Medical Center, Liebigstraße 21, 04103 Leipzig, Germany

## Abstract

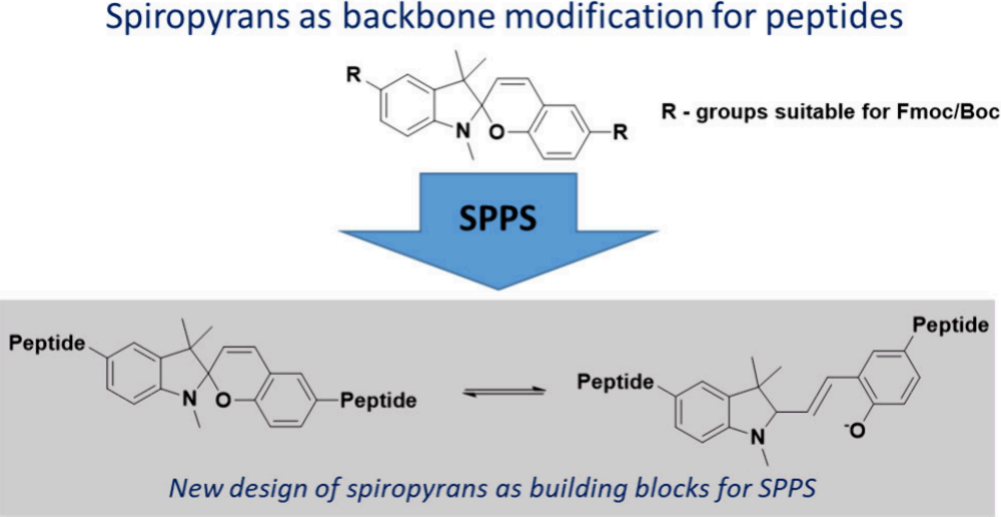

Light-controlled triggering of materials requires efficient embedding of molecular photoswitches into larger molecules. We herein present the synthesis of two new building blocks for the synthesis of photoswitchable peptides, embedding spiropyranes as a central unit into peptide-backbones via a novel, yet unreported approach. The synthesis presented here allows us to embed spiropyranes directly into solid-phase peptide synthesis (SPPS), further describing the resulting photophysical properties of the as-prepared photoswitchable peptides.

The incorporation of photoswitchable moieties into functional materials^1^ allows to address molecular conformation^2^ and dynamics^3^ by light as external trigger.^4^ Besides azobenzenes,^5^ diarylethenes,^6^ and thioindigos,^7^ spiropyrans are important photoswitches.^8^ Spiropyrans exist in two forms (Figure 1A): a colorless, closed form of the cyclic spiropyran (SP) and the colored merocyanin (MC) form, the latter displaying an extended molecular shape. Irradiation by UV-light causes cleavage of the C–O bond at the spiro-carbon and its isomerization to the MC-form, reversing isomerization by visible light.^9^ There is a strong change in the dipole-moment upon isomerization:^10^ while the dipole moment of the SP-form is ∼4–6 D, it is increased in the MC-form to ∼14–18 D. Spiropyranes are not only sensitive to light^11^ but can also also be modulated by temperature,^12^ pH,^13^ the redox potential,^14^ solvent polarity,^15^ ions,^16^ and even by mechanical force.^17^ Thus, they are of great interest in the generation of smart materials, where multiple stimuli can be transferred to the shape, strength, and dynamic properties of a material they are embedded into.^10,18^ Their incorporation into biological and synthetic macromolecules was first reported in 1976 for peptides^19^ and in 1978 for synthetic polymers^20^ but only in the side-chain of the respective monomer unit. Thus, for decades, side-chain or end-group modification of peptides and polymers^21^ (Figure 1B) remained the only method to incorporate spiropyrans into (biological) macromolecules to transfer their adaptive and stimuli-responsive properties therein. In 2013 the spiropyran unit was embedded directly into a polymer-backbone, revealing significant property changes upon their photoswitching.^22^ While spiropyrans play an important role in the control of peptide properties,^8b,23^ a general methodology to embed spiropyrans into the main backbone of a peptide is still missing. We herein report new spiropyran building blocks (Figure 1C) and their incorporation into peptides via SPPS (solid-phase peptide synthesis): two blocks via a Fmoc-synthesis strategy and two blocks via a Boc-strategy. Based on suitable indole and salicylaldehyde precursors, equipped with either a protected amine- or a carboxy functionality, the desired spiropyran can be formed subsequently be embedded into peptides via Fmoc-SPPS at different positions of a fibrillating peptide (see Table 1), and further investigated for their photophysical properties.

**Figure 1:**
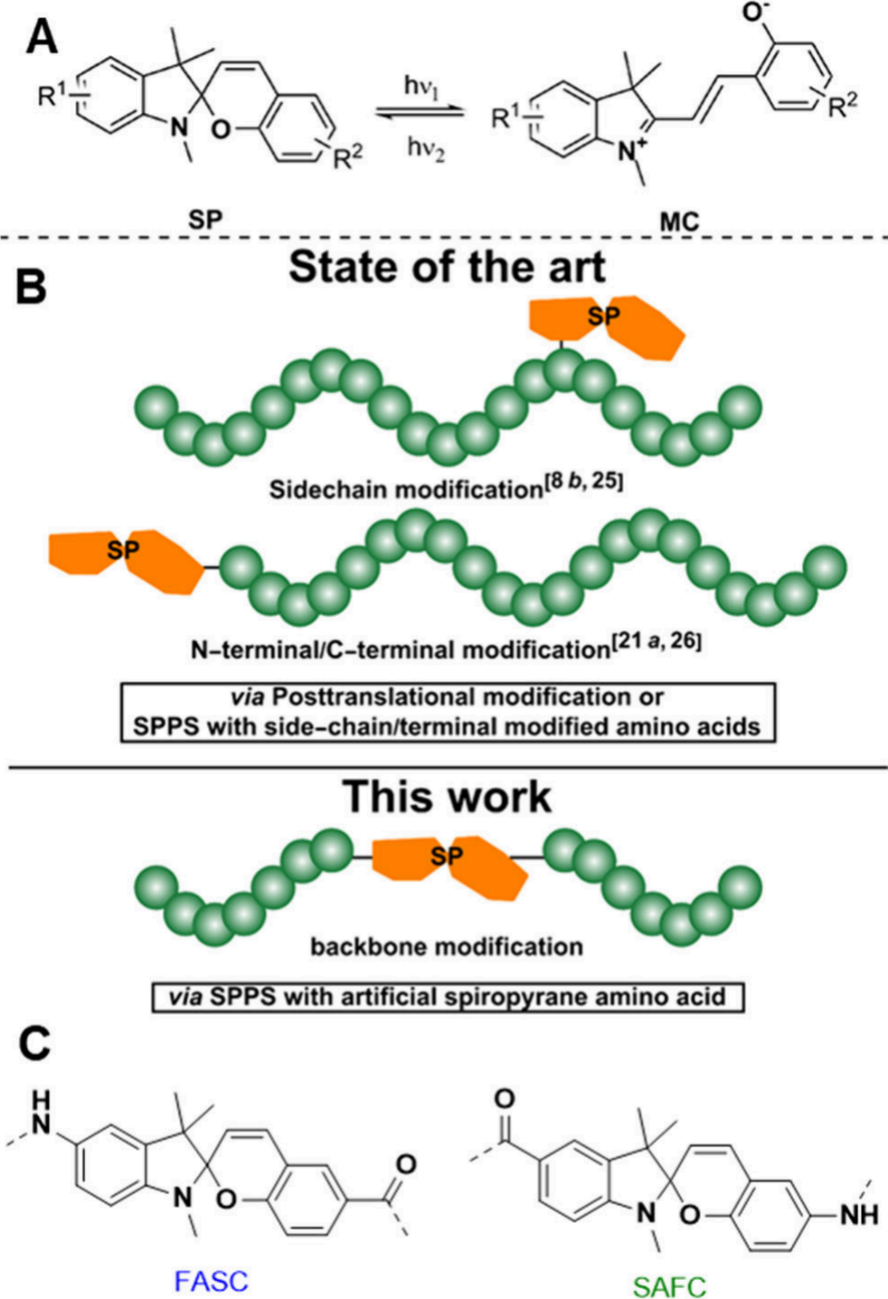
(A) Isomerization of spiropyrans between their closed form (SP) and their ring-opened merocyanine form (MC). (B) State-of-the-art strategies to modify peptides with spiropyrans consists of post-translational modifications of the synthesized peptide at the side chain^8b,25^ and at the N-/C-terminal end of the peptide chain (above).^21a,26^ This work: solid-phase peptide synthesis (SPPS) to incorporate a spiropyran building block into the peptide backbone (below). (C) Structures of the spiropyran two different building blocks, FASC and SAFC.

**Table 1:** Peptides Containing the Photoswitchable Spiropyran Moiety (SAFC, FASC) Synthesized *via* Fmoc Solid-Phase Peptide Synthesis

peptide	primary sequence	embedded spiropyran
**P1**	^25^RKKLQ^30^D-FASC-VHNF^35^VAL	**1a**
**P2**	^25^RK-FASC-KLQ^30^DVHNF^35^VAL	**1a**
**P3**	GSGSGS-FASC-GSGSGS	**1a**
**P4**	^25^RKKLQ^30^D-SAFC-HNF^35^VAL	**2a**

As non-natural amino acid building blocks for SPPS, we synthesized the four spiropyrans **1a**, **1b**, **2a**, and **2b** (Scheme 1). Spiropyrans **1a** and **1b** are designed for introducing the FASC building block containing the N-terminal protected amino group at the indolinium entity and the C-terminal carboxy function at the chromene unit. The synthetic strategy was reversed for spiropyrans **2a** and **2b**, introducing the SAFC building block.

**Scheme 1:**
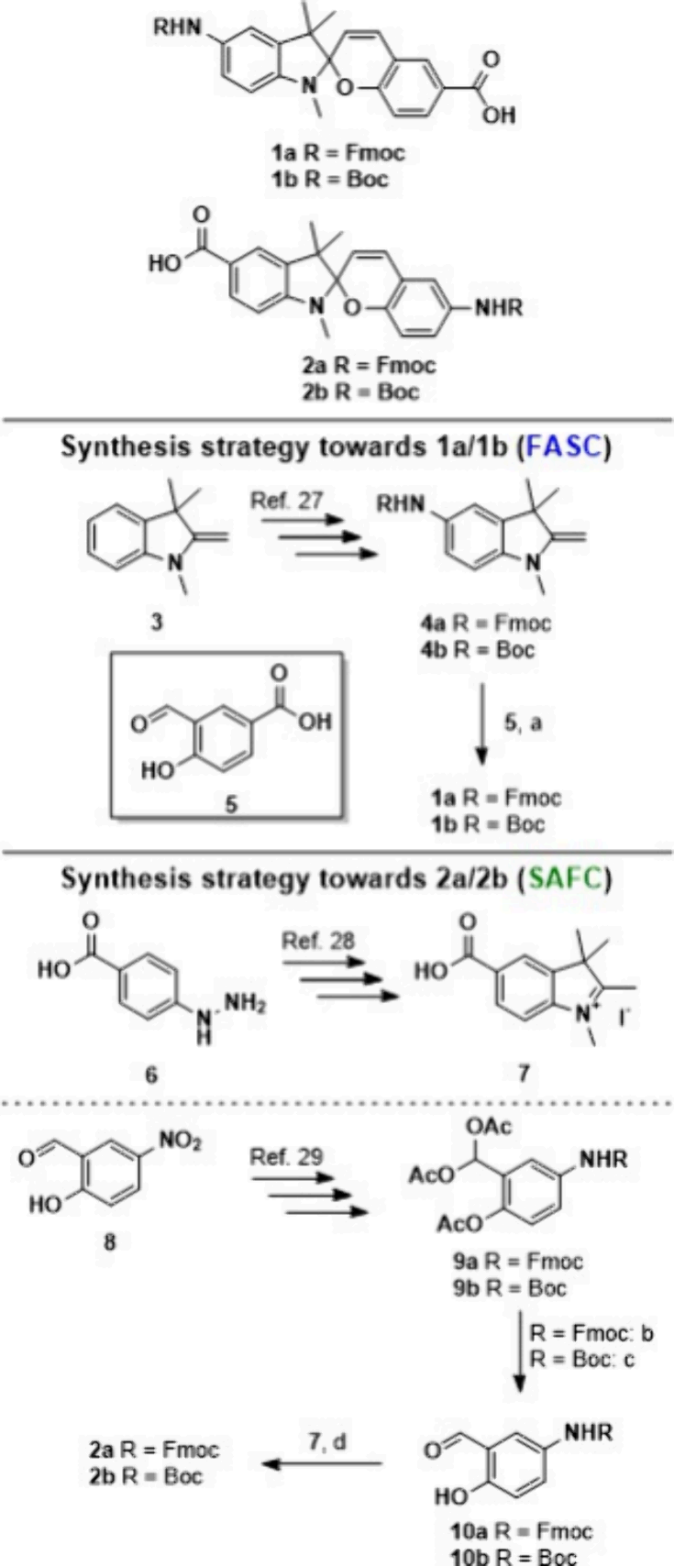
Synthesis of the Spiropyrans **1a**/**1b** and **2a**/**2b**

## Synthesis of the Photoswitch

We designed the building blocks **1a** and **2a** bearing a Fmoc-protection group, and **1b** and **2b** with a Boc-protection group.^24^ The key step in both strategies was a condensation reaction of an indoline compound with an *o*-hydroxy-aromatic aldehyde. The synthesis of the spiropyrans **1a** and **1b** (Scheme 1) started with commercially available **5**, where a nitro group was introduced at position 5.^27^ Subsequently, the nitro group was reduced to the amine^27^ and protected with a Boc- or Fmoc-group. Finally a condensation reaction with commercially available carboxylic acid **5** leads to the desired product **1a** with an overall yield of 5% over 4 steps and the spiropyran **1b** in a yield of 18%. The convergent synthesis toward spiropyrans **2a** and **2b** (Scheme 1) started with the indole precursor. 4-Hydrazinobenzoic acid **6** was converted in a Fischer indole like reaction^28^ followed by a methylation to yield the indolinium iodide **7**.^28^ In contrast to the spiropyrans **1a** and **1b** we additionally prepared *o*-hydroxy benzaldehydes **10a** and **10b**. First step was to protect the hydroxy- and the aldehyde-functionality of nitro salicyl aldehyde **8** with acetoxy groups,^29^ followed by reduction of the nitro group and attaching the group (Boc or Fmoc).^29^ To remove the acetoxy protecting groups, we probed de-esterification under different conditions. The Boc-protected **9a** was treated under basic conditions, while the Fmoc-protected **9b** was treated under acidic conditions. To obtain the final compounds, the indolinium iodide and the respective protected *o*-hydroxybenzaldehyde were converted in a condensation reaction. Spiropyran **2a** was obtained in an overall yield of 4% and spiropyran **2b** in 3%, both in 6 steps.

## Peptide Synthesis and Photophysical Properties

**Scheme 2:**
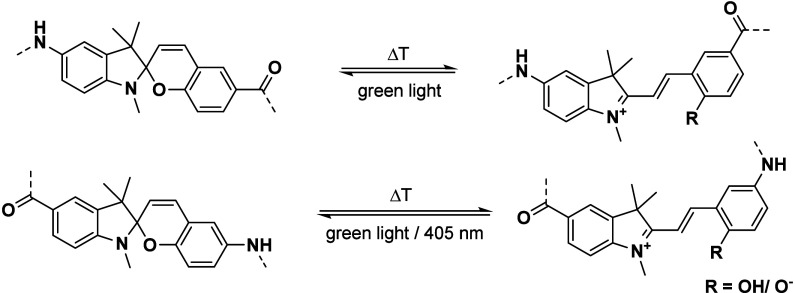
Photophysical Isomerization Process of the FASC (top) and SAFC (bottom) Building Blocks

To test if the Fmoc-protected spiropyrans **1a** and **2a** are compatible with standard SPPS conditions and to investigate the photophysical properties we synthesized four different peptides (Table 1). We chose the fibril core sequence of the parathyroid hormone (PTH_25–37_) as a model peptide^30^ to probe a modulation of the fibrillization of the peptide, in addition to the GlySer-sequence at the N-terminus and the C-terminus. The peptides were synthesized using standard SPPS conditions and characterized with ESI-Tof, MALDI-ToF, and ^1^H NMR (see the Supporting Information). The isomerization between the MC and the SP-form was probed via UV/vis-spectroscopy and HPLC. As spiropyrans display an acidochromic behavior, we investigated the behavior at physiological pH (FASC; buffered aqueous solution, pH 7.4), in a citrate buffered solution (FASC and SAFC; pH 2.5), and in 0.1 M HCl (SAFC; pH 1). For both spiropyran-building blocks (peptides **P1–P4**, Figures S1–S4) the colored MC-form showed absorption in the UV and visible range, while the colorless SP-isomer usually showed absorption only in the UV range. Furthermore, the MC-isomer was the thermodynamically favored form in aqueous solution (Scheme 2, Figures S5–S14), which is well-known for this class of spiropyrans in the presence of water.^31^ Irradiation with green light (525 nm) of **P1**–**P4** led to the SP-isomer, determined by UV/vis-spectroscopy. The thermal (dark) relaxation toward the MC-form follows a first-order kinetic^15b,32^ and occurred fast at 37 °C. At all tested conditions, a half-life time of less than 20 min was observed which increased to up to 1155 min if the temperature was decreased to 4 °C (Table 2, Figures S5–S16). A competitive reaction is the degradation process of the MC-form via hydrolysis of the bridging double bond, which was first described by Stafforst et al.^33^ Decomposition led to a peptide with the Fischer’s base moiety and a peptide bearing a salicyl aldehyde moiety, both detected *via* MALID-ToF measurements (Figure S17–S19). The hydrolysis rate compared to the thermal isomerization of the MC-FASC unit is slower, whereby the rate constant of the hydrolysis is 13 (0.116 min^–1^ vs 0.00921 min^–1^ for **P3**) to 150-times lower (0.237 min^–1^ vs 0.00162 min^–1^ for **P2**, Table S1). In contrast, the SAFC building block could not be handled at a physiological pH value, as the hydrolysis occurs within several minutes.

**Table 2:** Photochromic Properties of Peptides **P1**–**P4** in Aqueous Media

peptide	λ_max, SP_ [nm]	λ_max, MC_ [nm]	λ_iso_ [nm]	ε^a^ [mol^–1^ cm^–1^]	τ_1/2_ at 37 °C (4°) [min]
**P1**^b^	244, 511	244, 376, 518	292	–f	3.74 ± 0.06 (171.43 ± 0.28)
c	250	257, 321, 428	314	6201 ± 386 (314 nm)	10.74 ± 0.02 (1155.2 ± 0.4)
**P2**^b^	242	286, 376, 518	282	–f	2.92 ± 0.04 (321.44 ± 0.30)
c	n.d.	257, 321, 427	314	5910 ± 553 (314 nm)	11.31 ± 0.02 (1151.1 ± 0.2)
**P3**	240	244, 375, 515	283	–f	5.95 ± 0.10 (358.88 ± 0.18)
c	n.d.	257, 323, 426	313	5644 ± 66 (313 nm)	15.78 ± 0.03
**P4**^c^	247, 305	253, 379, 442	265, 326	–f	1.65 ± 0.04
d	305,357,450	253, 379, 442	329	6153 ± 90 (329 nm)	10.04 ± 0.21 (384.88 ± 0.86)

a At the isosbestic point.

b 50 μM NaH_2_PO_4_, 0,01% NaN_3_, pH 7.4.

c 100 μM citric acid/sodium citrate, pH 2.5.

d 1 M HCl, pH 1.

f Not determined as MC-form is hydrolyzed. n.d.: not determined.

If FASC is kept in the SP-form through continuous irradiation with green light, it remains stable until the light source is switched off, and the isomerization and degradation kinetics can be followed via UV/vis-spectroscopy (irradiation time of 5 h). Spiropyrans are known to act as photoacids; therefore, we further investigated the behavior at pH values, where the MC-form should mainly exist in its protonated form. As the p*K*_a_ value of the MC-form was determined to be ∼7.2 for several spiropyrans,^34^ strong acidic conditions (pH 2.5 or pH 1) were used. The FASC-containing peptides are stable (τ_1/2_ of hydrolysis >15,000 min) at a pH value of 2.5. The SP-to-MC isomerization reaches thermal equilibrium usually after 2 h, while the SP-PSS (photostationary state) was obtained through irradiation with green light after 10 min. Analysis with HPLC revealed that the thermal equilibrium consists of nearly 100% of the MC-form, while the photoconversion was almost quantitative (Table S2 and Figures S23 and 24). The SAFC-switch was not stable at pH 2.5, but the decomposition was strongly inhibited (the rate constant was ∼1300 fold smaller than the thermal isomerization, Table S1), so that photophysical parameters could be determined (Table 2). Irradiation at 405 nm leads to a conversion of at least ∼75% to the SP-form, whereby the thermal back-isomerization at 37 °C exhibits a half-life time of 1.65 min, completely stable at pH 1. The photoisomerization at pH 1 could be achieved with irradiation of light of two different wavelengths, where 405 nm led to an SP content of 51% at the PSS, while green light generated 62% (Figure S21).

The thermal back-isomerization exhibits a half-life time of 10 min at 37 °C and 385 min at 4 °C, respectively (Figures S17–19).

We observed that the stability of the spiropyrans in our peptides against hydrolysis was strongly pH-dependent. Thus, the peptide **P2** at pH 7.4 and 37 °C is almost six times more stable than the peptide **P1**. Furthermore, the substituents at the spiropyrane rings also have a significant impact, as the FASC building block could at least be investigated at pH 7.4, while the SAFC building block was hydrolyzed already within a few minutes. Increasing the stability of the spiropyrans against hydrolysis at higher pH values by different substituents is currently a subject of intensive research strategies. Thus, substituents can influence the SP-MC ratio in thermal equilibrium,^35^ wherein the SP-form does not undergo hydrolysis but the hydrolytic rate of the MC-form remains almost unchanged. Another approach is to modulate the stability of the bridging double bond against hydrolysis.^36^ Introducing an electron-donating methoxy group in the *para*-position conjugated with the double bond improved stability. Recently a spiropyran was reported bearing a napthalimide moiety, which was stable at pH 7.^37^

## Conclusion

Herein, we for the first time presented an approach to use Boc- and Fmoc-protected spiropyran photoswitches directly as a backbone modification, applicable via solid phase peptide synthesis (SPPS). We have developed an easy synthetic route and demonstrated the incorporation with standard Fmoc-chemistry as well as the photophysical behavior of the generated, fibrillating peptides under acidic conditions. Our future efforts will focus on the design of new photoswitches to increase the stability at higher pH values and to further modulate aggregation of peptides and proteins.

## Data Availability

Some data underlying this study are not publicly available due to patent issues. Most data of this study however are available within this article and its Supporting Information. The raw data that support the findings of this study are stored electronically according to the requirements of the DFG and are available from the corresponding author upon reasonable request by email (please contact the corresponding author, WHB, via Email (Wolfgang-binder@chemie.uni-halle.de).

## Supporting Information Available

General information, experimental procedures, characterization data, and copies of ^1^H, ^13^C NMR spectra; HPLC data; spectroscopic investigations; Supplementary Tables: (S1) Kinetic parameters of thermal isomerization and decomposition of peptides P1–P4; (S2) isomeric ratio in the thermal equilibrium and at the respective PSS of P1–P4; Supplementary Figures: (S1–S4) UV/vis-spectra of P1–P4 at different pH values; (S5–S19) UV/vis kinetic measurements of thermal isomerization and decomposition of P1–P4; (S20–S22) MALDI-ToF measurements of P1, P2, and P4 at different pH values; (S23–S24) UV/vis spectra, measured (thermal equilibrium and PSS) and calculated (pure isomers) of P1–P4; (S25–S42) NMR data of compounds and peptides; (S43–S46) HPLC and MS data of peptides (PDF)
