# Supplementary material for: Spiropyran as Building Block in Peptide Synthesis and Modulation of Photochromic Properties
Source: Org Lett. 2024 Dec 2;26(49):10542–7. doi: 10.1021/acs.orglett.4c03929 (PMC11650765; doi:10.1021/acs.orglett.4c03929)
Supplement: Supplementary file 1 — ol4c03929_si_001.pdf [file ol4c03929_si_001.pdf]

Supporting Information

Spiropyran as building block in peptide synthesis and modulation of photochromic properties

André Paschold[1], Niclas Starke[1], Sven Rothemund[2], Wolfgang H. Binder[1]

[1] Macromolecular Chemistry, Institute of Chemistry, Faculty of Natural Science II, Martin Luther University Halle Wittenberg, von-Danckelmann-Platz 4, 06120 Halle (Germany)

[2]Core Unit Peptide – Technologies, University of Leipzig Medical Center, Liebigstraße 21 04103 Leipzig (Germany)

wolfgang.binder@chemie.uni-halle.de

SUPPORTING INFORMATION

Contents

1. Experimental procedures 2
2. Supplementary tables 5
3. Synthetis procedures & analytical data 6
4. Supplementary figures 17
5. NMR data of compounds and peptides and LC-MS of peptides 32

1. Experimental procedures

1.1. Chemicals

All technical solvents were distilled prior use. Air- and moisture- sensitive reactions were carried out in flame-dried glassware under atmospheric pressure of nitrogen. 1,3,3-Trimethyl-2-methylenindolin and 4-hydrazino benzoic acid were purchased from TCI Deutschland GmbH. NaOH and $NaNO_3$ were purchased from Gruessing GmbH. H_2SO_4 Boc_2O, iodomethane, citric acid, sodium citrate, NaH_2PO_4, 2-hydroxy-5-nitrobenzaldehyd, and PtO_2 were purchased from Sigma Aldrich. $SnCl_2$ and 3-methyl-2-butanone were purchased from Merck GmbH, triethlamin (TEA) and Na_2HPO_4 from Alfa Aesar, Fmoc-Cl from Fluorochem, 3-fomyl-4-hydroxybezoic acid from abcr GmbH, Ac_2O from VWR, and Celite® from Carl Roth GmbH.

The phosphate buffer for pH 7.4 was made as a mixture of an aqueous 50 mM Na_2HPO_4 and 50 mM NaH_2PO_4-solution. The citrate buffer for pH 2.5 was made as a mixture of an aqueous 100 mM citric acid and a 100 mM sodium citrate solution. Each buffer solution was adjusted with a NaOH- and HCl-solution to the correct pH measured with a Titroline 7500 KF from SI Analytics.

1.2. Instrumentation and Analysis

ESI-ToF-MS measurements were performed on a Bruker Daltonics microTOF via direct injection at a flow rate of 180 µL h^{-1} in positive mode with an acceleration voltage of 4.5 kV. Samples were prepared by dissolving in either LC-MS grade THF, LC-MS grade acetonitrile or LC-MS grade methanol or a mixture thereof. The instrument was calibrated using the ESI-L low concentration tuning mix from Agilent Technologies (product no. G1969-85000). The software Data Analysis (version 4.0) was used for data evaluation.

MALDI-ToF-MS measurements were performed on a Bruker Autoflex III system (Bruker Daltonics) using a nitrogen laser operating at a wavelength of λ = 337 nm in reflection mode. The used matrix:analyte ratio was 1:1 and 1 µL of the solution was spotted on the MALDI target. The peptide samples were either dissolved or suspended in MeCN + 0.1% trifluoroacetic acid (TFA) with a concentration of 20 mg/mL. Data evaluation was carried out via flexAnalysis software (3.4) and simulation of the isotopic pattern was performed by Data Analysis software (version 4.0).

NMR spectra were measured on an Agilent Technologies 400 MHz VNMRS and 500 MHz DD2 at 27°C. Chemical shifts (δ) are reported in ppm and referred to the solvent residual signal ($CDCl_3$ 7.26 ppm for ^1H and 77.0 ppm for ^{13}C, DMSO-d_6 2.50 ppm for ^1H and 39.52 ppm for ^{13}C, THF-d_8 1.72 and 3.58 ppm for ^1H and 67.2 and 25.3 ppm for ^{13}C, and D_2O 4.66 ppm for 1H). The following abbreviations were used for ^1H- and ^{13}C-NMR peaks assignment: s = singlet, d = doublet, t = triplet, td = triplet of doublet, m = multiplet.

ATR-IR spectra were measured on a Bruker Tensor Vertex 70 equipped with a Golden Gate Heated Diamond ATR Top-plate.

UV/VIS-absorption measurements were performed on a Perkin Elmer LAMBDA 365 UV/Vis Spectrophotometer using Helma analytics quartz glass cuvettes (d = 10 mm). Temperature control was achieved using the Perkin Elmer Peltier System L365. To describe the kinetic processes, the kinetic measurements were fitted according to a first-order reaction. In the measurements at 4 °C condensation of water occurred at the cuvette walls during the measurements. The respective parts during the kinetic measurements were excluded for the fit.

MC-to-SP-isomerization was achieved by irradiation of the respective peptide dissolved in a buffered aqueous solution in a quartz glass cuvette (d = 10 mm) under stirring with light with a distinct wavelength, whereby the light source was placed 1 cm away from the cuvette. Green light (peak at 525 nm) was generated using a LED (2 W, UHP-T-520-DI, Prizmatix) together with a driver (Ultra-High-Power LED Controller, Prizmatix). For light with a wavelength of 405 nm a mounted LED (1.3 W, M405L4, Thorlabs) was used together with a LED-driver (LEDD1B, Thorlabs).

High-performance liquid chromatography (HPLC) was performed on a Hitachi Chromaster (VWR) with the modules 5430 Detector, 5310 Column Oven, 5260 Autosampler, 5160 Pump. The mobile phases were water (eluent A) and acetonitrile (eluent B), respectively, each containing 0.1% formic acid. Samples were eluted for 60 min with a linear gradient, which had to be adjusted for each peptide individually. As column a Atlatis T3 5 µm (Waters Corporation) was used. The oven temperature was set to 20 °C and the autosampler temperature to 4 °C.

Solid-phase peptide synthesis was utilized on an automated peptide synthesizer MultiPep RS (Intavis AG, Koeln, Germany) using standard Fmoc-chemistry and preloaded resin. Standard coupling of all protected natural amino acids were performed as single couplings in dimethylformamid (DMF) using 5 equivalents of amino acids and 2-(6-chloro-1-H-benzotriazole-1-yl)-1,1,3,3-tetramethylaminium hexafluorophosphate (HCTU) as coupling reagents and 10 equivalents of N-methyl-morpholine (NMM) as base for 1 h at room temperature. Special building groups, such as FASC-Fmoc **1a** or SAFC-Fmoc **2a**, were coupled with 3 equivalents using *N,N'*-diisopropylcarbodiimide (DIC) and N-Hydroxybenzotriazole (HOBT) in DMF/NMP at room temperature and with gentle shaking in the dark overnight. The N-terminal Fmoc protecting group was removed by washing the resin with 20% piperidine for 20 min. The final side chain deprotection and cleavage from the resin employed a mixture of trifluoroaceticacid and water (90:10 Vol%) with gentle agitation for 2h at room temperature.

The crude peptides were purified to >95% purity using preparative RP-HPLC (Gilson, Limburg, Germany). For both analytical and preparative use, the mobile phases were water (eluent A) and acetonitrile (eluent B), respectively, each containing 0.1% TFA. Samples were eluted with a linear gradient from 5% B to 95% B in 15 min for analytical runs and in 90 min for preparative runs on a

semiprepative PLRP-S column (Agilent Technologies, 300x25mm, 8um). Finally, all peptides were characterized by analytical HPLC Dionex Ultimate 3000 (Thermo Scientific, Germany) using a PLRP-S column (Agilent Technologies, 150x4.6mm, 3um) and MALDI-MS (Bruker Microflex LT, Bremen, Germany), which gave the expected $[M+H]^+$ mass peaks.

2. Supplementary tables

Table S1. Peptide primary sequences, the half-life time $\tau_{1/2}$ and rate constant *k* of the thermal back-isomerization from SP-to-MC and, the half-life time $\tau_{1/2}$ and rate constant *k* of the hydrolysis of the MC-form at different temperatures and pH values.

**peptide**	**pH**	**T [°C]**	**thermal isomerization**		**decomposition**	
			**$\tau_{1/2}$ [min]**	***k* [min^{-1}]**	**$\tau_{1/2}$ [min]**	***k* [min^{-1}]**
P1	7.4	37	3.7 ± 0.1	0.185	68.7 ± 0.1	0.101
		4	171.4 ± 0.3	0.0040	4418 ± 11	0.000156
	2.5	37	10.7 ± 0.0	0.0645	15620 ± 21	4.44 * 10^{-5}
		4	1155 ± 0	0.000600	-	-
P2	7.4	37	2.9 ± 0.0	0.237	427.8 ± 2.1	0.00162
		4	321.4 ± 0.3	0.00216	n.d.	n.d.
	2.5	37	11.3 ± 0.0	0.0613	18733 ± 10	3.70 * 10^{-5}
		4	1151 ± 2	0.000602	n.d.	n.d.
P3	7.4	37	6.0 ± 0.1	0.116	75.3 ± 0.2	0.00921
		4	358.9 ± 0.2	0.00193	n.d.	n.d.
	2.5	37	15.8 ± 0.0	0.440	n.d.	n.d.
		4	2070 ± 1	0.000335	n.d.	n.d.
P4	2.5	37	1.7 ± 0.0	0.429	2072 ± 1	0.000334
	1	37	10.04 ± 0.2	0.0666	-	-
		4	384.9 ± 0.9	0.00180	-	-

n.d. – not determined.

Table S2. Isomeric ratio in the thermal equilibrium and the SP-PSS at pH 2.5 (P1 – P3) and pH 1 (P4).

**Peptide**	**ratio thermal equilibrium [MC:SP]**	**ratio PSS [MC:SP] (wavelength)**
P1	98:2	4:96 (520 nm)
P2	98:2	3:97 (520 nm)
P3	98:2	5:95 (520 nm)
P4	98:2	49:51 (405 nm)<br>38:62 (520 nm)

3. Synthetis procedures & analytical data

Synthesis strategy 1

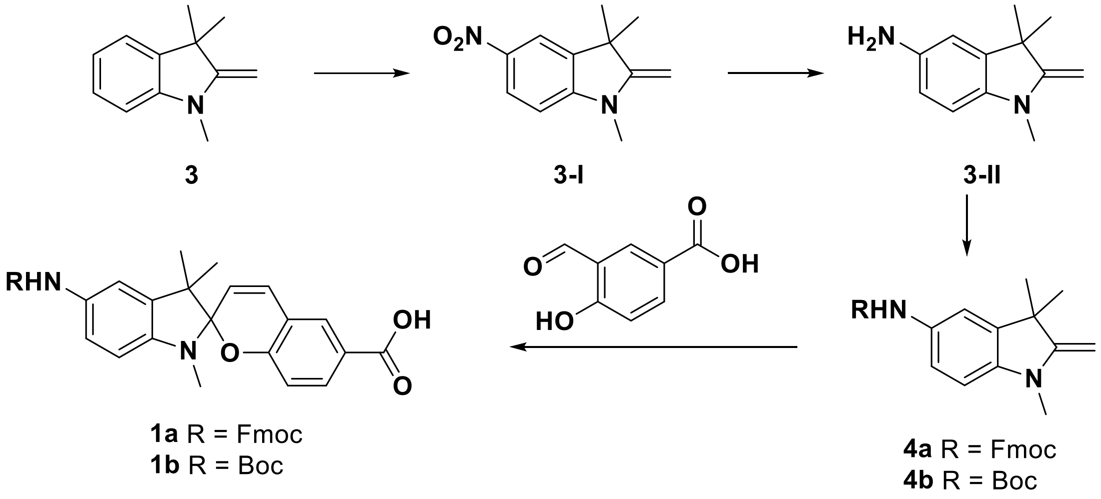

Scheme S1. Synthesis pathway for spiropyrans **1a** and **1b**.

The overall yield over 4 steps was for **1a** 4% and for **1b** 18%, respectively. Especially **3-II, 4a,** and **4b** are not completely stable at air and should therefore be processed faster.

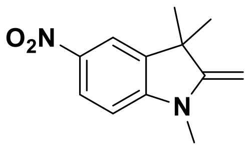

Synthesis of 3-I

1,3,3-Trimethyl-2-methylenindolin (10 g, 57.7 mmol, 1 eq.) was slowly added to 60 mL concentrated H_2SO_4 and cooled down to 0 °C. Sodiumnitrate (7.9 g, 63.5 mmol, 1.1 eq.) was added in small portions and the reaction mixture is stirred for 1 h. The solution was poured onto 100 g ice and neutralized with solid Na_2CO_3. The formed precipitate was filtrated and recrystallized (heated with an oil bath) in a mixture of acetone/water(1:1). **3-I** was obtained as a darkred solid. (7.3 g, 33.2 mmol, 58%).

Analytical data match reported literature values.[1]

R_f = 0.74 (hexane:EE / 3:2).

^{1}H-NMR (500 MHz, $CDCl_3$): δ = 8.12 (dd, *J* = 8.8, 2.3 Hz, 1H), 7.92 (d, *J* = 2.3 Hz, 1H), 6.51 (d, *J* = 8.8 Hz, 1H), 4.16–4.06 (m, 2H), 3.12 (s, 3H), 1.36 (s, 6H).

^{13}C-NMR (125 MHz, $CDCl_3$): δ = 161.2, 151.5, 140.0, 138.2, 126.2, 118.1, 103.9, 78.9, 43.4, 29.7, 29.0.

MS (ESI) *m/z*: $[M+Li]^+$ Calcd. for $[C_{12}H_{14}N_2O_2Li]^+$ 225.12; Found 225.12.

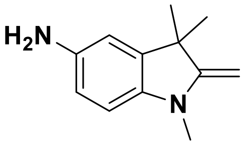

Synthesis of 3-II

3-I (2 g, 9.1 mmol, 1 eq.) and dry tin(II) chloride (10.4 g, 55 mmol, 6 eq.) were dissolved in 60 mL concentrated aqueous hydrogen chloride solution and refluxed (heated with an oil bath) for 2 h. The reaction solution was alkalinized and extracted three times with 60 mL Et_2O. The organic phases were combined and the solvent removed *in vacuo*. The residue was recrystallized in hexane (heated with an oil bath) and **3-II** was obtained as a green solid (1 g, 5.3 mmol, 58%).

Analytical data match reported literature values. [2]

R_f = 0.1 (DCM:MeOH + 0.1% FA / 9:1).

^{1}H-NMR (500 MHz, $CDCl_3$): δ = 6.56 (d, *J* = 2.3 Hz, 1H), 6.51 (dd, *J* = 8.1, 2.3 Hz, 1H), 6.35 (d, *J* = 8.0 Hz, 1H), 3.79–3.72 (m, 2H), 3.35 (s, 2H), 2.99 (s, 3H), 1.33 (s, 6H).

^{13}C-NMR (125 MHz, $CDCl_3$): δ = 163.4, 139.7, 138.9, 138.5, 114.1, 111.3, 105.1, 71.5, 44.4, 29.9, 29.0.

MS (ESI) *m/z*: $[M+H]^+$ Calcd. for $[C_{12}H_{17}N_2]^+$ 189.14; Found 189.14.

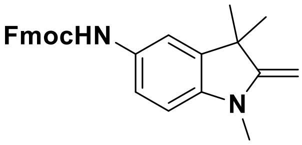

Synthesis of 4a

To a mixture of **3-II** (0.5 g, 2.7 mmol, 1 eq.) and Fmoc-Cl (0.82 g, 3.2 mmol, 1.2 eq.) were added 4 mL water and the suspension was stirred at 60 °C (heated with an oil bath) for 2 h. The reaction suspension was neutralized with saturated aqueous $NaHCO_3$ solution and extracted three times with 60 mL DCM. The organic phases were combined and the solvent removed *in vacuo*. The residue was purified by flash chromatography on normal phase silica gel (solvent A: DCM + 0.1% FA, solvent B: MeOH + 0.1% FA, 95% A) and **4a** was obtained as a lightpurple solid. (0.38 g, 0.93 mmol, 35%).

R_f = 0.16 (DCM:MeOH + 0.1% FA / 9:1).

^{1}H-NMR (500 MHz, $CDCl_3$): δ = 7.78 (d, *J* = 7.5 Hz, 2H), 7.69–7.52 (m, 2H), 7.41 (t, *J* = 7.4 Hz, 2H), 7.37–7.29 (m, 2H), 7.24–7.15 (m, 1H), 7.05 (s, 1H), 6.52 (s, 1H), 6.45 (d, *J* = 8.3 Hz, 1H), 4.50 (d, *J* = 6.8 Hz, 2H), 4.28 (t, *J* = 6.9 Hz, 1H), 3.84 (q, *J* = 2.0 Hz, 2H), 3.02 (s, 3H), 1.34 (s, 6H). ^{13}C-NMR (125 MHz, $CDCl_3$): δ = 162.8, 154.1, 143.9, 143.2, 141.3, 138.3, 129.2, 127.7, 127.1, 125.0, 120.0, 119.2, 114.9, 104.6, 73.1, 66.7, 47.2, 44.3, 29.9, 28.9.

IR (ATR): ν = 3303w, 2959w, 1697s, 1499s, 1208s cm^{-1}.

UV/VIS (c = 0.06 µmol mL^{-1} in MeOH): $\lambda_{max,1}$ = 289.2, $\lambda_{max,2}$ = 277.3, $\lambda_{max,3}$ = 265.3 nm.

HR-MS (ESI) *m/z*: $[M+H]^+$ Calcd. for $[C_{27}H_{27}N_2O_2]^+$ 411.2106; Found: 411.2119.

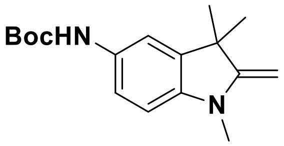

Synthesis of 4b

3-II (585 mg, 3.1, mmol, 1 eq.) were dissolved in 40 mL THF. Boc_2O (680 mg, 3.1 mmol, 1 eq.) and TEA (440 µL, 3.1 mmol, 1 eq.) were added under a nitrogen atmosphere and the reaction mixture was stirred at ambient temperature for 18 h. The solvent was removed *in vacuo* and the residue was dissolved in 50 mL DCM. The solution was extracted three times with 50 mL of an aqueous solution with 5% acetic acid. The combined aqueous phases were washed two times with 50 mL Et_2O and the solution was made alkaline (pH 9 – 10) with $NaHCO_3$. The aqueous solution was extracted three times with 50 mL DCM. The combined organic phases were dried over Na_2SO_4 and the solvent was removed *in vacuo*. The residue of **4b** (yellowish oil, 760 mg) was used without further purification.

R_f = 0.39 (DCM:MeOH / 19:1).

^{1}H-NMR (400 MHz, $CDCl_3$) δ = 7.17 (s, 1H), 7.02 – 6.90 (m, 1H), 6.41 (d, *J* = 8.3 Hz, 1H), 6.26 (s, 1H), 3.79 (q, *J* = 2.0 Hz, 2H), 2.99 (s, 3H), 1.49 (s, 9H), 1.31 (s, 6H).
^{13}C-NMR (100 MHz, $CDCl_3$) δ = 163.0, 155.0, 142.7, 138.2, 129.7, 118.7, 114.7, 104.5, 79.6, 72.7, 44.3, 29.9, 28.9, 28.4.
MS (ESI) *m/z*: $[M+H]^+$ Calcd. for $[C_{17}H_{25}N_2O_2]^+$ 289.19; Found 289.19.

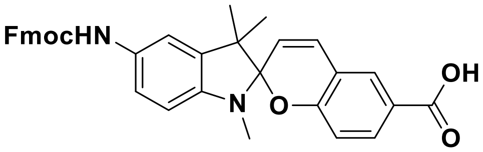

Synthesis of 1a

4a (0.3 g, 0.73 mmol, 1 eq.) and 3-formyl-4-hydroxybenzoic acid (0.16 g, 0.95 mmol, 1.3 eq.) were dissolved in 25 mL MeCN for 6 h and refluxed (heated with an oil bath) under a nitrogen atmosphere. The solvent was removed *in vacuo* and the residue was purified by flash chromatography on normal phase silica gel (solvent A: hexane + 0.1% FA, solvent B: EE + 0.1% FA, 60% A). **1a** was obtained as a redish solid (0.17 g, 0.3 mmol, 42%).

R_f = 0.45 (hexane:EE + 0.1% FA / 3:2).

^{1}H-NMR (500 MHz, DMSO-d_6): δ = 12.60 (s, 1H), 9.40 (s, 1H), 7.89 (d, J = 7.0 Hz, 2H), 7.79 (d, J = 2.2 Hz, 1H), 7.77 – 7.69 (m, 2H), 7.67 (dd, J = 8.5, 2.2 Hz, 1H), 7.41 (t, J = 7.4 Hz, 2H), 7.34 (t, J = 7.4 Hz, 2H), 7.23 (s, 1H), 7.15 (s, 1H), 7.10 (d, J = 10.5 Hz, 1H), 6.73 (d, J = 8.5 Hz, 1H), 6.49 (d, J = 8.3 Hz, 1H), 5.82 (d, J = 10.2 Hz, 1H), 4.41 (d, J = 6.9 Hz, 2H), 4.28 (t, J = 6.9 Hz, 1H), 2.60 (s, 3H), 1.17 – 1.07 (m, 6H).

^{13}C-NMR (125 MHz, DMSO-d_6): δ = 167.2, 158.1, 154.2, 144.3, 141.2, 137.3, 136.9, 131.8, 131.2, 129.5, 129.0, 128.1, 127.6, 125.6, 123.2, 120.6, 120.2, 118.9, 118.0, 114.9, 107.3, 105.7, 65.9, 52.1, 47.2, 40.6, 40.4, 40.3, 40.1, 39.9, 39.8, 39.6, 29.2, 26.1, 20.1.

IR (ATR): ν = 3338w, 2968br, 1693s, 1234m, 1208s cm^{-1}.

UV/VIS (c = 0.46 μmol mL^{-1} in MeOH): $\lambda_{max,1}$ = 443.7 nm.

HR-MS (ESI) *m/z*: [M+H]$^+$ Calcd. for $[C_{35}H_{31}N_2O_5]^+$ 559.2227; Found 559.2258.

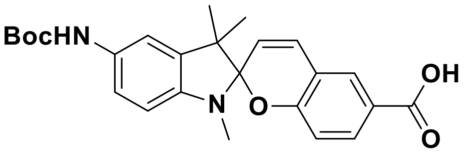

Synthesis of 1b

The unpurified residue of **7** (700 mg, ~2.43 mmol, 1 eq.) and 3-formyl-4-hydroxybenzoic acid (400 mg, 2.43 mmol, 1 eq.) were dissolved in 50 mL MeCN and the solution was refluxed (heated with an oil bath) for 4.5 h under a nitrogen atmosphere. The solvent was removed *in vacuo* and the residue was purified by flash chromatography in normal phase silica (solvent A: hexane + 0.1% FA, solvent B: EE + 0.1% FA, 60% A). **1b** was obtained as a redish solid (0.680 mg, 1.56 mmol, 64%).

R_f = 0.41 (hexane:EE + 0.1% FA / 3:2).

^{1}H-NMR (500 MHz, DMSO-d_6): δ = 12.65 (s, 1H), 8.97 (s, 1H), 7.80 (d, J = 2.3 Hz, 1H), 7.68 (dd, J = 8.5, 2.3 Hz, 1H), 7.25 (s, 1H), 7.18 – 6.99 (m, 2H), 6.74 (d, J = 8.6 Hz, 1H), 6.48 (d, J = 8.3 Hz, 1H), 5.83 (d, J = 10.3 Hz, 1H), 2.61 (s, 3H), 1.46 (s, 9H), 1.17 (s, 3H), 1.10 (s, 3H).

^{13}C-NMR (125 MHz, DMSO-d_6): δ = 166.7, 157.7, 153.1, 143.2, 136.3, 131.3, 129.0, 128.5, 122.7, 122.0, 119.8, 118.4, 117.5, 114.4, 113.6, 106.7, 105.3, 78.4, 51.6, 28.7, 28.2, 25.6, 19.7.

IR (ATR): ν = 3319w, 3069w, 2973br, 1683s, 1158s cm^{-1}.

HR-MS (ESI) *m/z*: [M+Na]$^+$ Calcd. for $[C_{25}H_{28}N_2O_5Na]^+$ 459.1890; Found 459.1909.

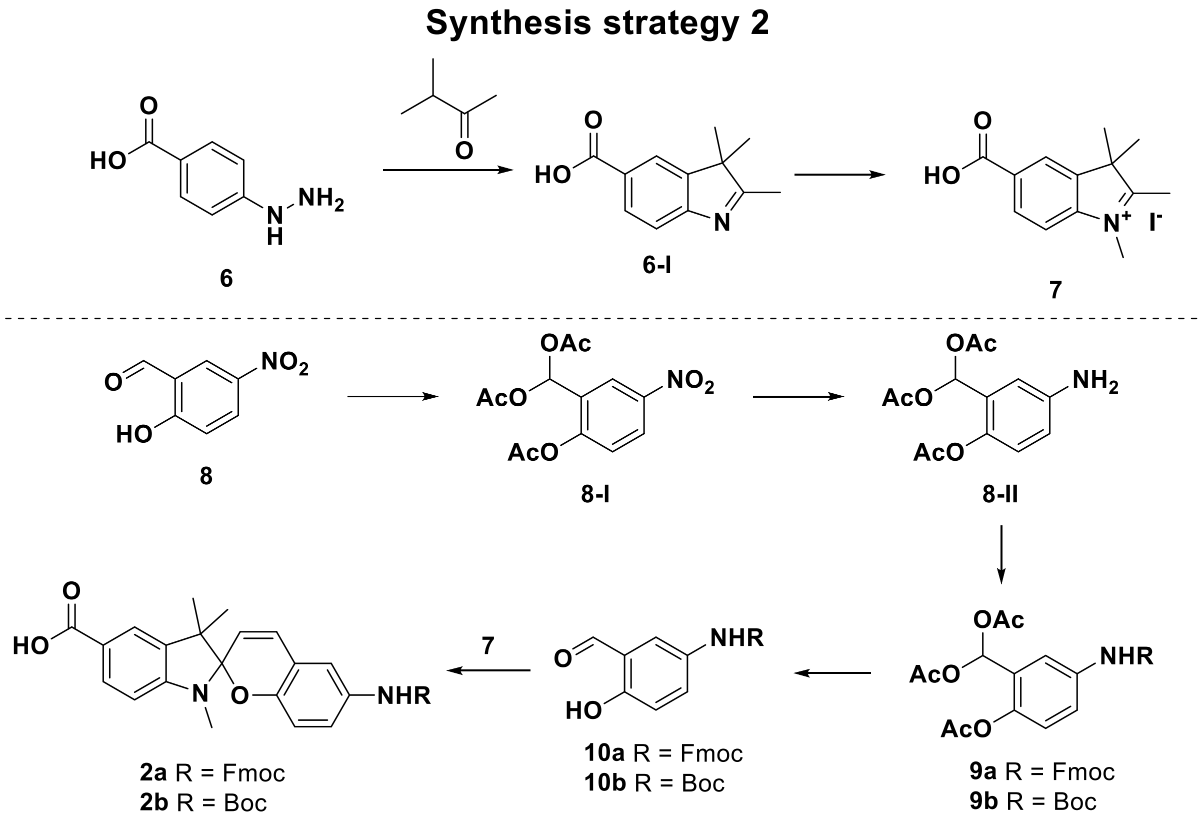

Scheme S2. Synthesis pathway for spiropyrans **2a** and **2b**.

The overall yield over 6 consecutive steps was for **2a** 4% and for **2b** 3%, respectively. Especially **8-II**, was not completely stable at air and should therefore be processed faster. Furthermore, the actual indole precursor was generated in the last step from **7** *in situ* through deprotonation. A more convenient method could be to produce and isolate the respective indole precursor in an additional step.

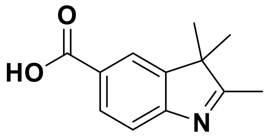

Synthesis of 6-I

4-Hydrazinobenzoic acid **6** (5 g, 33 mmol, 1 eq.) and 3-methyl-2-butanone (5 ml, 59 mmol, 1.8 eq.) were added to 50 mL acetic acid and refluxed (heated with an oil bath) for 16 h. The solvent was removed *in vacuo*, the residue redissolved in 50 mL DCM and neutralized with saturated aqueous $NaHCO_3$-solution. The combined organic phases were dried over Na_2SO_4 and the solvent was removed *in vacuo* to obtain **6-I** as an orange solid (1.3 g, 6.4 mmol, 19%).

Analytical data match reported literature values.[3]

R_f = 0.74 (DCM:MeOH + 0.1% FA / 9:1).

^{1}H-NMR (500 MHz, $CDCl_3$): δ = 12.76 (s, 1H), 7.97 (d, *J* = 1.6 Hz, 1H), 7.90 (dd, *J* = 8.1, 1.5 Hz, 1H), 7.49 (dd, *J* = 8.0, 1.2 Hz, 1H), 2.23 (s, 3H), 1.25 (s, 6H).

^{13}C-NMR (125 MHz, $CDCl_3$): δ = 192.1, 167.9, 157.9, 146.6, 130.0, 127.7, 123.2, 119.6, 53.9, 22.7, 15.8.

MS (ESI) *m/z*: $[M+H]^+$ Calcd. for $[C_{12}H_{14}NO_2]^+$ 204.10; Found 204.10.

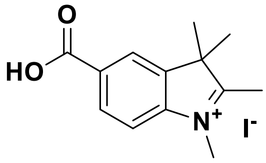

Synthesis of 7

6-I (5 g, 25 mmol, 1 eq.) was dissolved in 70 mL toluene and 35 mL MeCN under nitrogen atmosphere. Iodomethane (1.8 ml, 30 mmol, 1.2 eq.) was added and the reaction mixture was refluxed (heated with an oil bath) for 16 h. The formed precipitate was separated, washed with hexane and cold ethanol and dried. **7** was obtained as an orange-pinkish solid (2.9 g, 8.4 mmol, 34%).

Analytical data match reported literature values. [3]

R_f = 0.21 (DCM:MeOH + 0.1% FA / 9:1).

^{1}H-NMR (400 MHz, DMSO-*d*$_6$): δ = 8.35 (s, 1H), 8.17 (d, *J* = 8.4 Hz, 1H), 8.02 (d, *J* = 8.4 Hz, 1H), 3.98 (s, 3H), 2.80 (s, 3H), 1.55 (s, 6H).

^{13}C-NMR (100 MHz, DMSO-*d*$_6$): δ = 199.5, 166.9, 145.7, 142.4, 132.0, 130.8, 124.7, 115.8, 54.7, 35.5, 21.9, 15.0.

MS (ESI) *m/z*: $[M-I]^+$ Calcd. for $[C_{13}H_{16}NO_2]^+$ 218.12; Found 218.12.

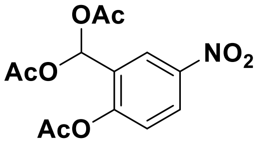

Synthesis of 8-I

2-Hydroxy-5-nitrobenzaldehyd **8** (2.5 g, 15 mmol, 1 eq.) was dissolved in 50 mL acetic anhydride. Three drops of concentrated H_2SO_4 were added and the solution was stirred for 1 h under nitrogen atmosphere at ambient temperature. The reaction mixture was poured in 500 mL water and stirred for 30 min. The formed precipitate was separated, washed with water and dried. **8-I** was obtained as white crystals (3.57 g, 11.5 mmol, 76%).

Analytical data match reported literature values.[4]

R_f = 0.23 (hexane:EE/ 8:2).

^{1}H-NMR (400 MHz, $CDCl_3$): δ = 8.53 (d, *J* = 2.8 Hz, 1H), 8.29 (dd, *J* = 8.9, 2.8 Hz, 1H), 7.93 (s, 1H), 7.35 (d, *J* = 8.9 Hz, 1H), 2.38 (s, 3H), 2.13 (s, 6H).

^{13}C-NMR (100 MHz, $CDCl_3$): δ = 168.3, 168.2, 152.7, 145.6, 129.6, 125.8, 124.4, 123.8, 84.1, 20.8, 20.6.

MS (ESI+, MeCN/MeOH): *m/z* = 318.081 $[M+Li]^+$. 318.0801

MS (ESI) *m/z*: $[M+Li]^+$ Calcd. for $[C_{13}H_{13}NO_8Li]^+$ 318.08; Found 318.08.

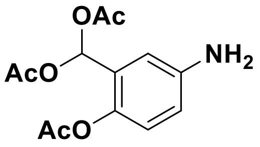

Synthesis of 8-II

8-I (1.5 g, 4.8 mmol, 1 eq.) was dissolved in 40 mL ethanol and PtO_2 (109 mg, 480 µM, 0.1 eq.) was added. The reaction suspension was stirred for 12 h under a hydrogen atmosphere (5 bar) at ambient temperature. The suspension was filtrated over Celite® and the solvent of the filtrate was removed *in vacuo*. The residue was purified by flash chromatography on normal phase silica gel (solvent A: hexane, solvent B: EE, 60% A) and **8-II** was obtained as a yellowish solid (0.95 g, 3.4 mmol, 70%).

Analytical data match reported literature values.[4]

R_f = 0.4 (hexane:EE / 1:1).

^{1}H-NMR (500 MHz, $CDCl_3$): δ = 7.79 (s, 1H), 6.95–6.83 (m, 2H), 6.69 (dd, *J* = 8.7, 2.8 Hz, 1H), 3.72 (s, 2H), 2.30 (s, 3H), 2.10 (s, 6H).

^{13}C-NMR (125 MHz, $CDCl_3$): δ = 170.1, 168.4, 144.6, 140.1, 128.1, 123.8, 117.0, 113.5, 85.4, 20.8, 20.7.

MS (ESI) *m/z*: $[M+Li]^+$ Calcd. for $[C_{13}H_{15}NO_6Li]^+$ 288.11; Found 288.11.

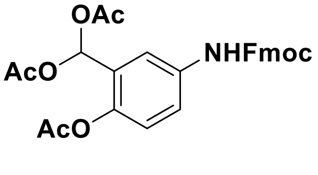

Synthesis of 9a

8-II (2 g, 7.1 mmol, 1 eq.) and Fmoc-Cl (2.2 g, 8.5 mmol, 1.2 eq.) were suspended in 10 mL water and stirred for 2h at 60 °C (heated with an oil bath). The reaction mixture was extracted three times with 60 mL EE and the combined organic phases were dried over Na_2SO_4. The solvent was removed *in vacuo*

and the residue was purified by flash chromatography on normal phase silica gel (solvent A: hexane, solvent B: EE, 80% A) to obtain **9a** as transparent crystals (1.45 g, 2.9 mmol, 41%).

R_f = 0.12 (hexane:EE / 4:1).

^{1}H-NMR (500 MHz, $CDCl_3$): δ = 7.85 (s, 1H), 7.79 (d, *J* = 7.6 Hz, 2H), 7.68 (s, 1H), 7.61 (d, *J* = 7.5 Hz, 2H), 7.44–7.40 (m, 3H), 7.33 (t, *J* = 7.4 Hz, 2H), 7.05 (d, *J* = 8.8 Hz, 1H), 6.74 (s, 1H), 4.56 (d, *J* = 6.6 Hz, 2H), 4.28 (t, *J* = 6.5 Hz, 1H), 2.32 (s, 3H), 2.09 (s, 6H).

^{13}C-NMR (125 MHz, $CDCl_3$): δ = 169.5, 168.4, 153.2, 143.8, 143.6, 141.4, 135.8, 128.4, 127.8, 127.2, 124.9, 123.8, 120.8, 120.1, 117.8, 85.0, 67.0, 47.1, 20.8, 20.7.

IR (ATR): ν = 3399m, 1749s, 1727s, 1543m, 1195s cm^{-1}.

UV/VIS (c = 0.05 µmol mL^{-1} /MeOH): $\lambda_{max,1}$ = 300, $\lambda_{max,2}$ = 289, $\lambda_{max,1}$ = 264, $\lambda_{max,1}$ = 244 nm.

HR-MS (ESI) *m/z*: $[M+Li]^+$ Calcd. for $[C_{28}H_{25}NO_8Li]^+$ 510.1740; Found 510.1724.

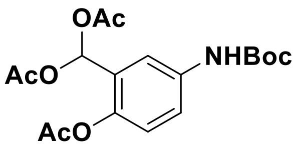

Synthesis of 9b

8-II (1 g, 3.6 mmol, 1 eq.) and Boc_2O (1.55 g, 7 mmol, 2 eq.) were dissolved in 10 mL DCM and stirred at ambient temperature for 12 h under a nitrogen atmosphere. The solvent was removed *in vacuo* and the residue was purified by flash chromatography on normal phase silica gel (solvent A: hexane, solvent B: EE, 80% A) to obtain **9b** as a transparent gel (0.57 g, 1.5 mmol, 42%).

Analytical data match reported literature value.[4]

R_f = 0.63 (hexane:EE / 3:2).

^{1}H-NMR (400 MHz, $CDCl_3$): δ = 7.84 (s, 1H), 7.64 (d, *J* = 2.5 Hz, 1H), 7.41 (d, *J* = 8.8, 2.7 Hz, 1H), 7.03 (d, *J* = 8.8 Hz, 1H), 6.52 (s, 1H), 2.31 (s, 3H), 2.09 (s, 6H), 1.52 (s, 9H).

^{13}C-NMR (100 MHz, $CDCl_3$): δ = 169.6, 168.4, 152.5, 143.3, 136.5, 128.2, 123.7, 120.7, 117.6, 85.1, 80.9, 28.3, 20.8, 20.7.

MS (ESI) *m/z*: $[M+Li]^+$ Calcd. for $[C_{18}H_{23}NO_8Li]^+$ 388.16; Found 388.16.

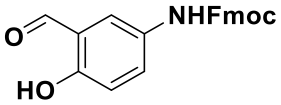

Synthesis of 10a

9a (0.1 g, 0.2 mmol, 1 eq.) was dissolved in 20 mL ethanol and 1 mL of concentrated H_2SO_4 was added. The solution was refluxed (heated with an oil bath) for 2 h and neutralized with a saturated aqueous $NaHCO_3$-solution. The solution was extracted three times with 50 mL Et_2O and the combined organic phases were dried over Na_2SO_4. The solvent is removed *in vacuo* and **10a** was obtained as beige crystals (52 mg, 0.14 mmol, 73%).

R_f = 0.28 (hexane:EE / 4:1).

^{1}H-NMR (400 MHz, THF-d_8): δ = 10.58 (s, 1H), 9.91 (s, 1H), 8.82 (s, 1H), 7.88 (s, 1H), 7.79 (d, *J* = 7.5 Hz, 2H), 7.67 (d, *J* = 7.3 Hz, 2H), 7.51 (s, 1H), 7.36 (t, *J* = 7.5 Hz, 2H), 7.28 (t, *J* = 7.4 Hz, 2H), 6.85 (d, *J* = 8.9 Hz, 1H), 4.55 (d, *J* = 6.3 Hz, 2H), 4.26 (t, *J* = 6.2 Hz, 1H).

^{13}C-NMR (100 MHz, THF-d_8): δ = 196.8, 157.8, 154.3, 145.0, 142.2, 132.8, 128.2, 128.0, 127.6, 125.5, 122.6, 121.5, 120.5, 118.0, 66.6, 48.1.
IR (ATR): ν = 3294m, 1693s, 1658s, 1243s, 732m cm^{-1}.

UV/VIS (c = 0.07 µmol mL^{-1} /MeOH): $\lambda_{max,1}$ = 356, $\lambda_{max,2}$ = 256, $\lambda_{max,3}$ = 235 nm.

HR-MS (ESI) *m*/*z*: $[M+Na]^+$ Calcd. for $[C_{22}H_{18}NO_4Na]^+$ = 382.1055; Found 382.1052.

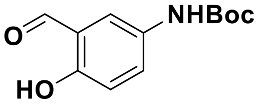

Synthesis of 10b

9b (0.5 g, 1.3 mmol, 1 eq.) was dissolved in 10 mL methanol and 5 g NaOH dissolved in 5 mL water were added. The solution was refluxed (heated with an oil bath) for 3 h and neutralized with an aqueous 1 M HCl-solution. The solution was extracted three times with 20 mL EE and the combined organic phases were dried over Na_2SO_4. The solvent was removed *in vacuo* and the residue was purified by flash chromatography on normal phase silica gel (solvent A: hexane, solvent B: EE, 90% A) to obtain **10b** as a yellow solid (0.21 g, 890 µmol, 45%).

Analytical data match reported literature values.[4]

R_f = 0.45 (hexane:EE / 4:1).

^{1}H-NMR (400 MHz, $CDCl_3$): δ = 10.76 (s, 1H), 9.86 (d, *J* = 0.5 Hz, 1H), 7.83 (s, 1H), 7.29 (dd, *J* = 8.9, 2.7 Hz, 1H), 6.92 (d, *J* = 8.9 Hz, 1H), 6.45 (s, 1H), 1.52 (s, 6H).

^{13}C-NMR (100 MHz, $CDCl_3$): δ = 196.5, 157.6, 153.0, 130.8, 128.4, 123.3, 120.2, 118.0, 80.9, 28.3.

MS (ESI) *m/z*: $[M+Li]^+$ Calcd. for $[C_{12}H_{15}NO_4Li]^+$ 244.12; Found 244.11.

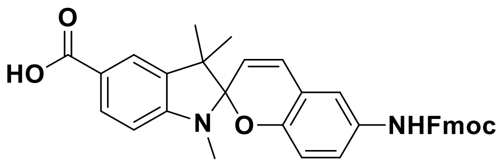

Synthesis of 2a

10a (0.3 g, 0.83 mmol, 1 eq.) and **7** (0.58 g, 1.7 mmol, 2 eq.) were dissolved in 100 mL ethanol under a nitrogen atmosphere and TEA (0.47 mL, 3.3 mmol, 4 eq.) was added slowly. The solution was refluxed (heated with an oil bath) for 3 h and neutralized with an aqueous 1 M HCl-solution. The solution was extracted three times with 60 mL EE and the combined organic phases were dried over Na_2SO_4. The solvent was removed *in vacuo* and the residue was purified by flash chromatography on normal phase silica gel (solvent A: DCM, solvent B: MeOH, 98% A) to obtain **2a** as a red solid (0.12 g, 0.21 mmol, 26%).

R_f = 0.41 (DCM:MeOH / 19:1).

^{1}H-NMR (400 MHz, THF-d_8): δ = 12.83 (s, 1H), 8.64 (s, 1H), 7.87 (dd, *J* = 8.2, 1.7 Hz, 1H), 7.80 (d, *J* = 7.6 Hz, 2H), 7.72 (d, *J* = 1.7 Hz, 1H), 7.68 (d, *J* = 7.4 Hz, 2H), 7.43 – 7.34 (m, 3H), 7.29 (td, *J* = 7.4, 1.2 Hz, 2H), 7.10 (d, *J* = 8.7 Hz, 1H), 6.93 (d, *J* = 10.2 Hz, 1H), 6.58 (d, *J* = 8.7 Hz, 1H), 6.53 (d, *J* = 8.2 Hz, 1H), 5.76 (d, *J* = 10.2 Hz, 1H), 4.51 (d, *J* = 6.5 Hz, 2H), 4.27 (t, *J* = 6.4 Hz, 1H), 2.77 (s, 3H), 1.33 (s, 3H), 1.17 (s, 3H).

^{13}C-NMR (100 MHz, THF-d_8): δ = 167.6, 154.2, 152.6, 150.5, 145.1, 142.2, 137.3, 133.3, 131.6, 130.6, 128.1, 127.6, 125.6, 123.8, 122.3, 120.5, 119.8, 119.4, 117.5, 115.3, 106.2, 104.8, 66.5, 51.8, 48.2, 28.7, 25.9, 20.3.

IR (ATR): ν = 3303br, 3064br, 1671s, 1583s, 1199s cm^{-1}.
UV/VIS (c = 0.09 µmol mL^{-1} /MeOH): $\lambda_{max,1}$ = 474, $\lambda_{max,2}$ = 378, $\lambda_{max,3}$ = 299, $\lambda_{max,4}$ = 264 nm.
HR-MS (ESI) *m*/*z*: $[M+H]^+$ Calcd. for $[C_{35}H_{31}N_2O_5]^+$ 559.2227; Found: 559.2207.

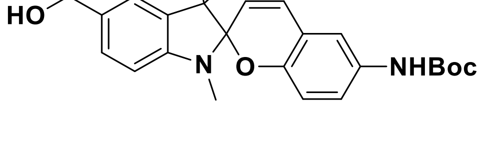

Synthesis of 2b

10b (0.5 g, 2.1 mmol, 0.5 eq.) and **7** (1.45 g, 4.2 mmol, 2 eq.) were dissolved in 100 mL ethanol under a nitrogen atmosphere and TEA (1.2 mL, 8.4 mmol, 4 eq.) was added slowly. The solution was refluxed (heated with an oil bath) for 3 h and neutralized with an aqueous 1 M HCl-solution. The solution was

extracted three times with 60 mL EE and the combined organic phases were dried over Na_2SO_4. The solvent was removed *in vacuo* and the residue was purified by flash chromatography on normal phase silica gel (solvent A: hexane, solvent B: EE, 20% A) to obtain **2b** as a pink solid (0.3 g, 0.7 mmol, 33%).

R_f = 0.35 (DCM:MeOH / 19:1).

^{1}H-NMR (400 MHz, DMSO-d_6): δ = 12.25 (s, 1H), 9.10 (s, 1H), 7.76 (dd, *J* = 8.2, 1.7 Hz, 1H), 7.61 (d, *J* = 1.6 Hz, 1H), 7.34 (s, 1H), 7.04 (dd, *J* = 8.8, 2.4 Hz, 1H), 6.98 (d, *J* = 10.2 Hz, 1H), 6.59 (dd, *J* = 8.4, 6.2 Hz, 2H), 5.75 (d, *J* = 10.2 Hz, 1H), 2.70 (s, 3H), 1.44 (s, 3H), 1.21 (s, 3H), 1.08 (s, 3H).

^{13}C-NMR (100 MHz, DMSO-d_6): δ = 167.9, 153.4, 153.0, 149.3, 136.8, 132.9, 131.1, 130.2, 123.3, 121.4, 120.9, 119.5, 118.6, 117.4, 114.8, 106.3, 104.0, 79.2, 51.3, 28.9, 28.6, 26.0, 20.2.

IR (ATR): ν = 3448w, 2980m, 1724s, 1667s, 1155s cm^{-1}.

UV/VIS (c = 0.03 µmol mL^{-1} /MeOH): $\lambda_{max,1}$ = 301, $\lambda_{max,2}$ = 233 nm.

HR-MS (ESI) *m/z*: $[M+Li]^+$ Calcd. $[C_{25}H_{28}N_2O_5Li]^+$ 443.2158; Found: 443.2121.

4. Supplementary figures

UV/Vis-spectra

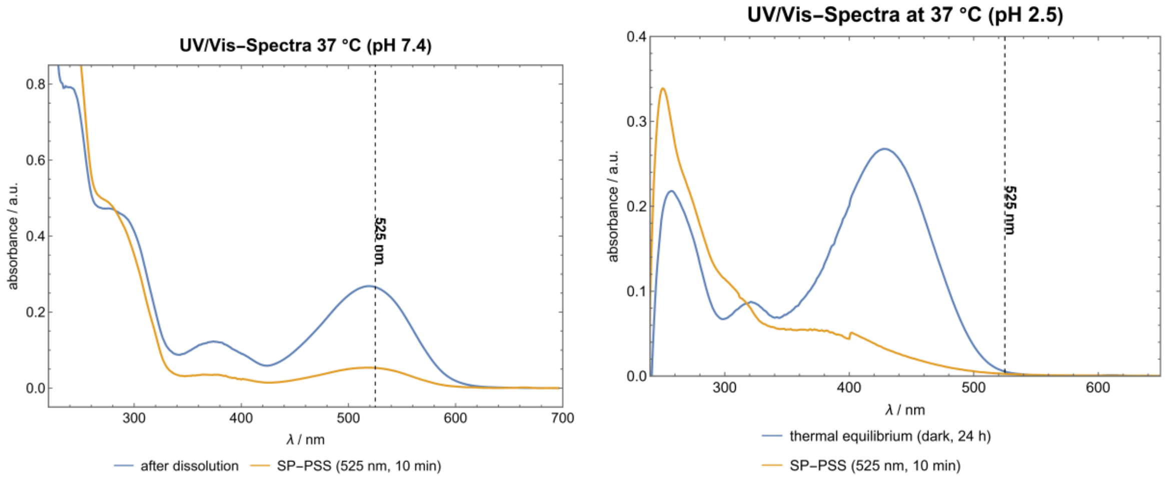

Figure S1. UV/Vis-spectra of **P1** at 37 °C and different pH values: pH 7.4 (left, 55 µM), pH 2.5 (right, 13 µM).

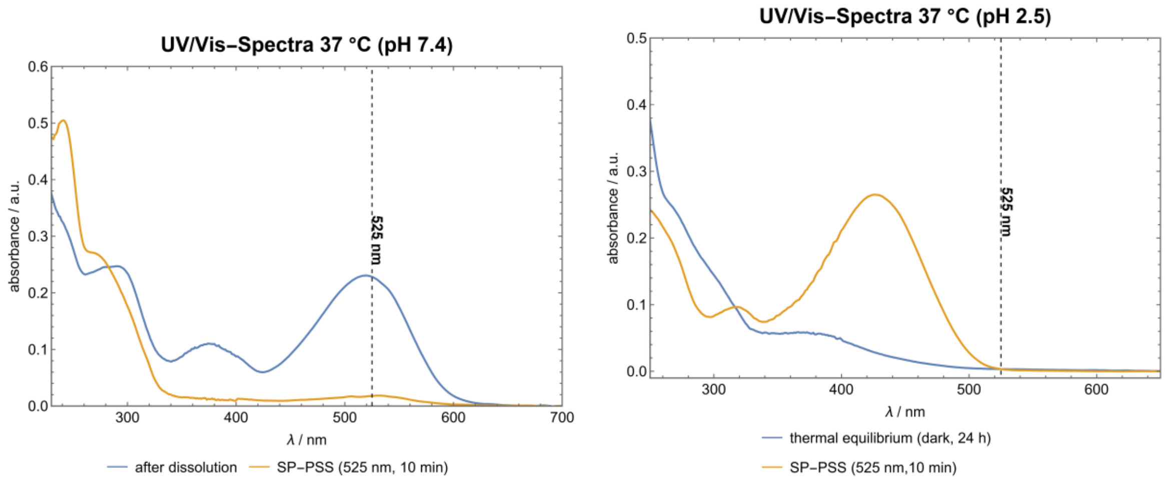

Figure S2. UV/Vis-spectra of **P2** at 37 °C and different pH values: pH 7.4 (left, 30 µM), pH 2.5 (right, 16 µM).

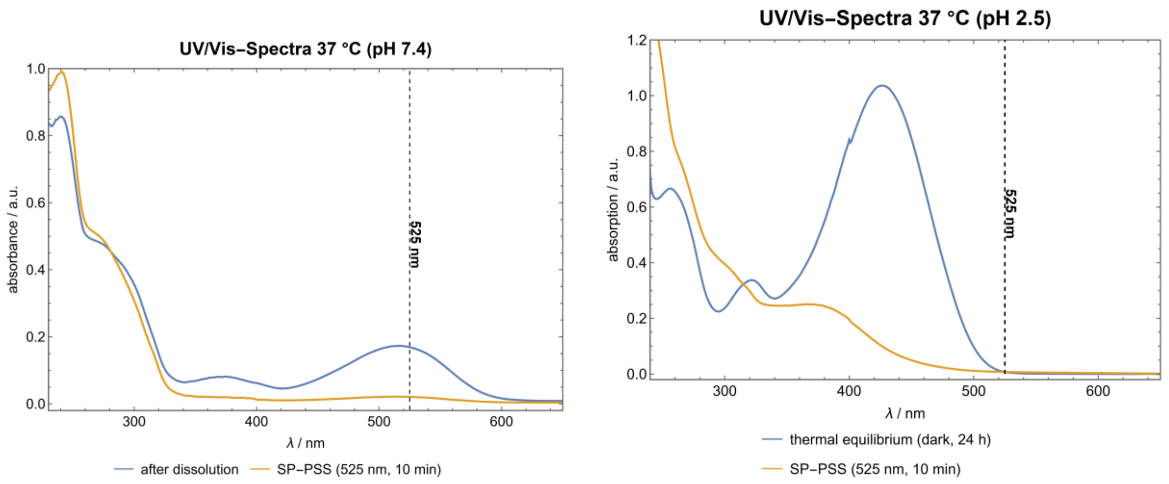

Figure S3. UV/Vis-spectra of **P3** at 37 °C and different pH values: pH 7.4 (left, 50 µM), pH 2.5 (right, c = 55 µM).

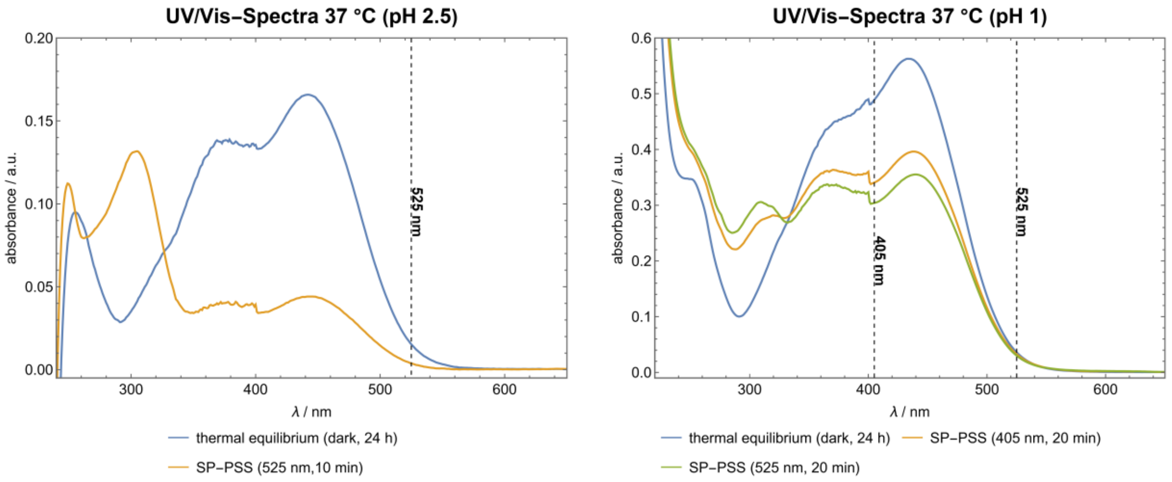

Figure S4. UV/Vis-spectra of **P4** at 37 °C and different pH values: pH 2.5 (left, 15 µM), pH 1 (right, 44 µM).

<u>Kinetics</u>

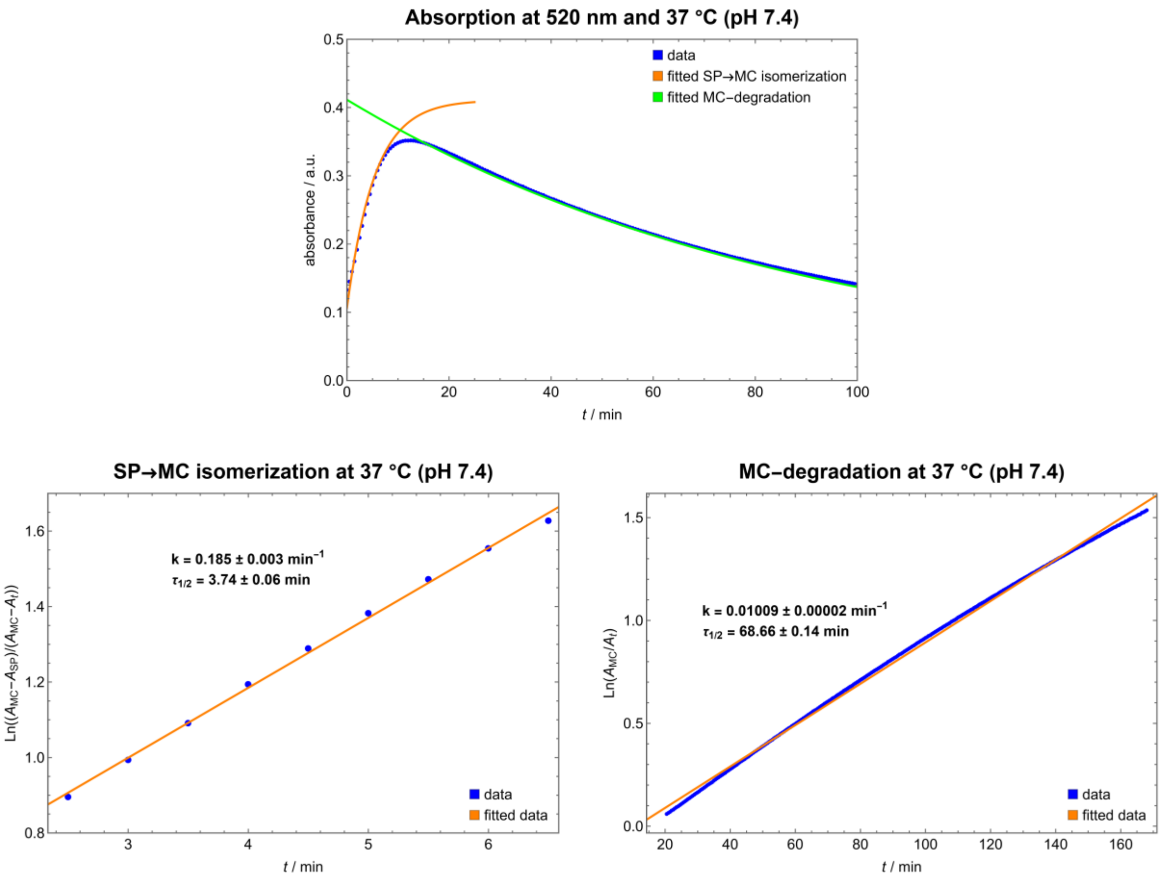

Figure S5. Kinetic measured at 520 nm (top) of SP-to-MC isomerization (rate constant *k*, lower left) and hydrolysis (rate constant *k*, lower right) of **P1** (55 µM) at pH 7.4 and 37 °C.

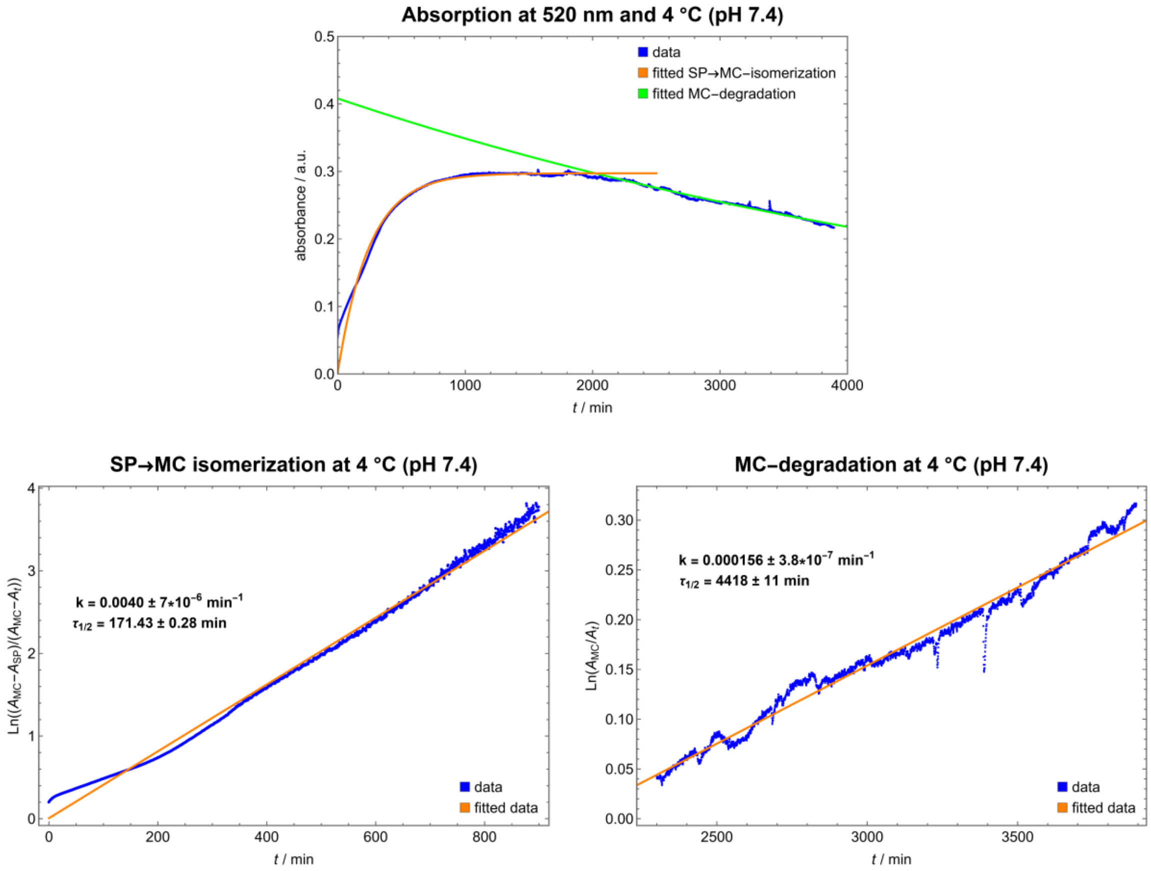

Figure S6. Kinetic measured at 520 nm (top) of SP-to-MC isomerization (rate constant *k*, lower left) and hydrolysis (rate constant *k*, lower right) of **P1** (40 µM) at pH 7.4 and 4 °C.

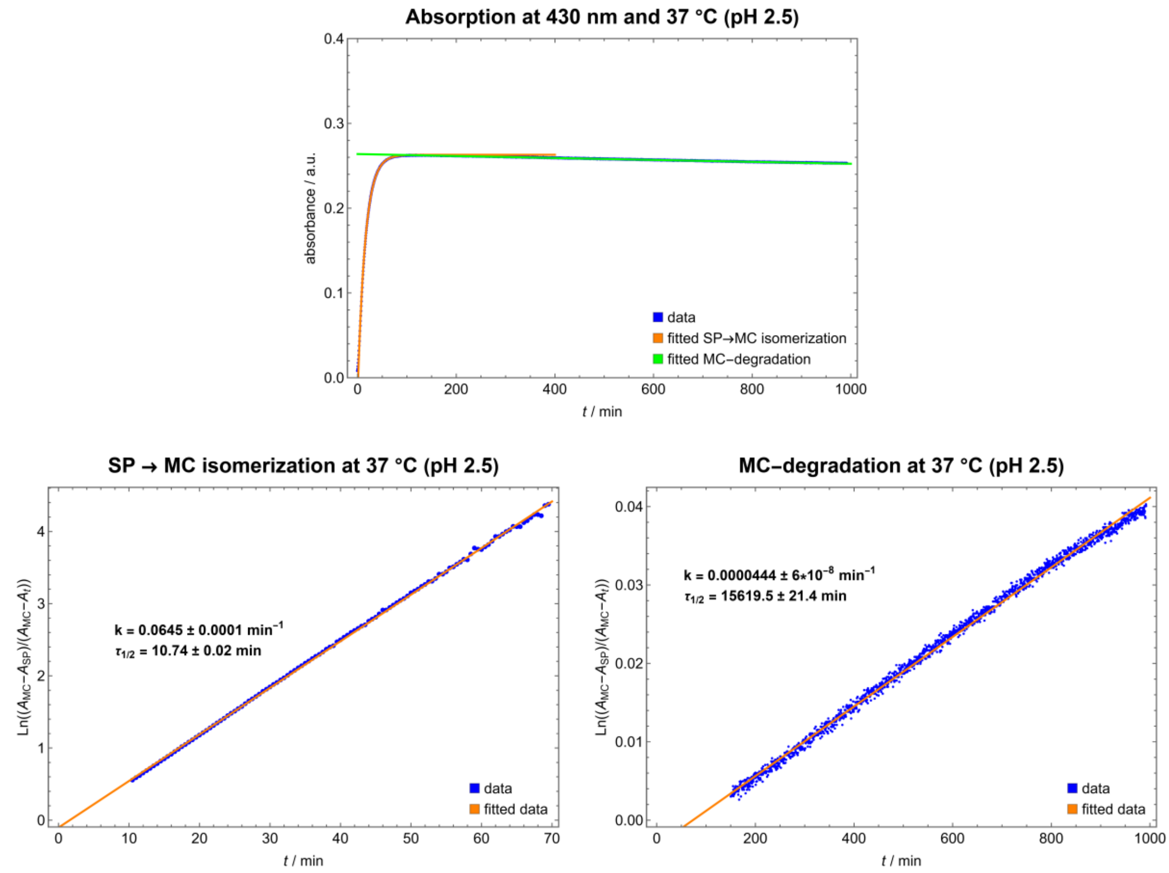

Figure S7. Kinetic measured at 430 nm (top) of SP-to-MC isomerization (rate constant k, lower left) and hydrolysis (rate constant k, lower right) of **P1** (13 µM) at pH 2.5 and 37 °C.

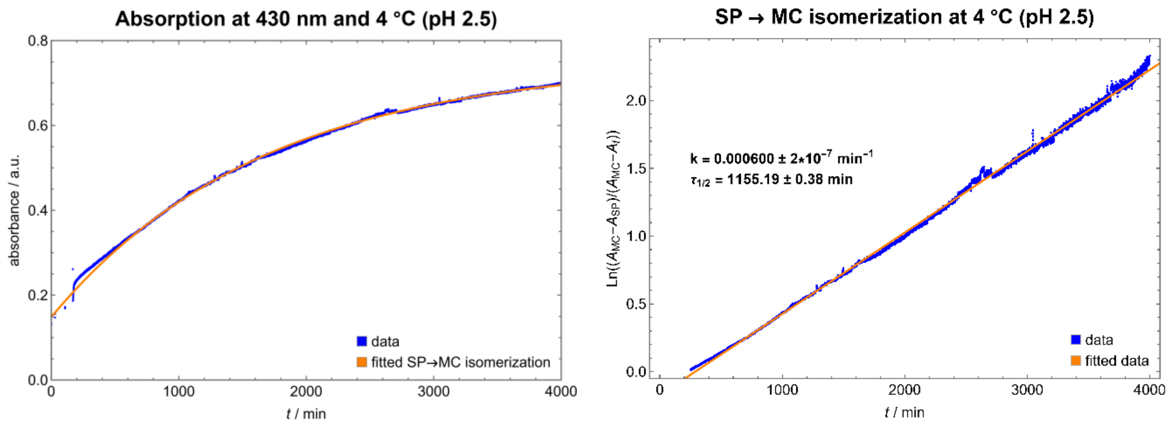

Figure S8. Kinetic measured at 430 nm (left) of SP-to-MC isomerization (rate constant k, right) of **P1** (13 µM) at pH 2.5 and 4 °C.

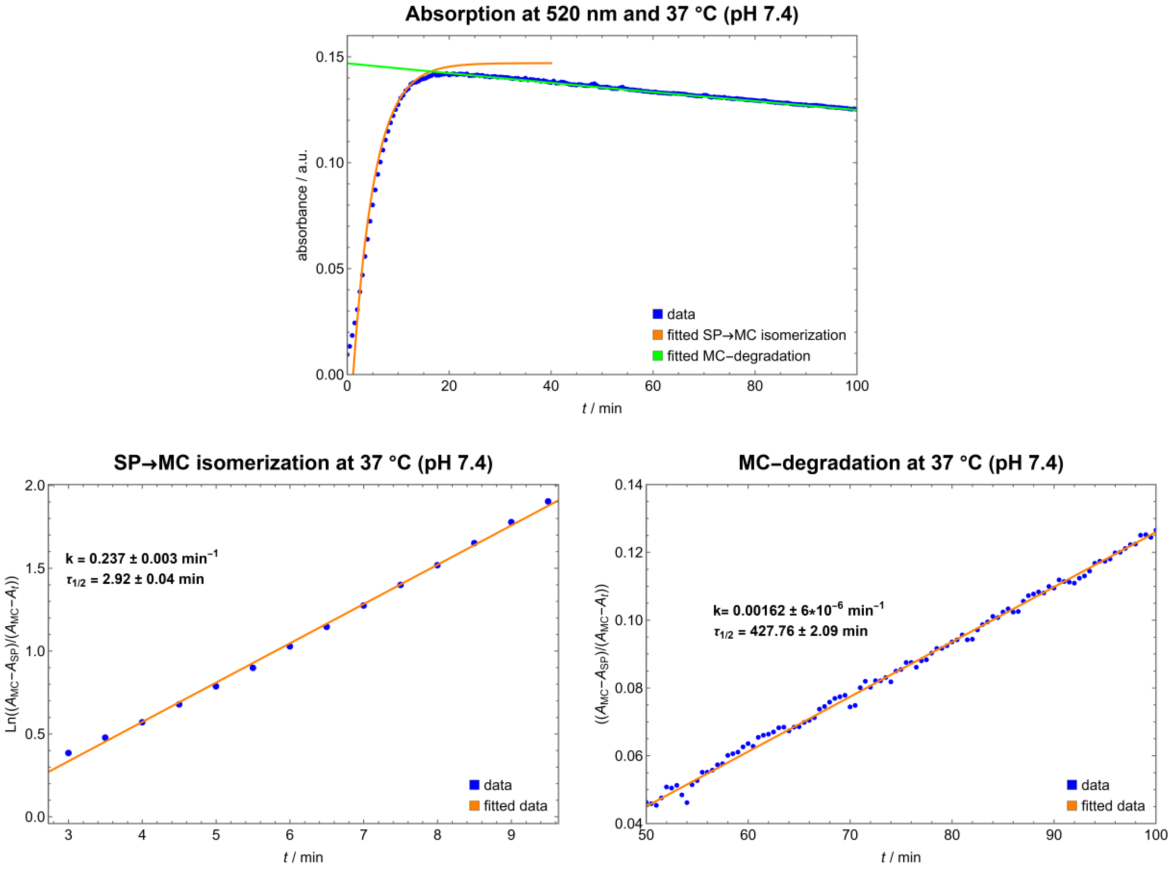

Figure S9. Kinetic measured at 520 nm (top) of SP-to-MC isomerization (rate constant *k*, lower left) and hydrolysis (rate constant *k*, lower right) of **P2** (20 µM) at pH 7.4 and 37 °C.

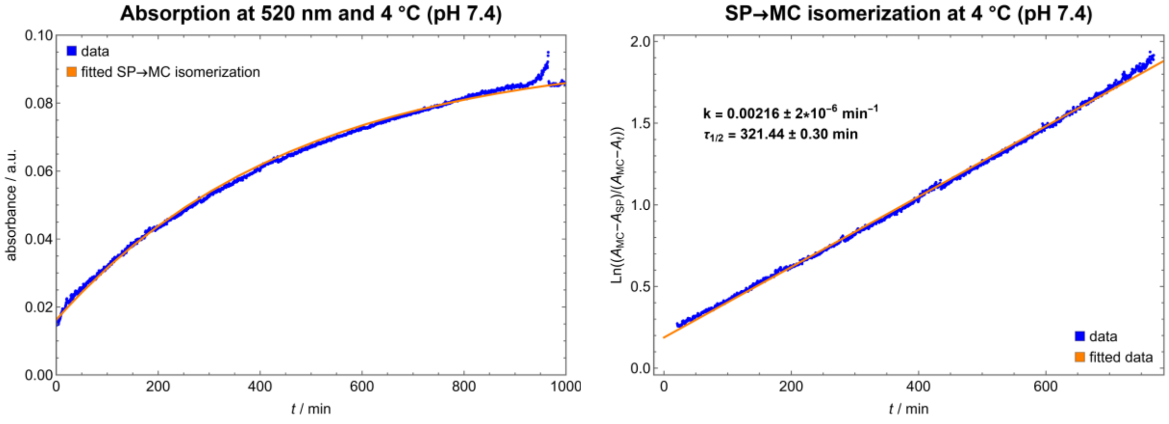

Figure S10. Kinetic measured at 520 nm (left) of SP-to-MC isomerization (rate constant *k*, right) of **P2** (12 µM) at pH 7.4 and 4 °C.

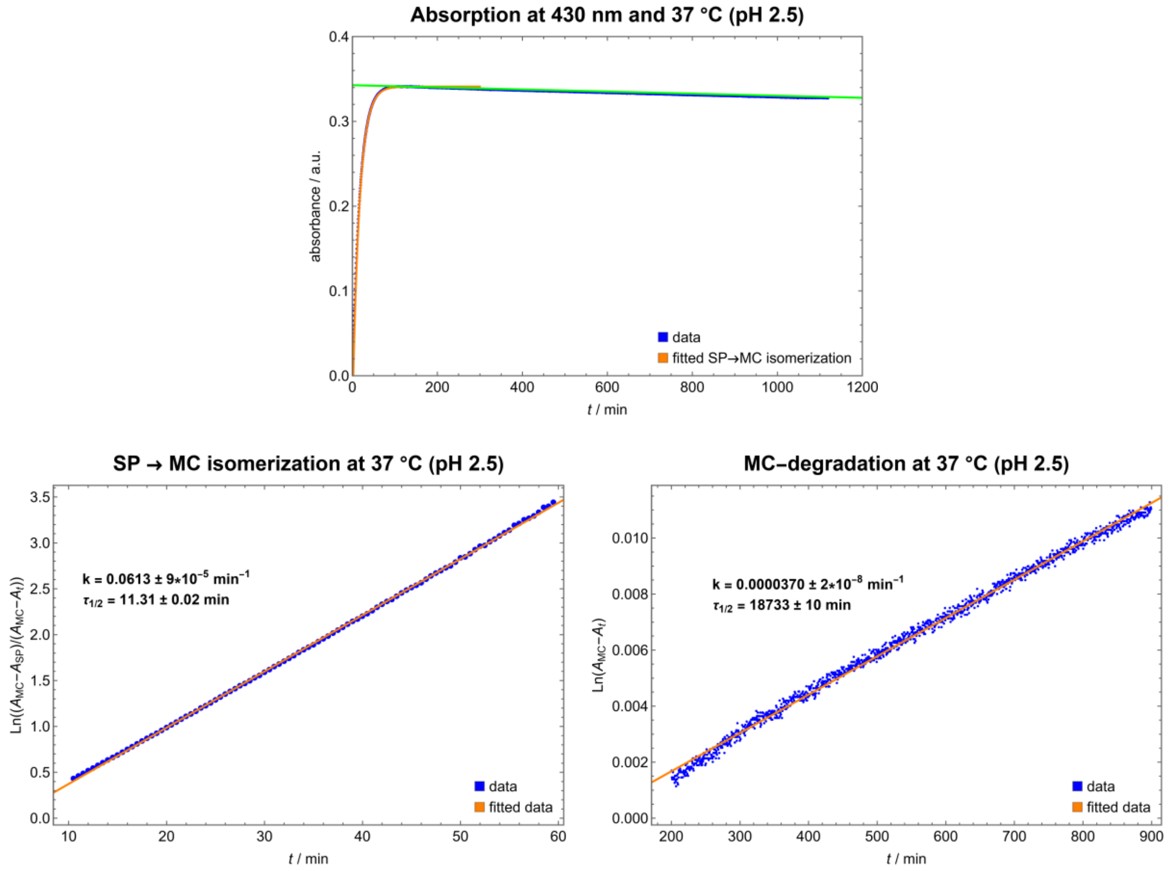

Figure S11. Kinetic measured at 430 nm (top) of SP-to-MC isomerization (rate constant *k*, lower left) and hydrolysis (rate constant *k*, lower right) of **P2** (16 µM) at pH 2.5 and 37 °C.

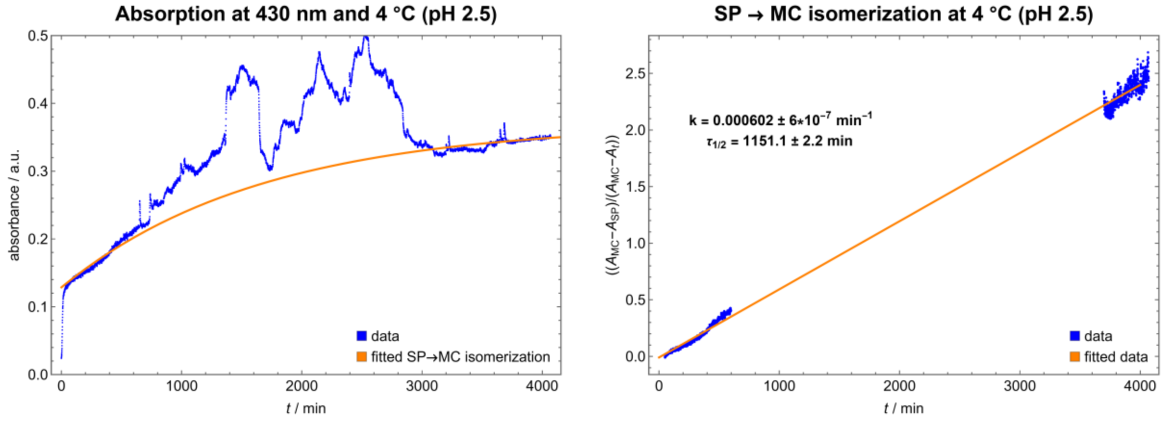

Figure S12. Kinetic measured at 430 nm (left) of SP-to-MC isomerization (rate constant *k*, right) of **P2** (16 µM) at pH 2.5 and 4 °C.

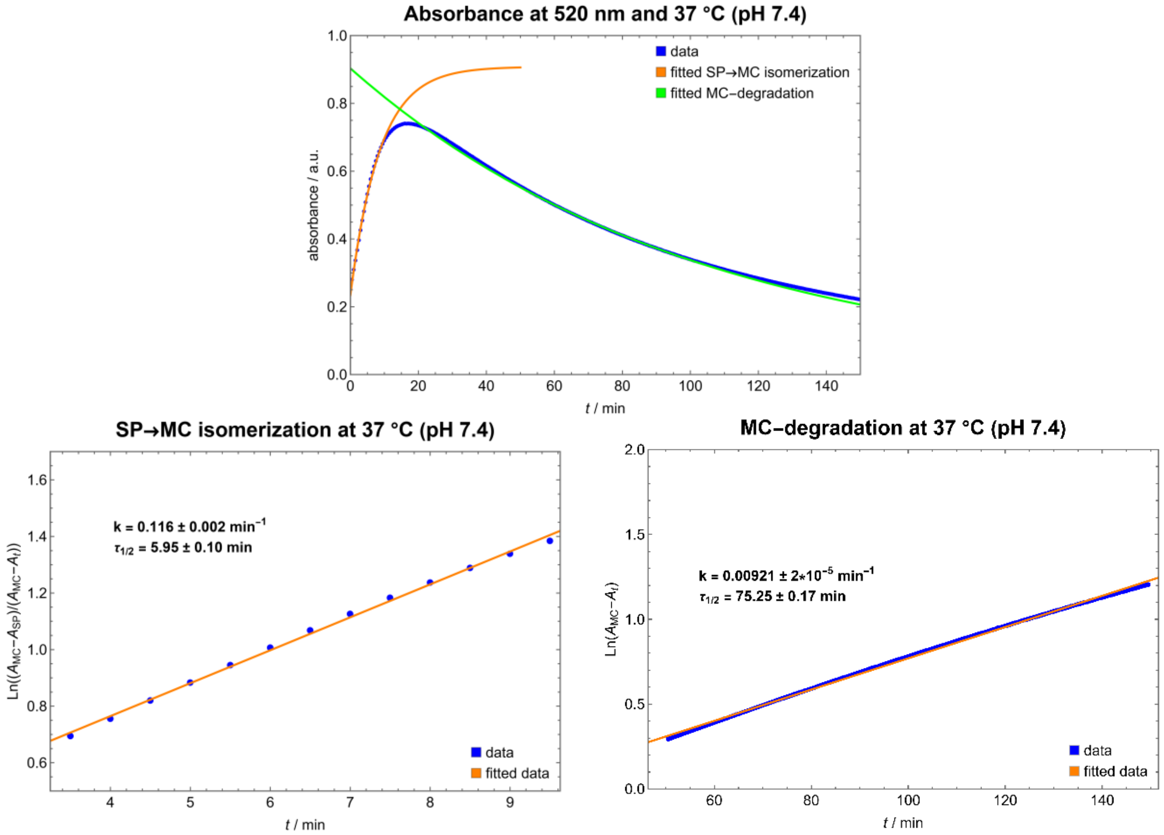

Figure S13. Kinetic measured at 520 nm (top) of SP-to-MC isomerization (rate constant k, lower left) and hydrolysis (rate constant k, lower right) of **P3** (120 µM) at pH 7.4 and 37 °C.

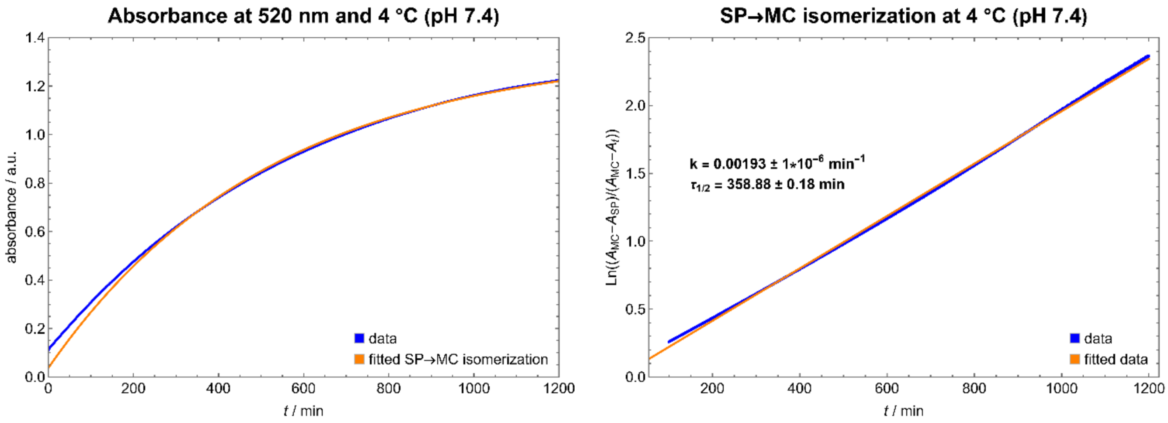

Figure S14. Kinetic measured at 520 nm (left) of SP-to-MC isomerization (rate constant k, right) of **P3** (160 µM) at pH 7.4 and 4 °C.

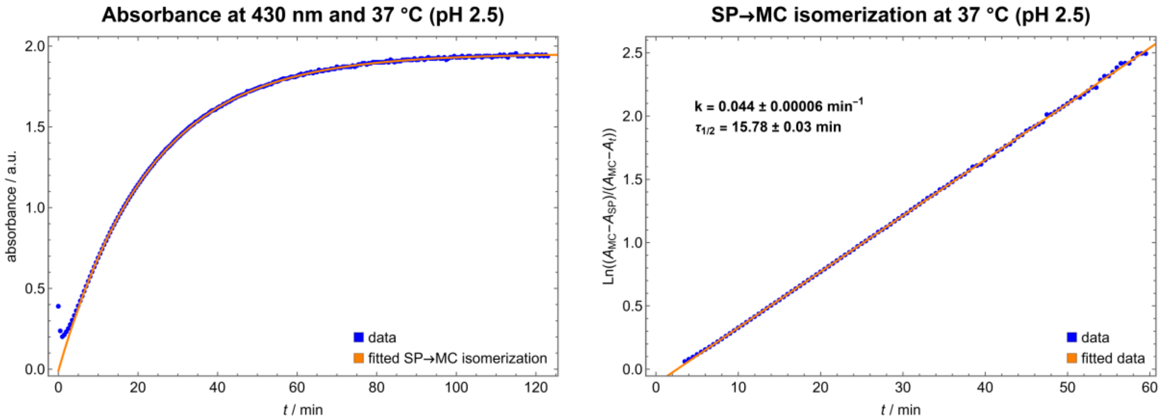

Figure S15. Kinetic measured at 430 nm (left) of SP-to-MC isomerization (rate constant *k*, right) of **P3** (100 µM) at pH 2.5 and 37 °C.

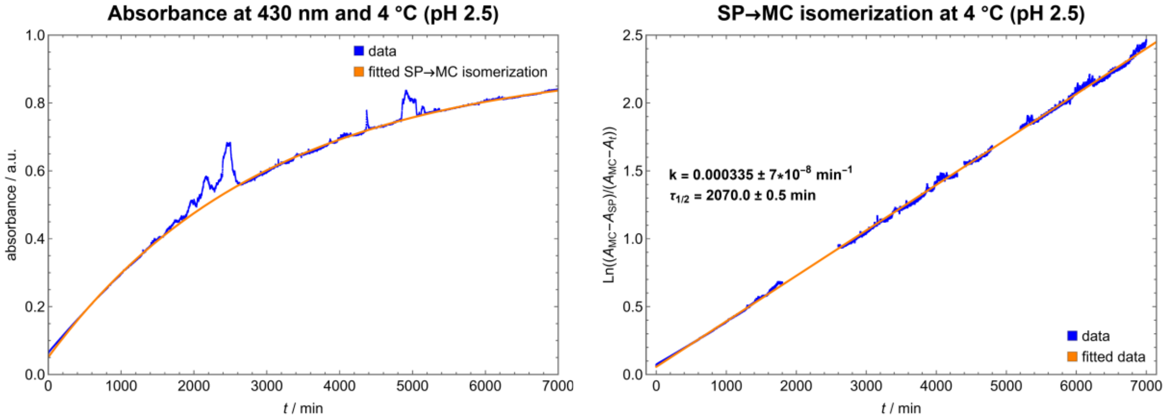

Figure S16. Kinetic measured at 430 nm (left) of SP-to-MC isomerization (rate constant *k*, right) of **P3** (50 µM) at pH 2.5 and 4 °C.

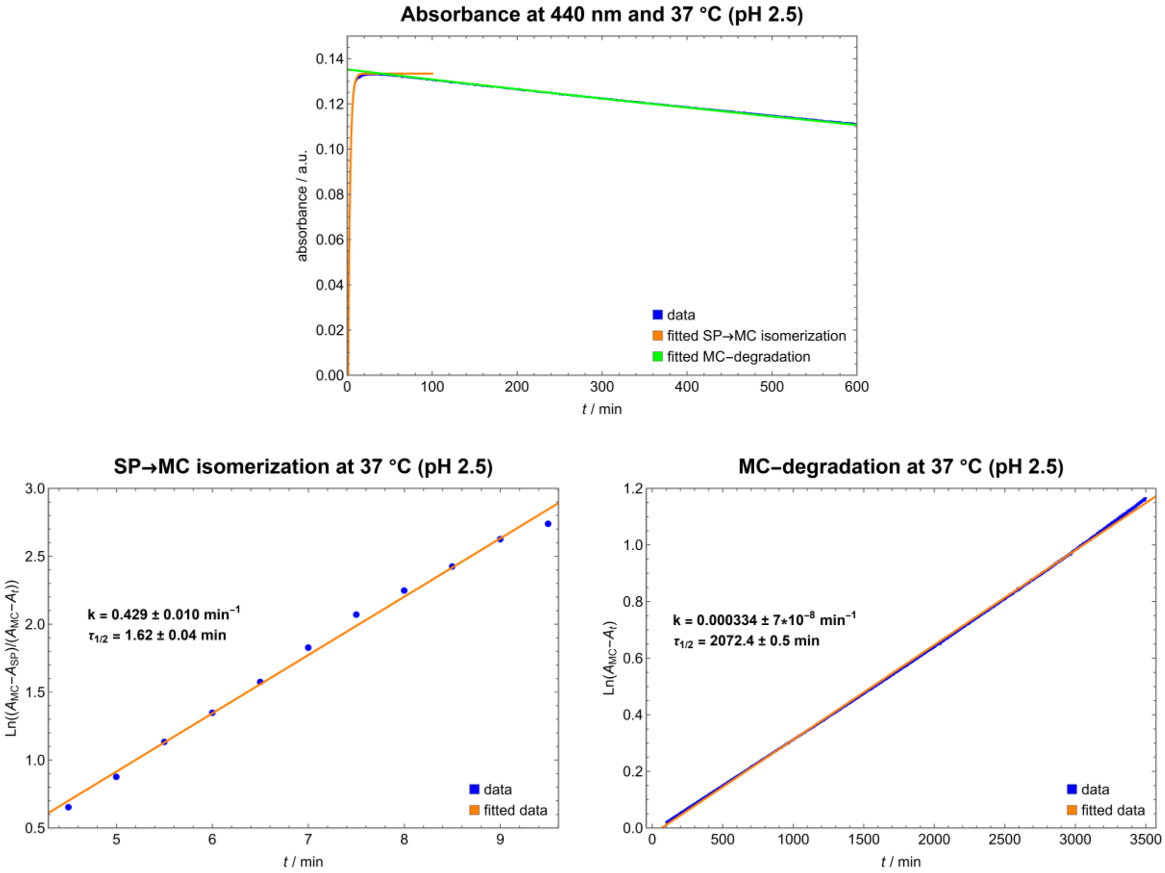

Figure S17. Kinetic measured at 440 nm (top) of SP-to-MC isomerization (rate constant *k*, lower left) and hydrolysis (rate constant *k*, lower right) of **P4** (15 µM) at pH 2.5 and 37 °C.

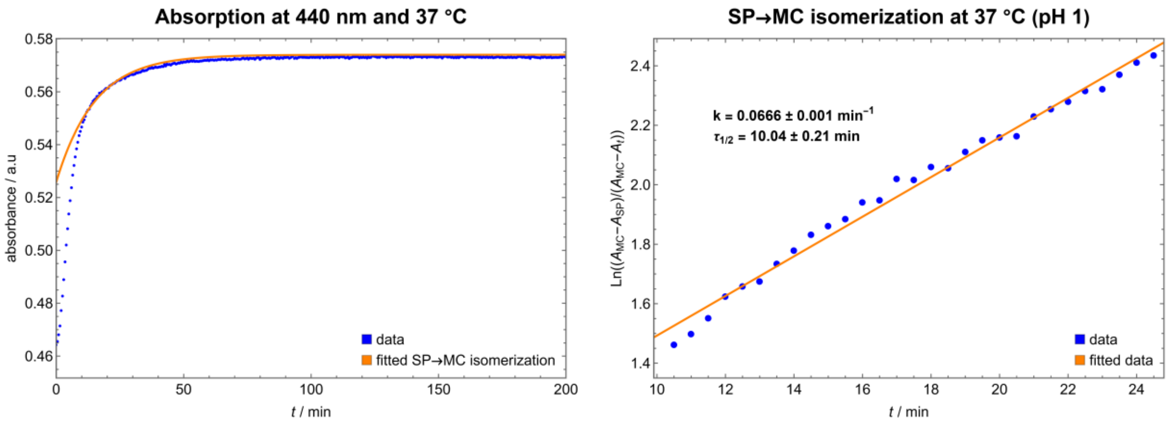

Figure S18. Kinetic measured at 440 nm (left) of SP-to-MC isomerization (rate constant *k*, right) of **P4** (44 µM) at pH 1 and 37 °C.

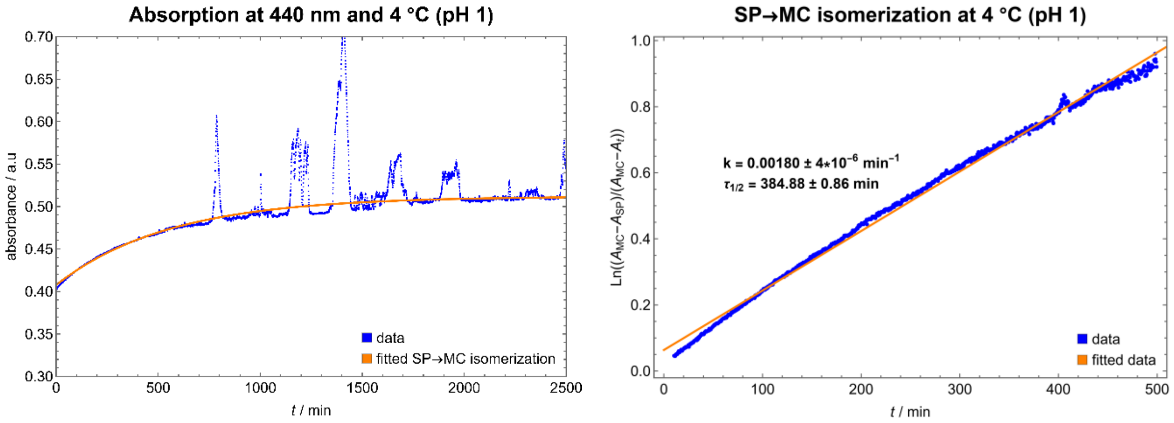

Figure S19. Kinetic measured at 440 nm (left) of SP-to-MC isomerization (rate constant *k*, right) of **P4** (39 µM) at pH 1 and 4 °C.

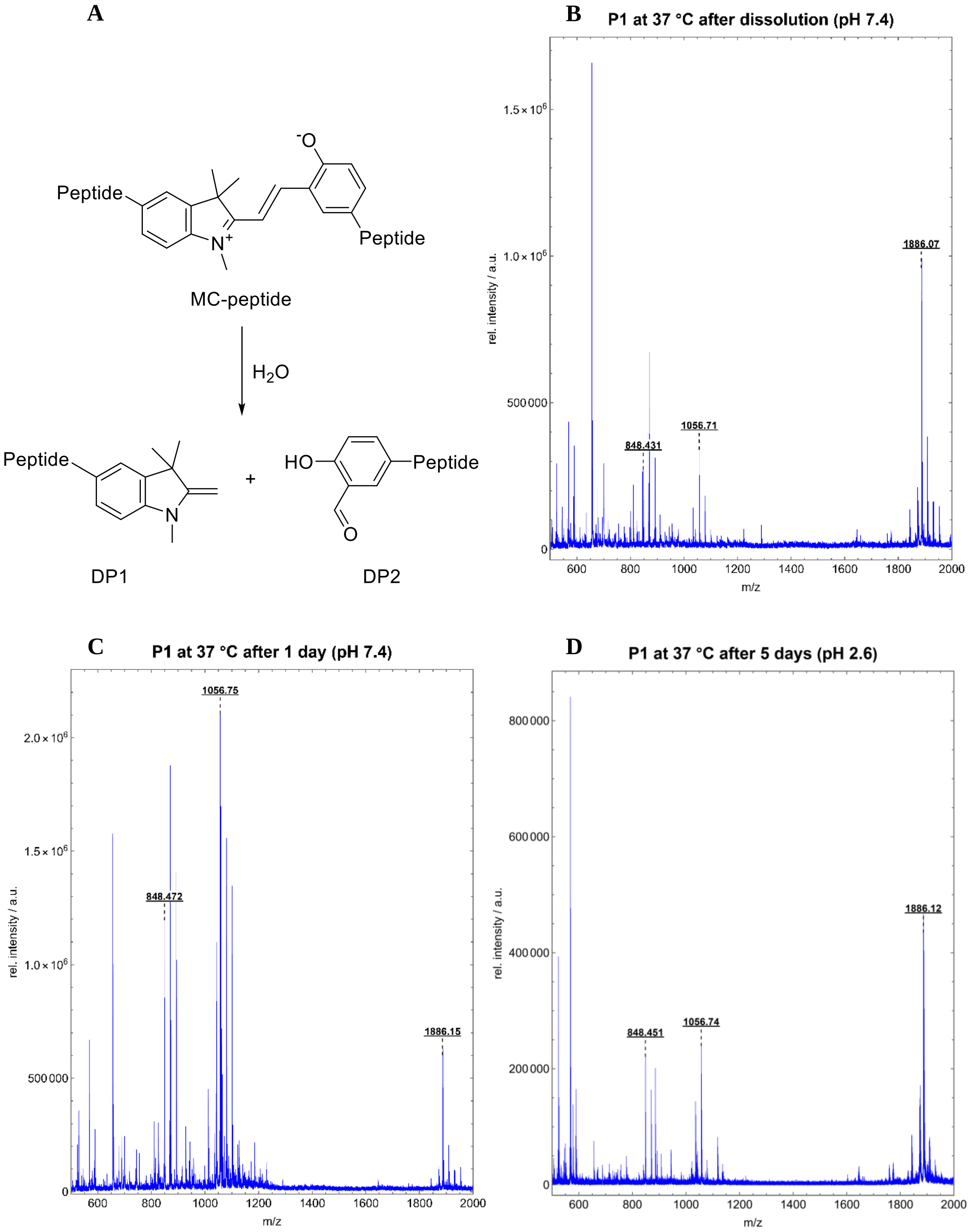

Figure S20. (A) Products of the hydrolysis reaction. (C – D) MALDI-measurements of **P1** and hydrolysis products at 37 °C after dissolution at pH 7.4 (B), after 1 day at pH 7.4 (C), and after 5 days at pH 2.6 (D). Calculated mass of **P1**+H^+: 1886.0435, calculated mass for DP1+H^+: 1056.6600, calculated mass for DP2+H^+: 848.3997.

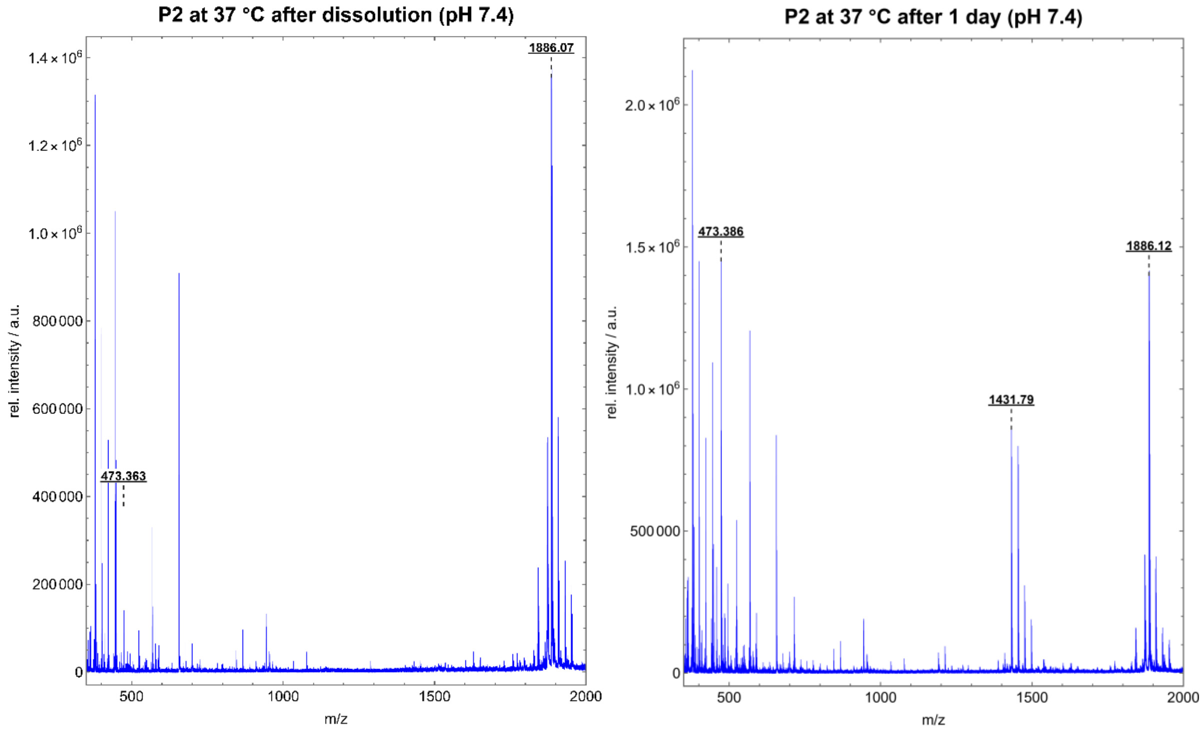

Figure S21. MALDI-measurements of **P2** and hydrolysis products at 37 °C after dissolution at pH 7.4 (left) and after 1 day at pH 7.4 (right). Calculated mass of **P2**+H$^+$: 1886.0435, calculated mass for DP1+H$^+$: 473.3347, calculated mass for DP2+H$^+$: 1431.7267.

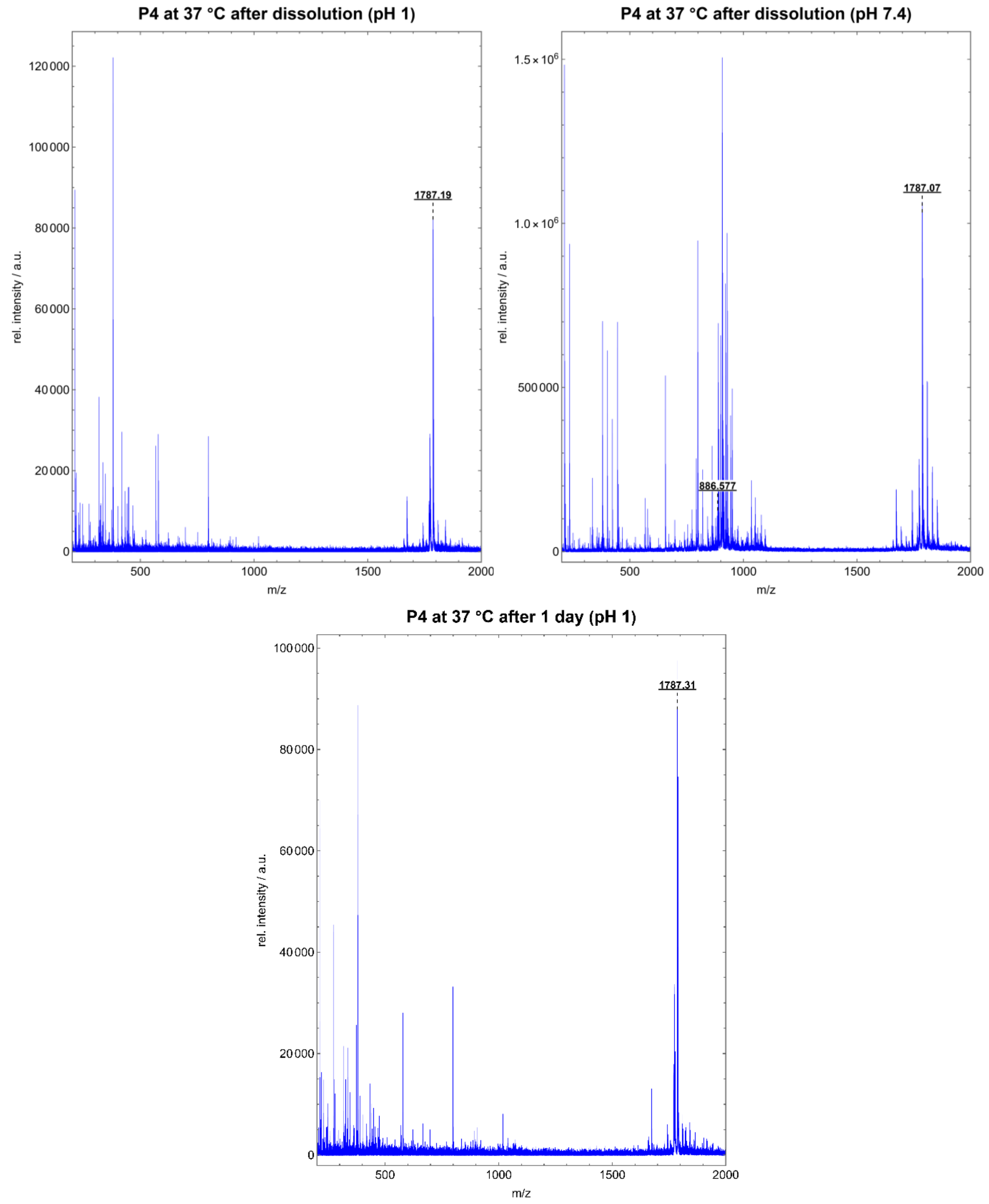

Figure S22. MALDI-measurements of **P4** and hydrolysis products at 37 °C after dissolution at pH 7.4 (top, left), after dissolution at pH 1 (top, right), and after 1 day at pH 1 (bottom). Calculated mass of **P4**+H^+: 1786.9571, calculated mass for DP1+H^+: 886.4934, calculated mass for DP2+H^+: 919.4996.

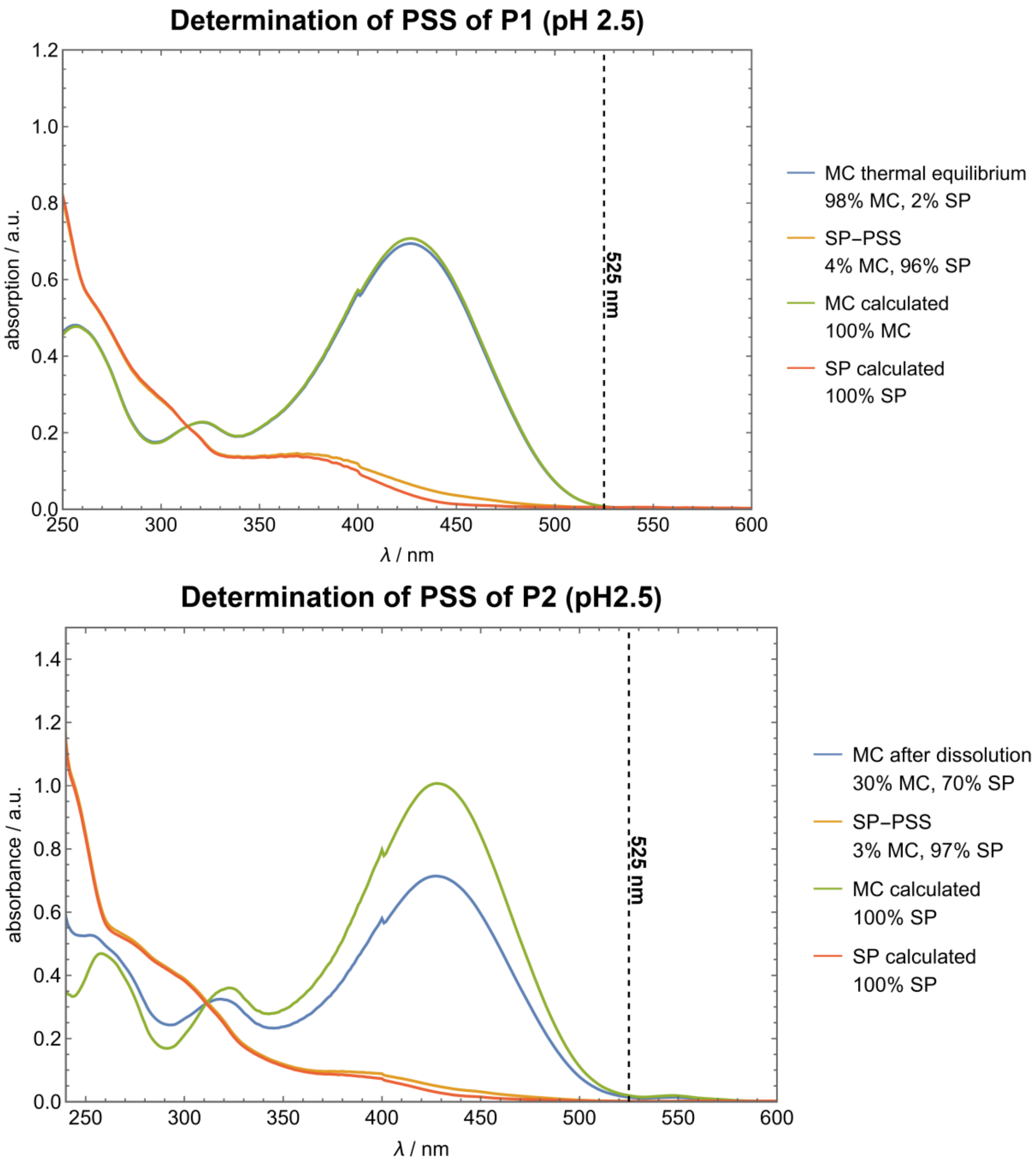

Figure S23. UV/Vis-spectra of **P1** (top, 35 µM) and **P2** (bottom, 56 µM) at pH 2.5. Spectra were measured at thermal equilibrium (P1), after dissolution (P2) and in the SP-PSS (after irradiation with green light for 10 min). SP:MC ratio was analyzed with HPLC and the spectra of the pure MC- and SP-form were calculated using Mathematic 13.3.1.0.

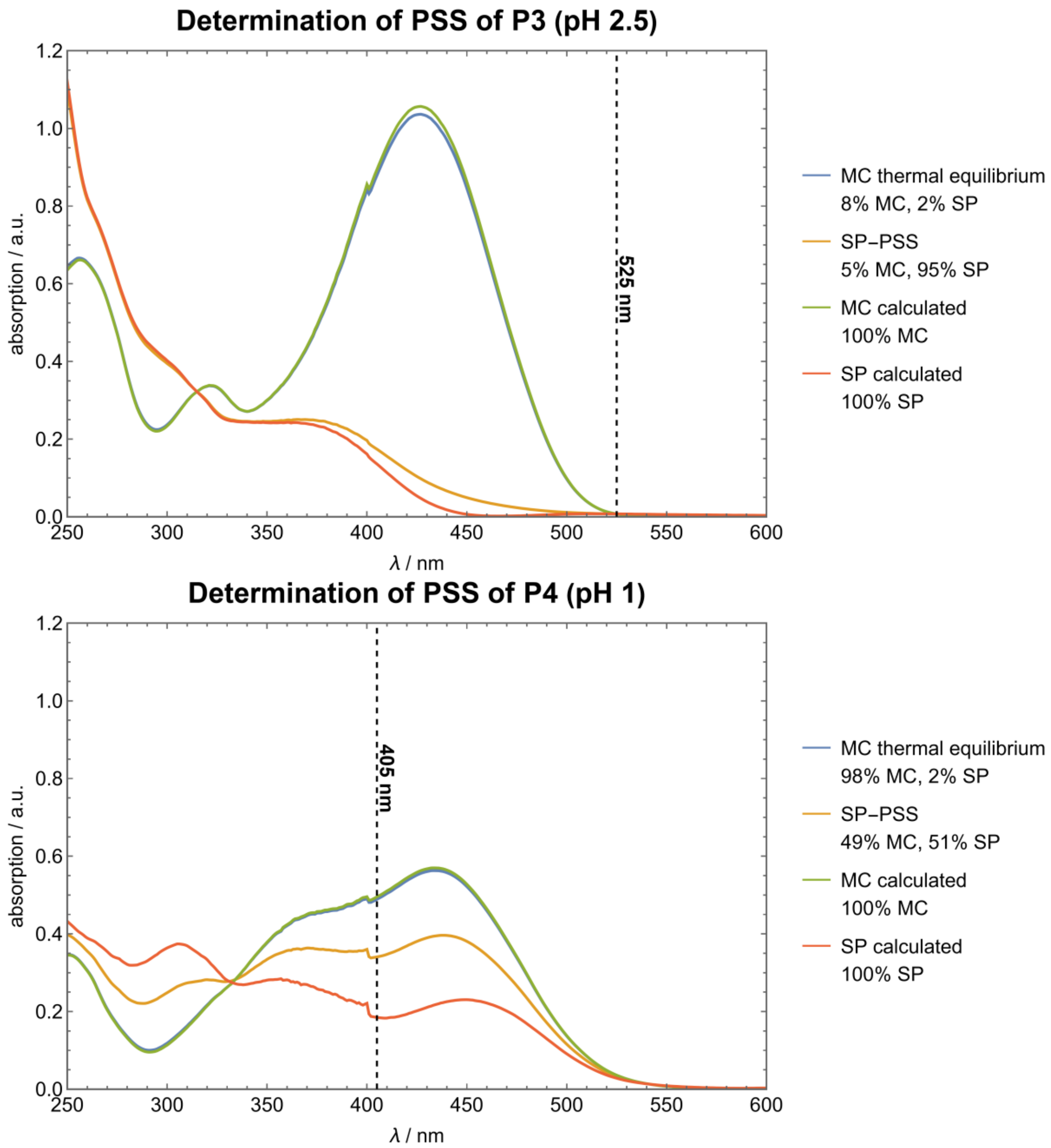

Figure S24. UV/Vis-spectra of **P3** at pH 2.5 (top, 55 µM) and **P4** at pH 1 (bottom, 44 µM). Spectra were measured at thermal equilibrium and in the SP-PSS (for **P3** after irradiation with green light for 10 min, for **P4** after irradiation with 405 nm for 10 min). SP:MC ratio was analyzed with HPLC and the spectra of the pure MC- and SP-form were calculated using Mathematic 13.3.1.0.

5. NMR data of compounds and peptides and LC-MS of peptides

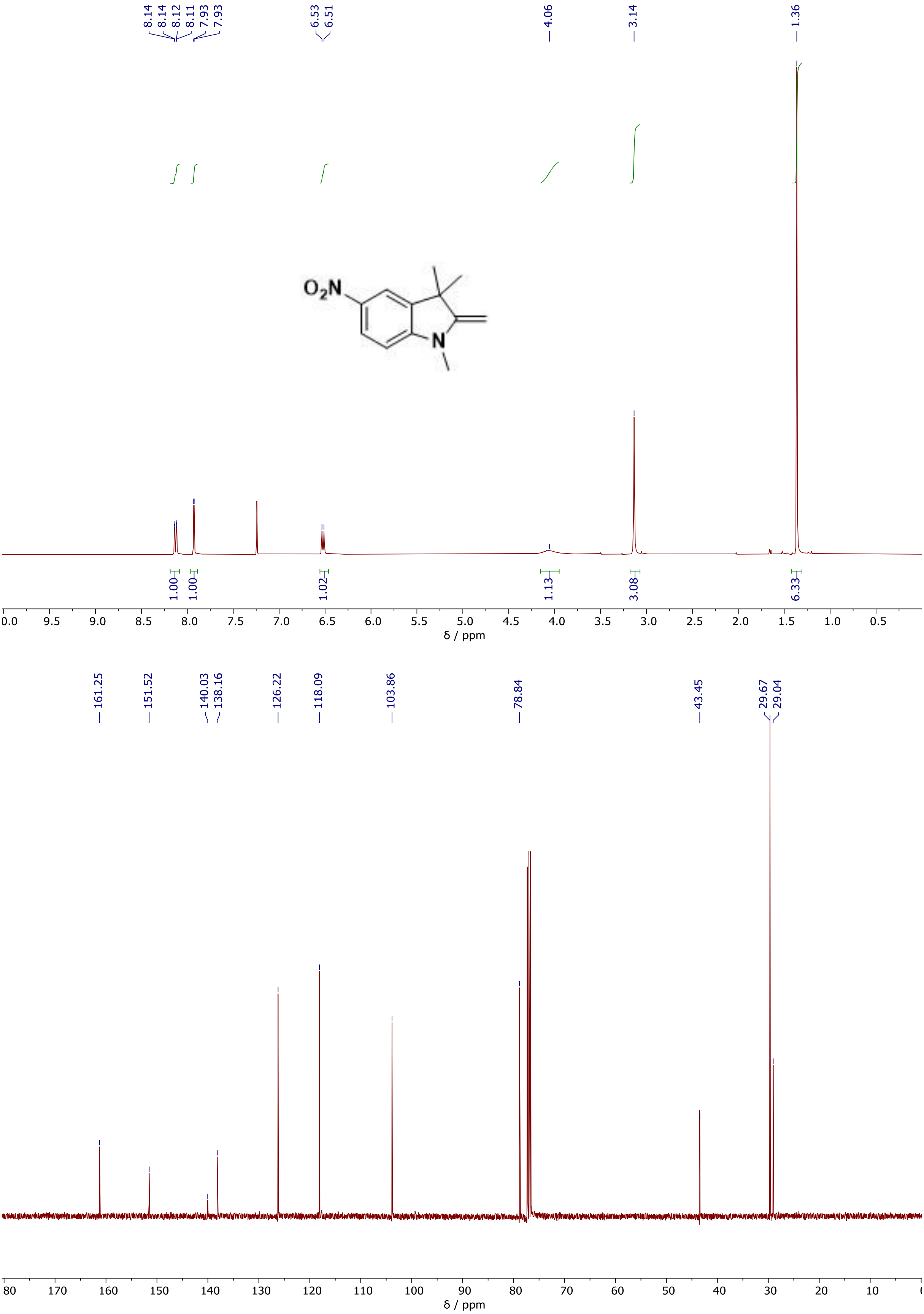

Figure S25. ^{1}H-NMR (top, 500 MHz) and ^{13}C-NMR (bottom, 125 MHz) in $CDCl_3$ of **3-I**.

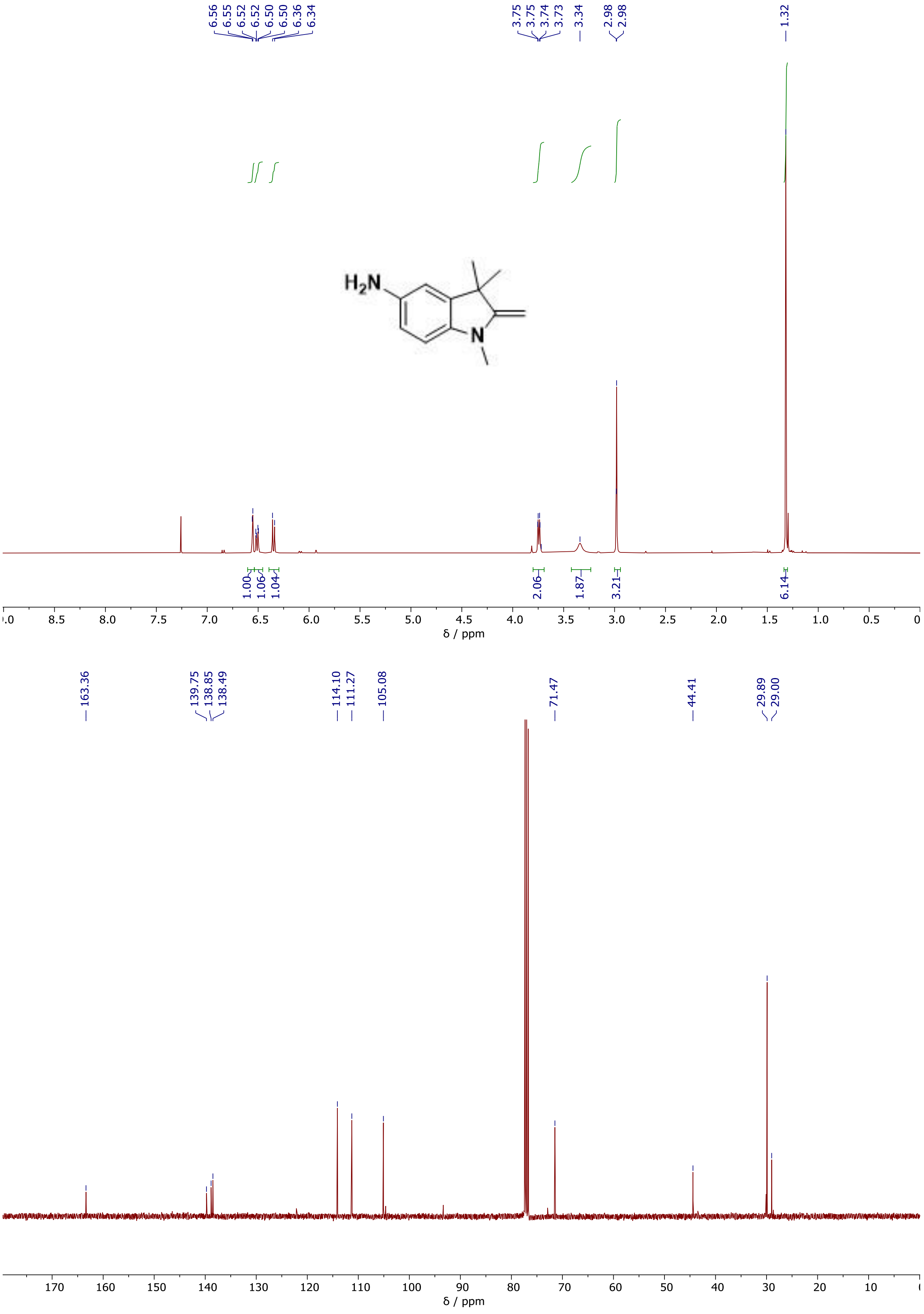

Figure S26. ^{1}H-NMR (top, 500 MHz) and ^{13}C-NMR (bottom, 125 MHz) in $CDCl_3$ of **3-II**.

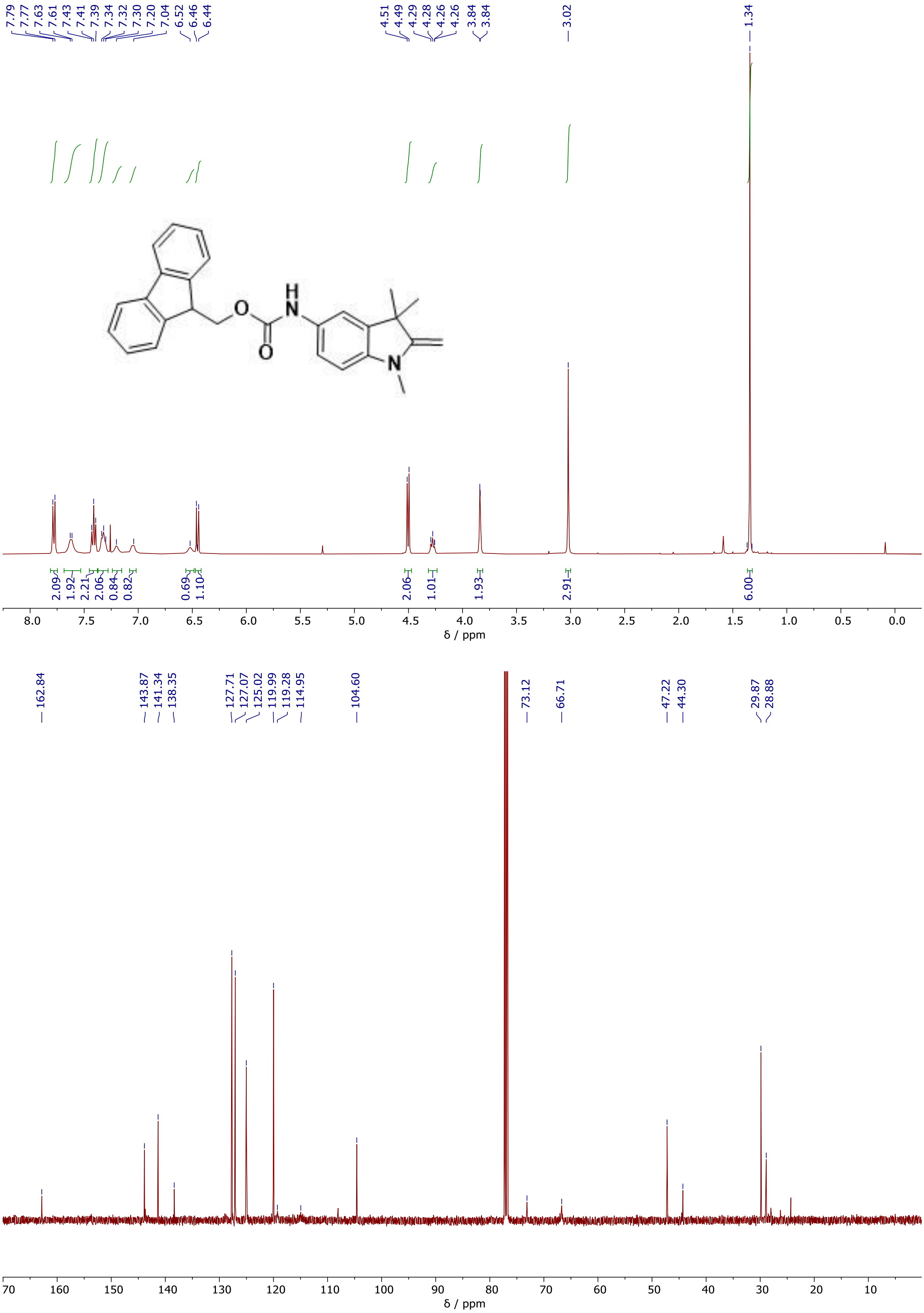

Figure S27. ^{1}H-NMR (top, 500 MHz) and ^{13}C-NMR (bottom, 125 MHz) in $CDCl_3$ of **4a**.

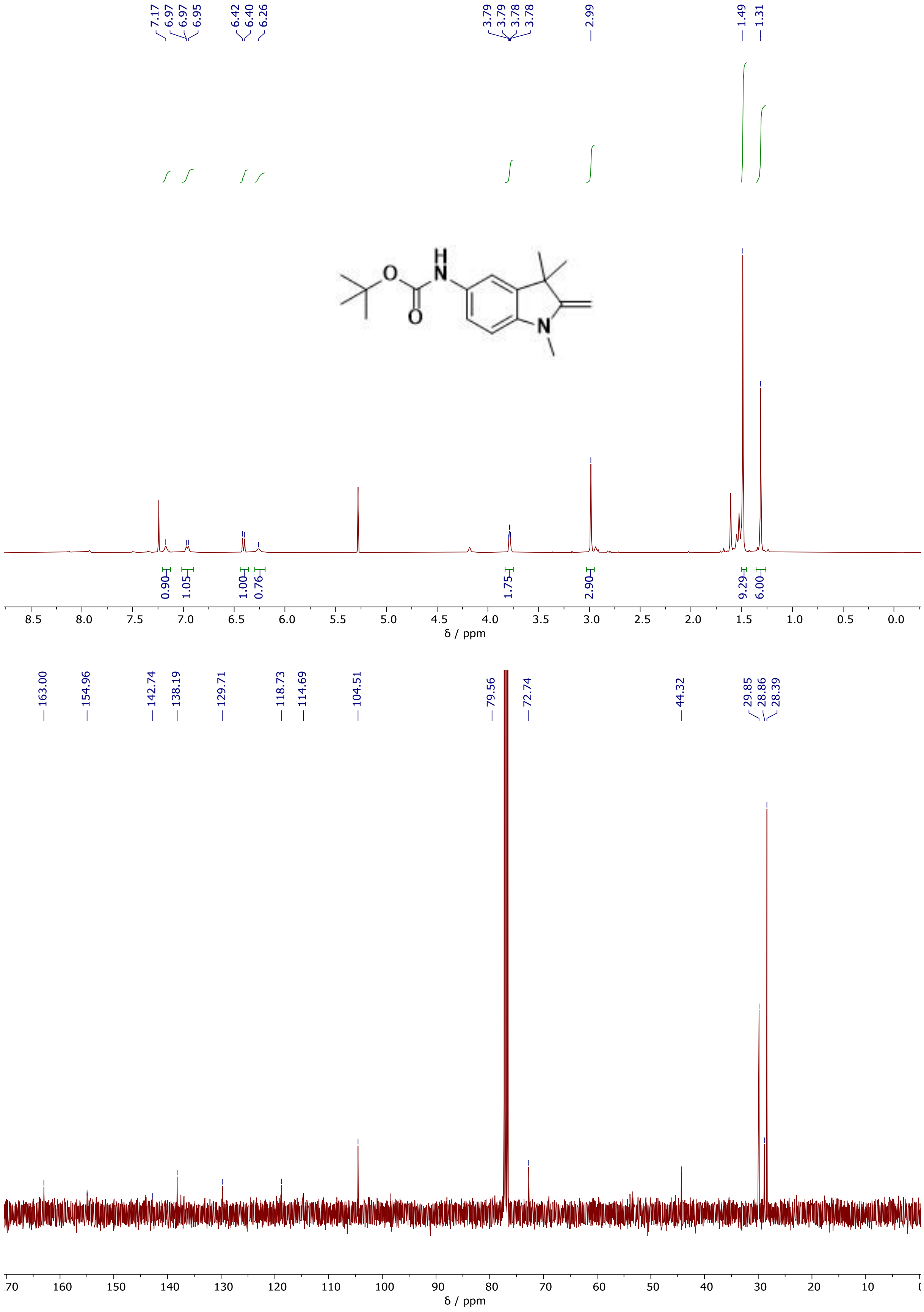

Figure S28. ^{1}H-NMR (top, 400 MHz) and ^{13}C-NMR (bottom, 100 MHz) in $CDCl_3$ of **4b**.

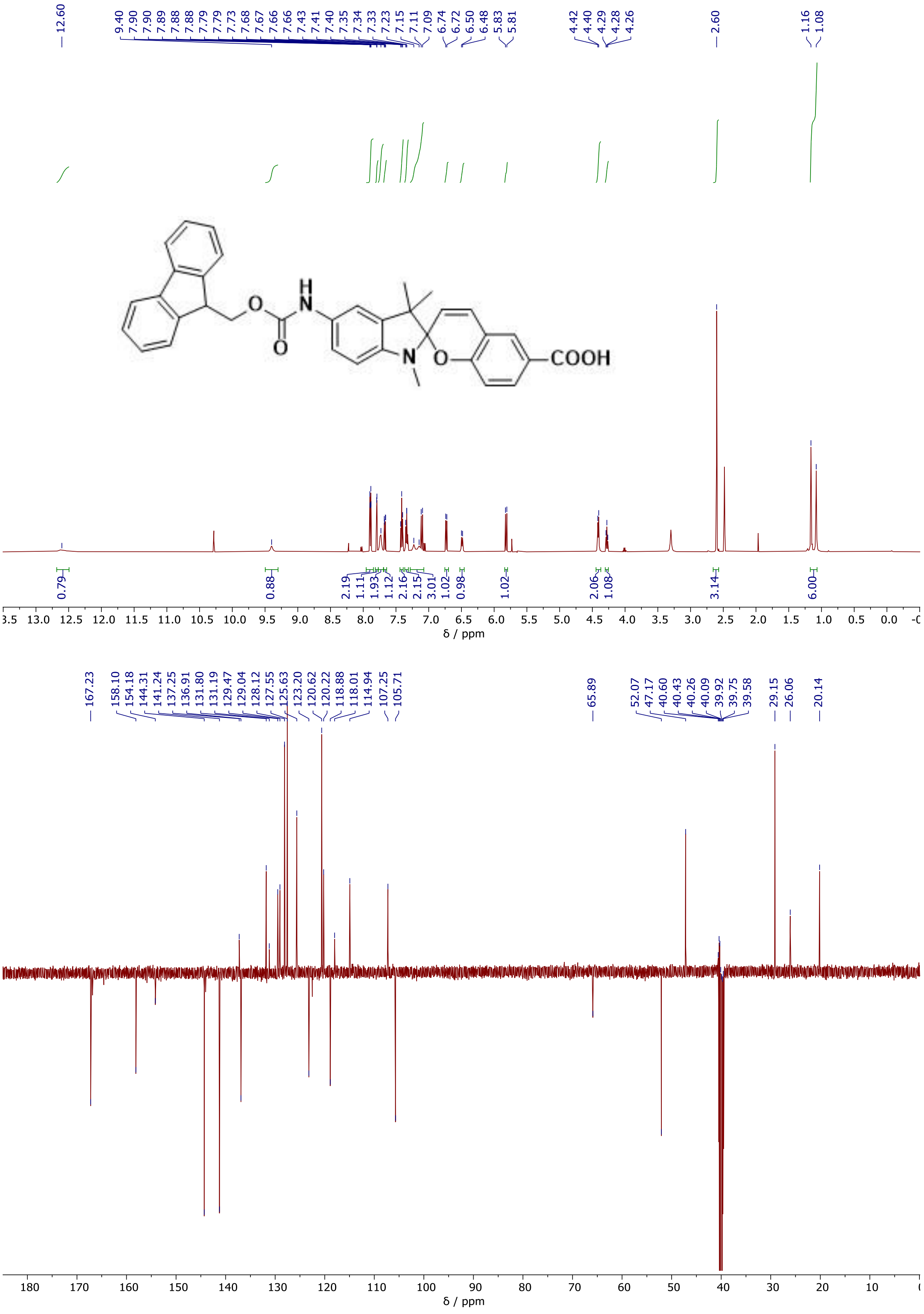

Figure S29. ^{1}H-NMR (top, 500 MHz) and ^{13}C-NMR (bottom, 125 MHz) in DMSO-d_6 of **1a**.

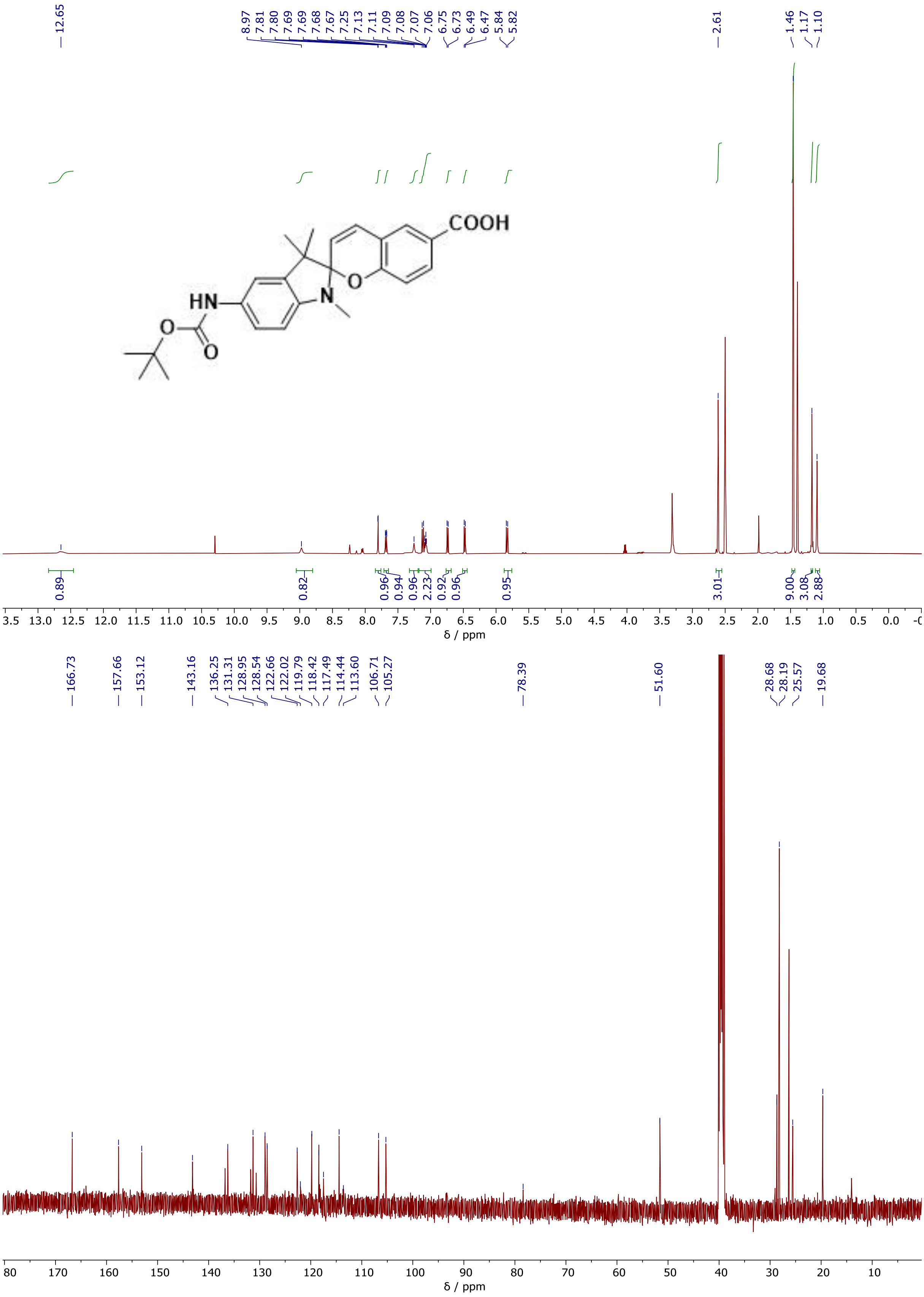

Figure S30. ^{1}H-NMR (top, 500 MHz) and ^{13}C-NMR (bottom, 125 MHz) in DMSO-d_6 of **1b**.

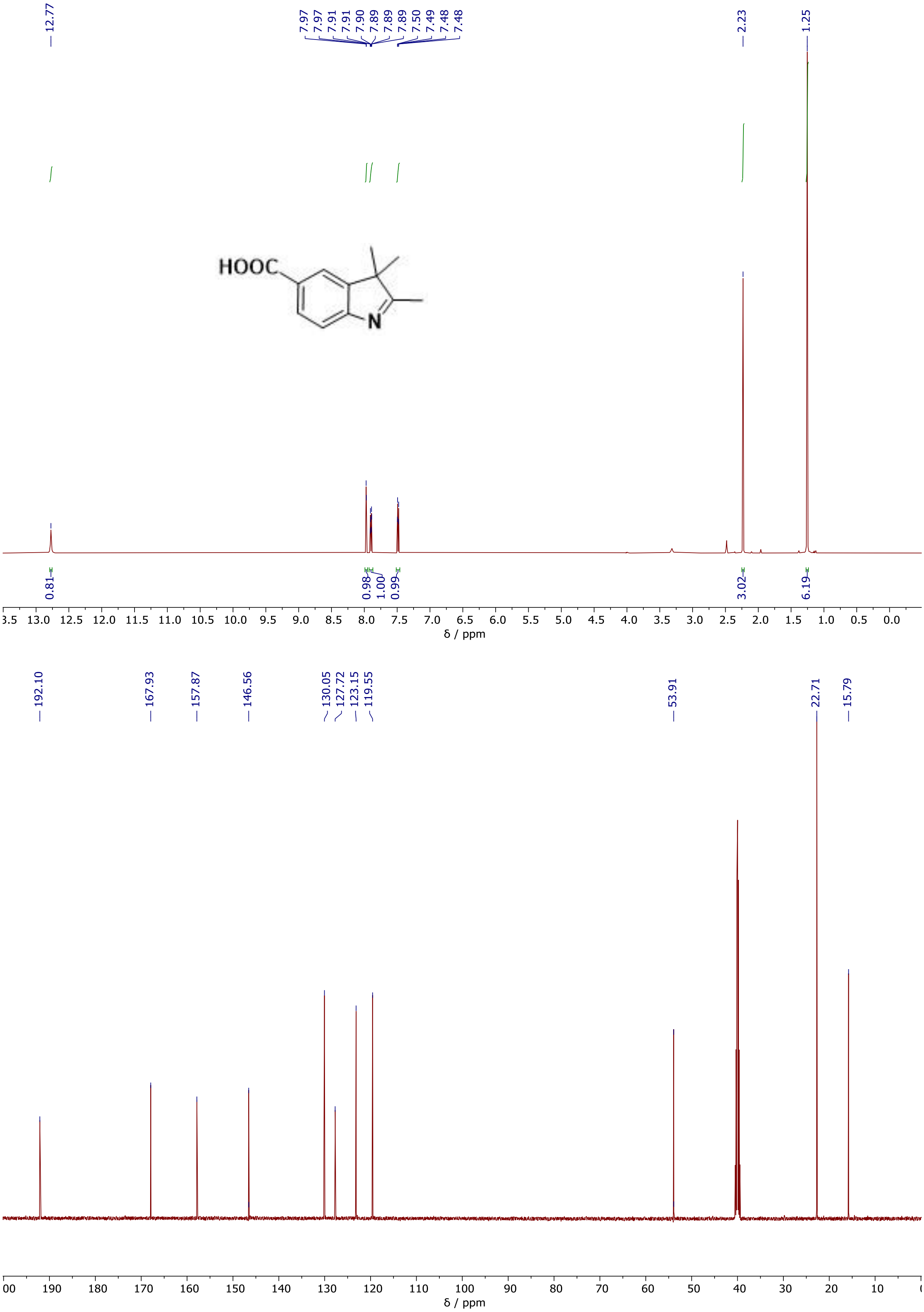

Figure S31. ^{1}H-NMR (top, 500 MHz) and ^{13}C-NMR (bottom, 125 MHz) in $CDCl_3$ of **6-I**.

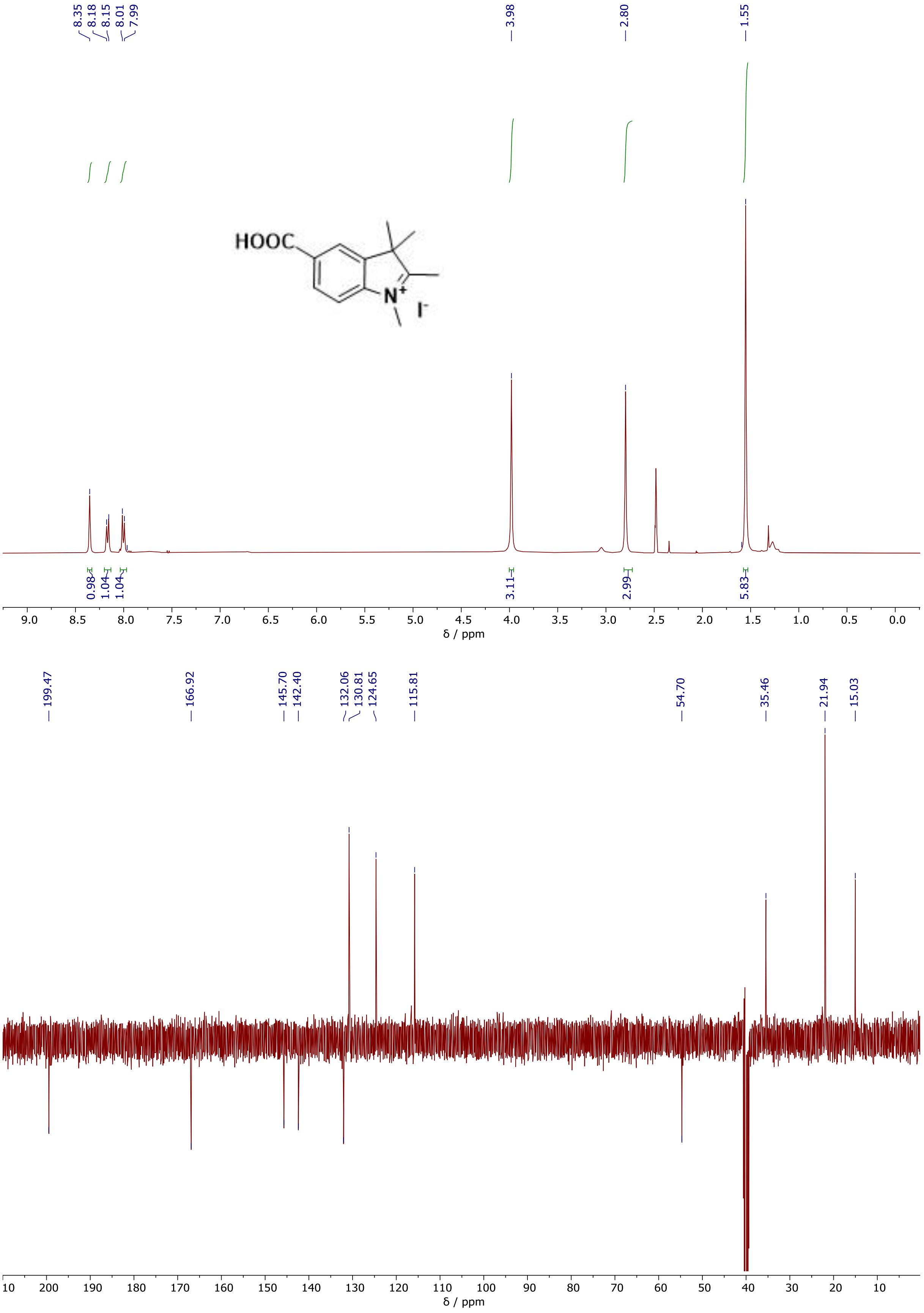

Figure S32. ^{1}H-NMR (top, 400 MHz) and ^{13}C-NMR (bottom, 100 MHz) in DMSO-d_6 of **7**.

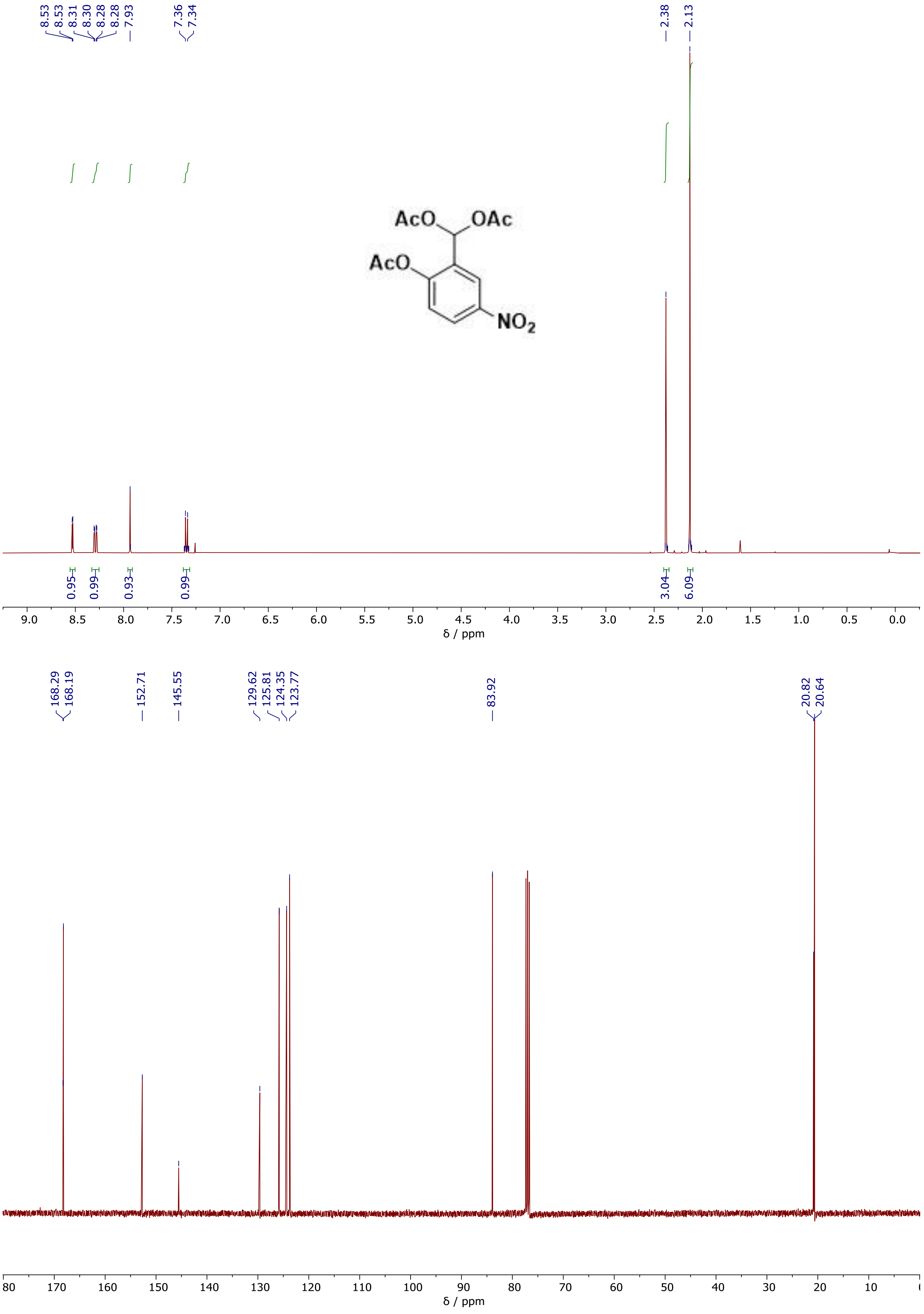

Figure S33. ^{1}H-NMR (top, 400 MHz) and ^{13}C-NMR (bottom, 100 MHz) in $CDCl_3$ of **8-I**.

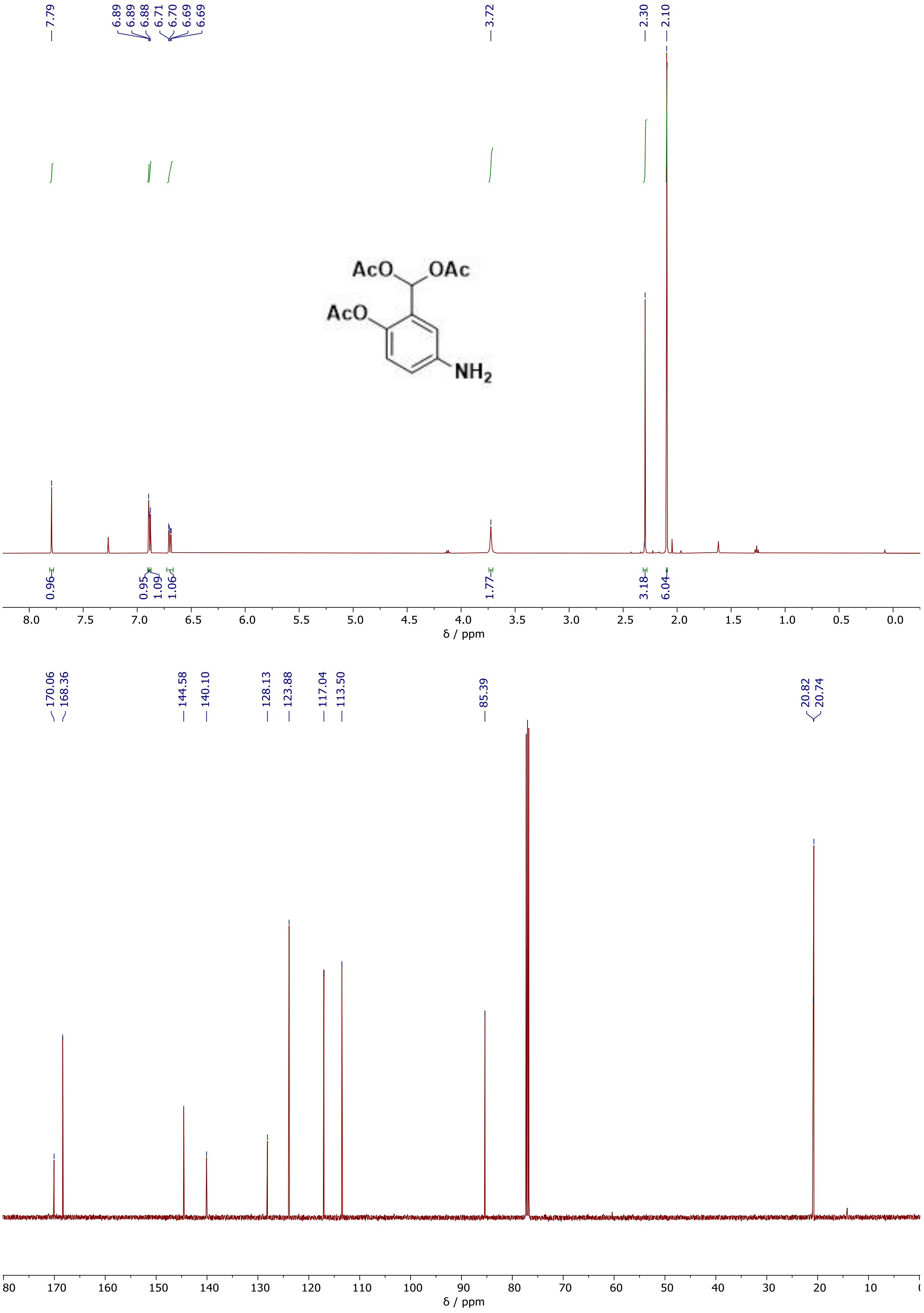

Figure S34. ^{1}H-NMR (top, 500 MHz) and ^{13}C-NMR (bottom, 125 MHz) in $CDCl_3$ of **8-II**.

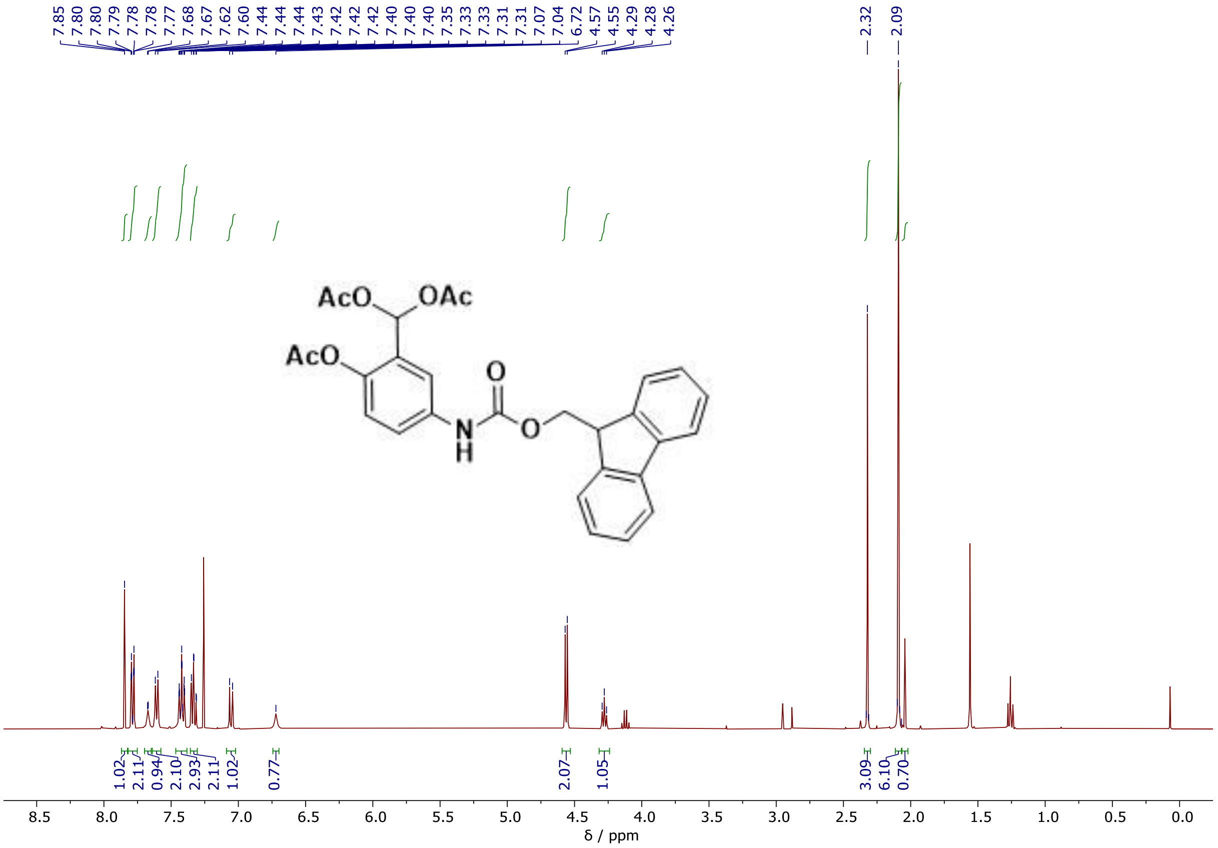

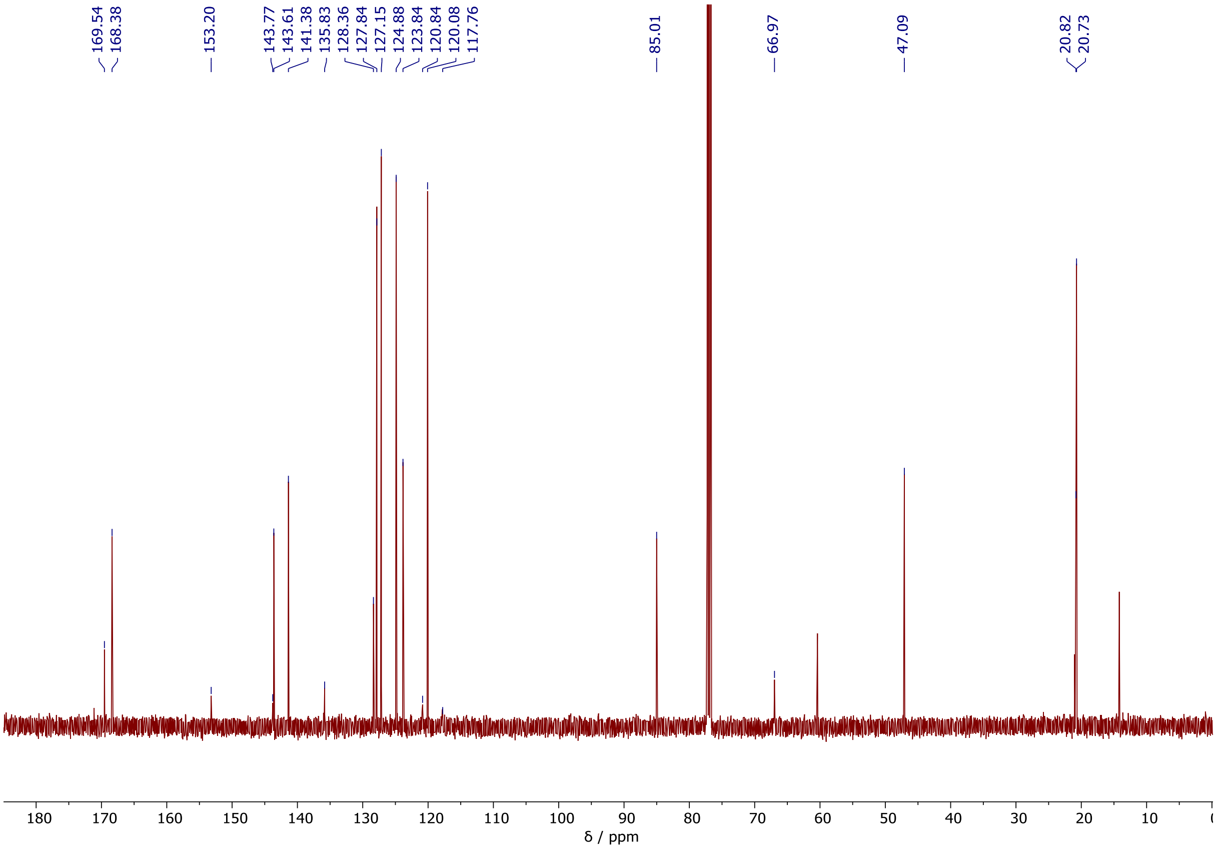

Figure S35. ^{1}H-NMR (top, 500 MHz) and ^{13}C-NMR (bottom, 125 MHz) in $CDCl_3$ of **9a**.

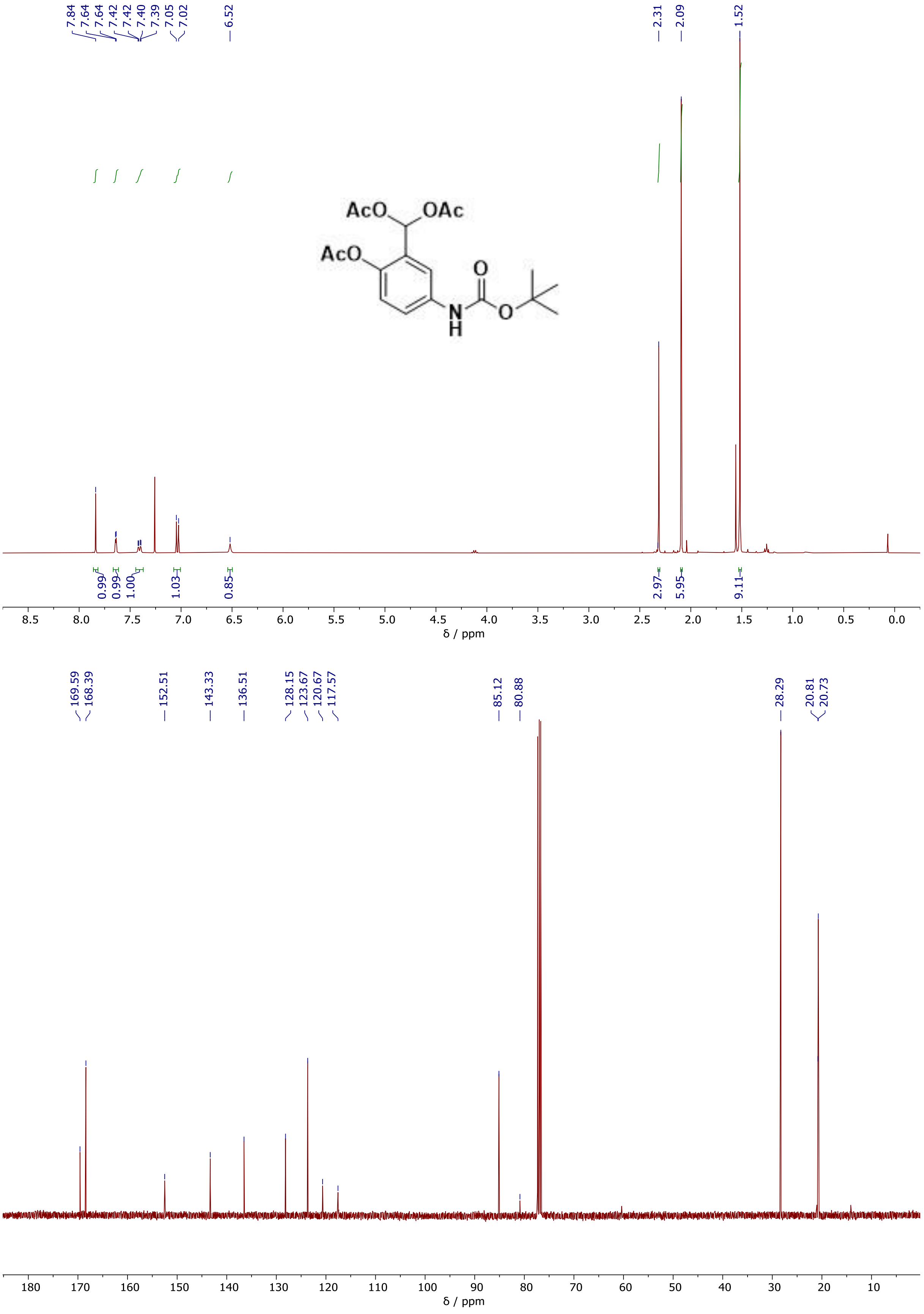

Figure S36. ^{1}H-NMR (top, 400 MHz) and ^{13}C-NMR (bottom, 100 MHz) in $CDCl_3$ of **9b**.

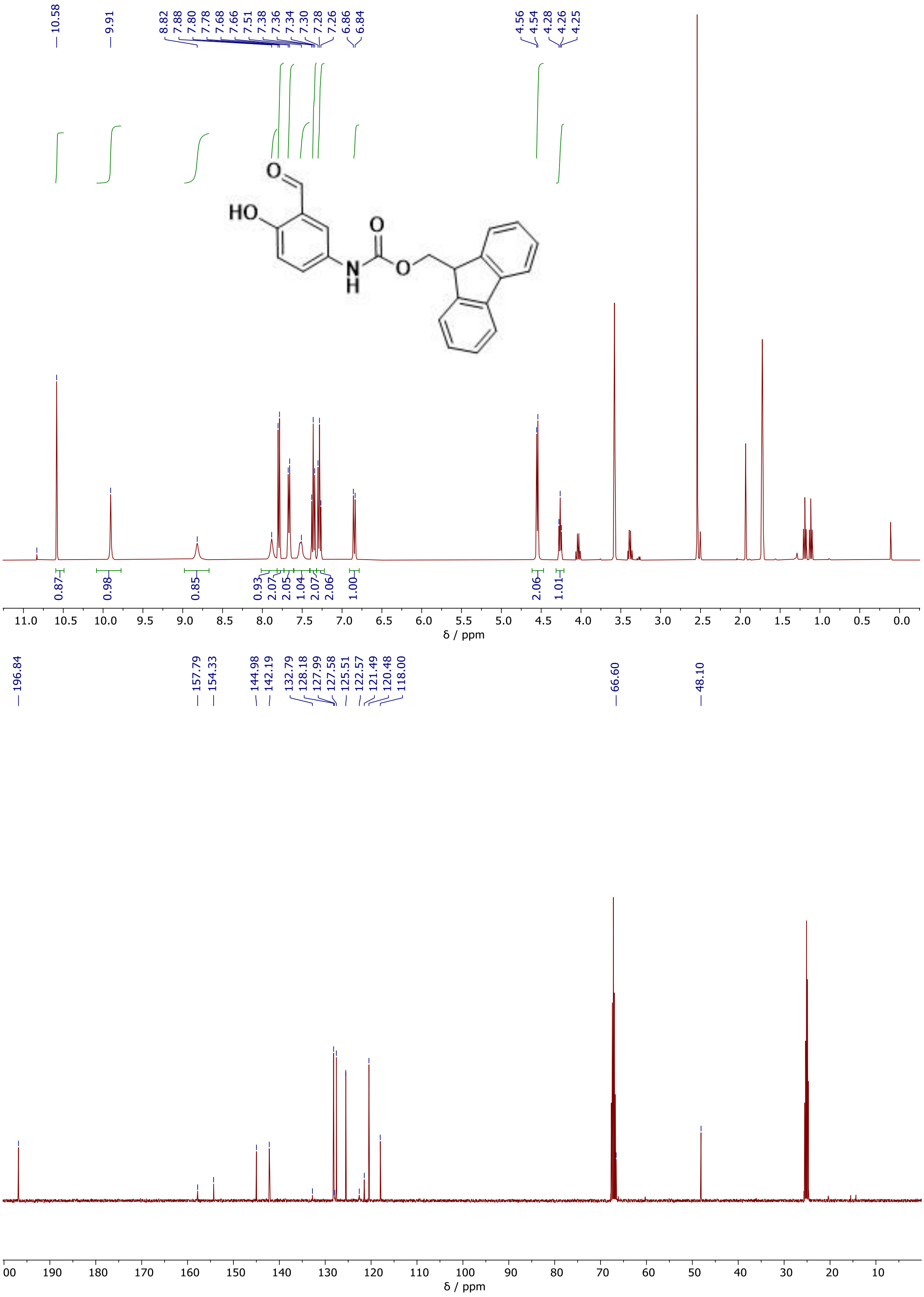

Figure S37. ^{1}H-NMR (top, 400 MHz) and ^{13}C-NMR (bottom, 100 MHz) in THF-d_8 of **10a**.

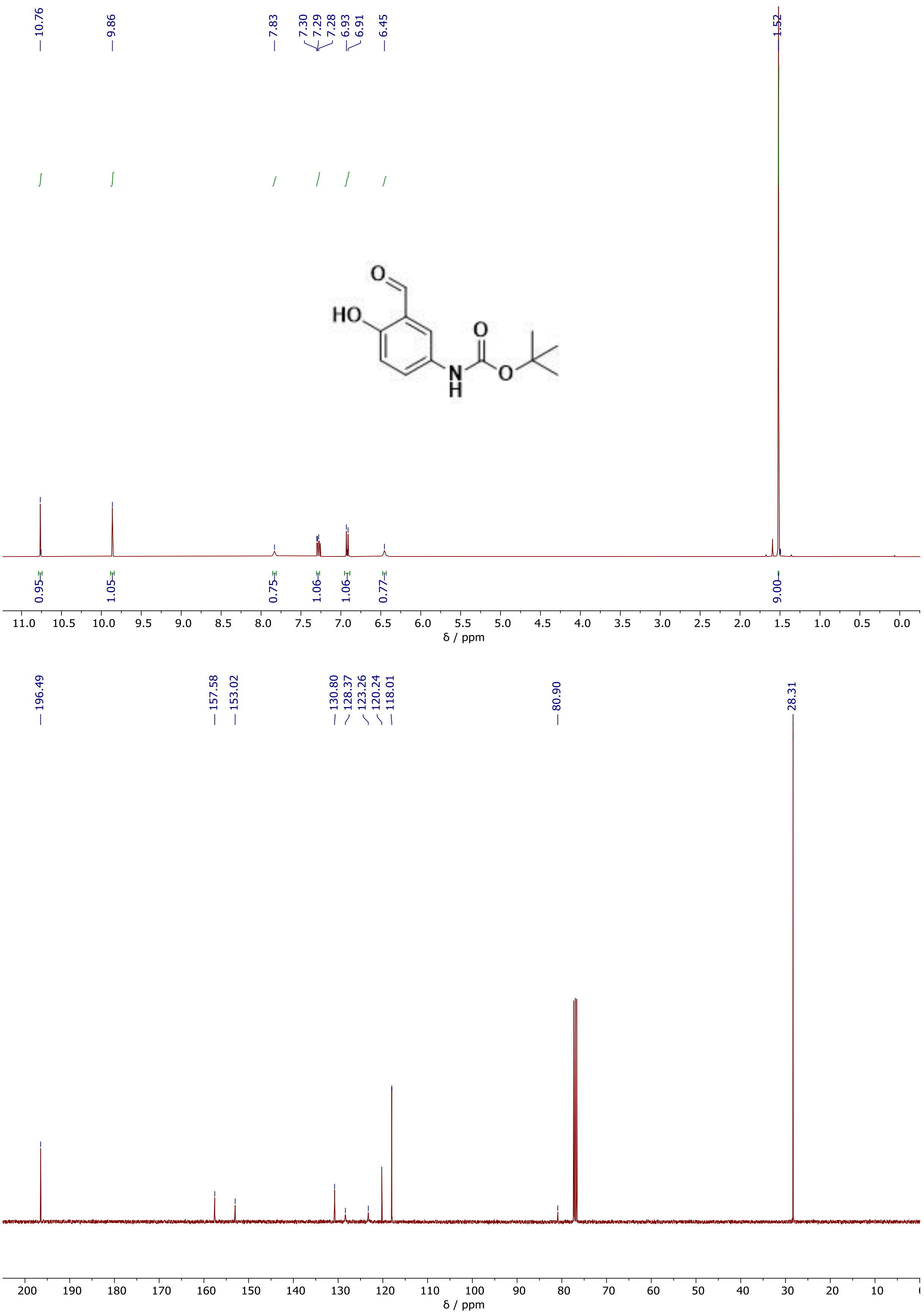

Figure S38. ^{1}H-NMR (top, 400 MHz) and ^{13}C-NMR (bottom, 100 MHz) in $CDCl_3$ of **10b**.

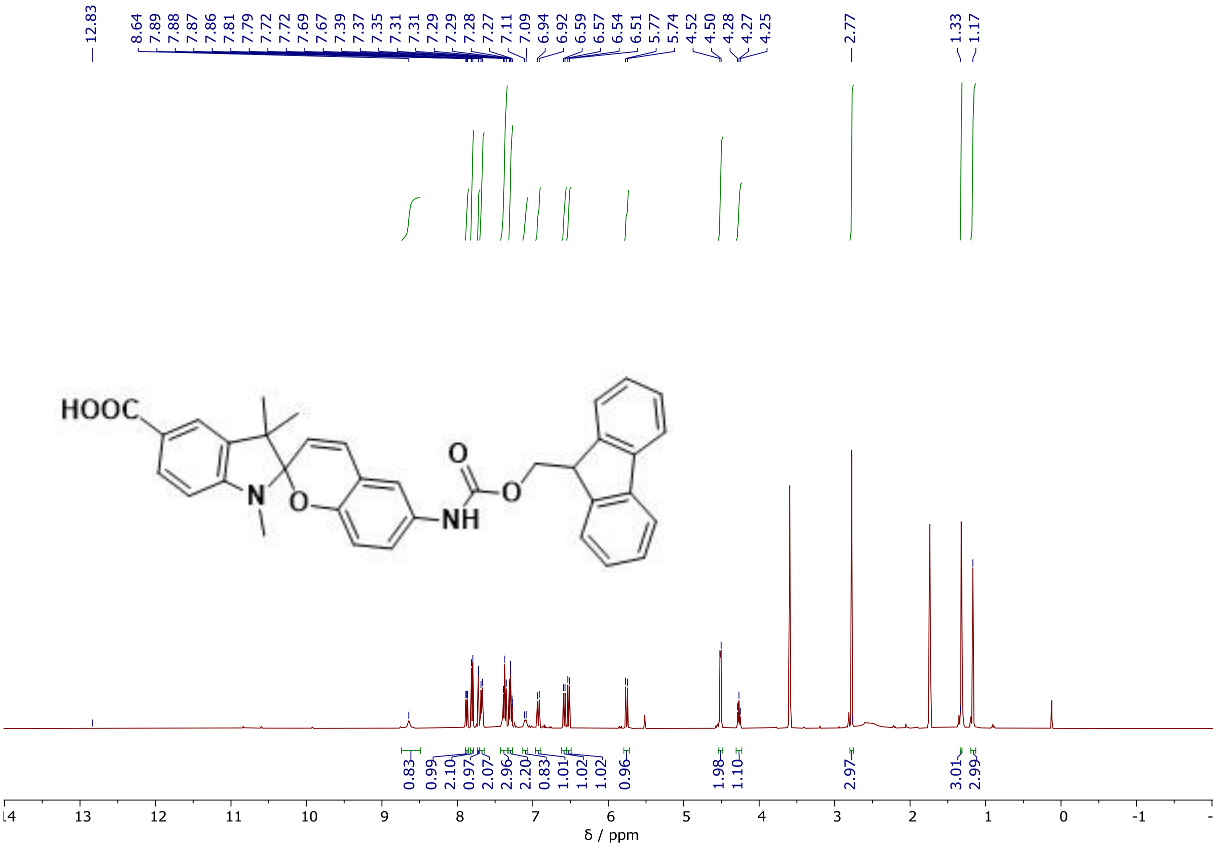

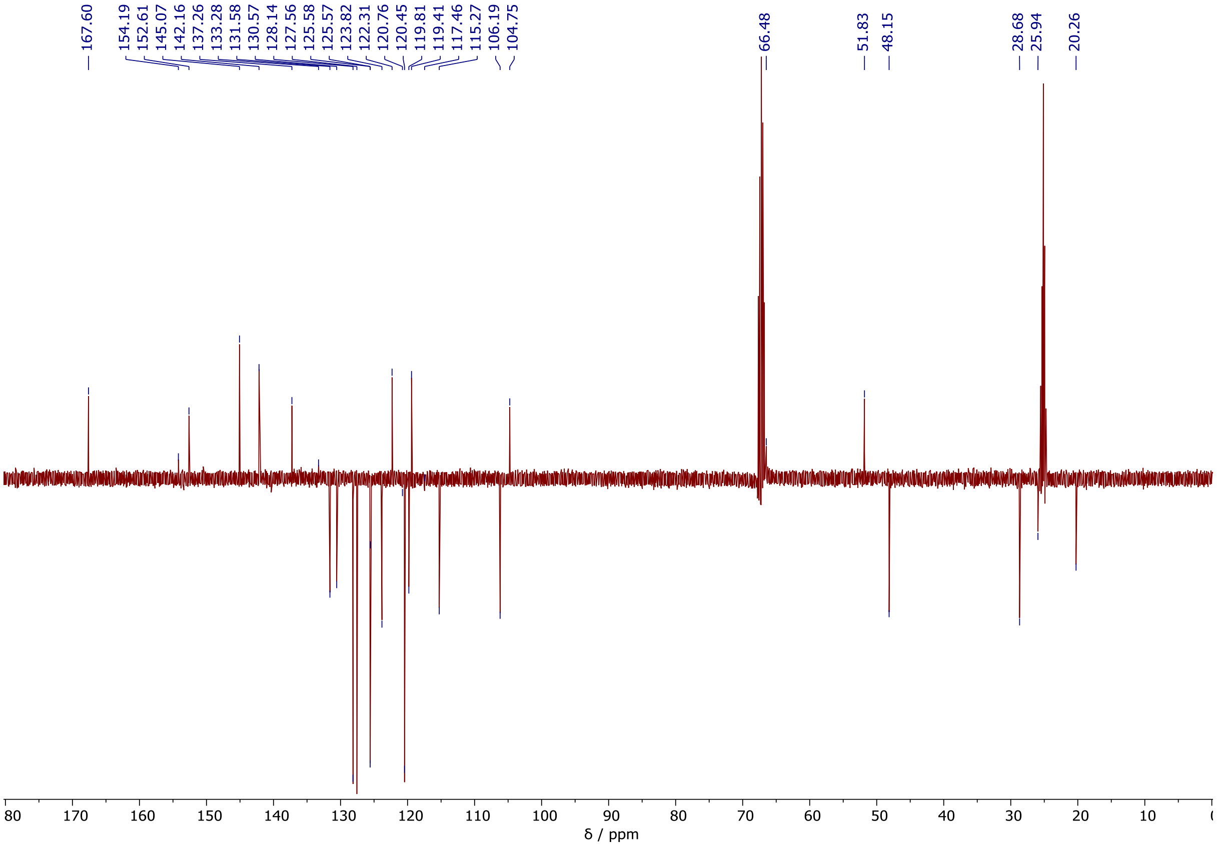

Figure S39. ^{1}H-NMR (top, 400 MHz) and ^{13}C-NMR (bottom, 100 MHz) in THF-d_8 of **2a**.

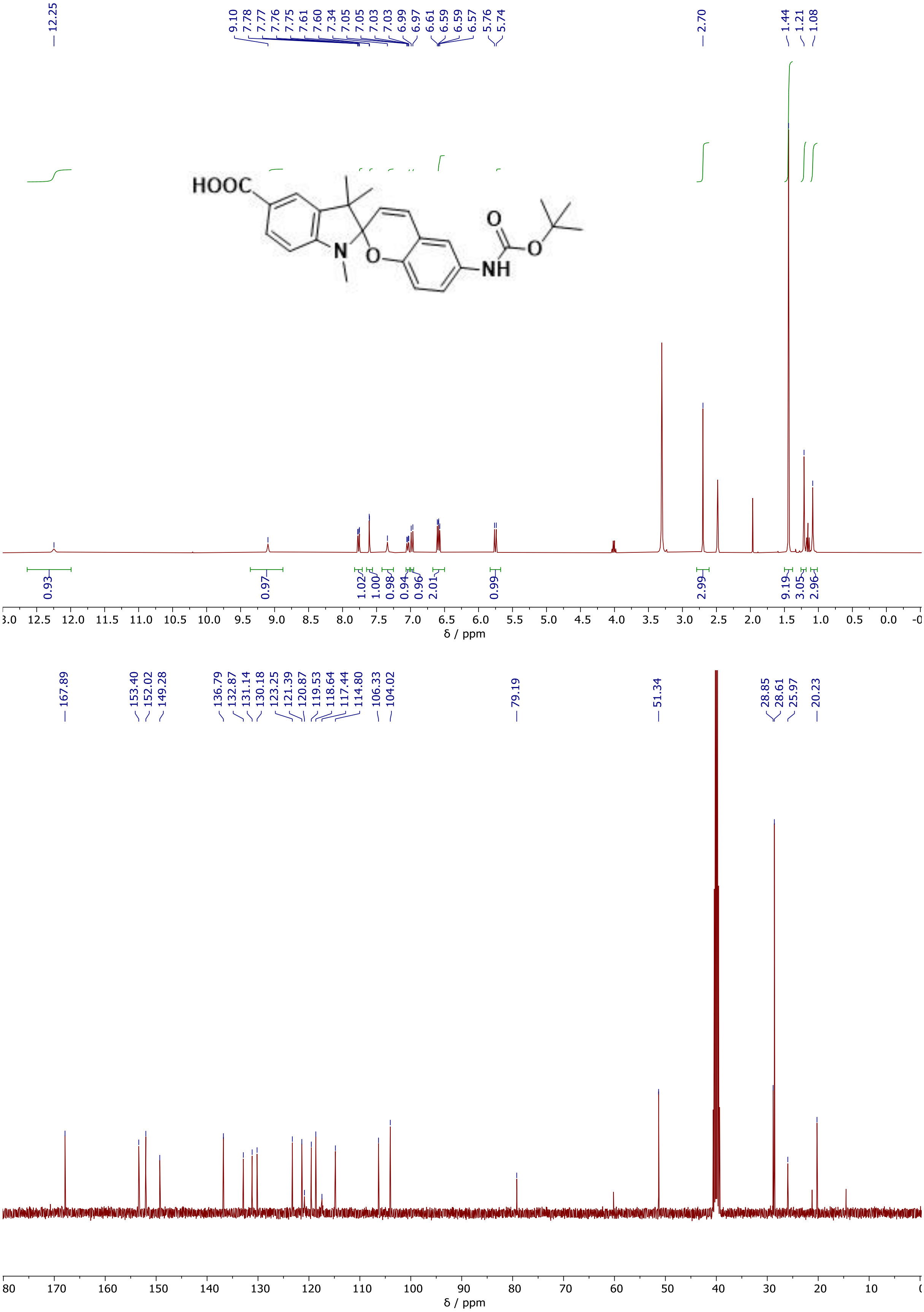

Figure S40. ^{1}H-NMR (top, 400 MHz) and ^{13}C-NMR (bottom, 100 MHz) in DMSO-d_6 of **2b**.

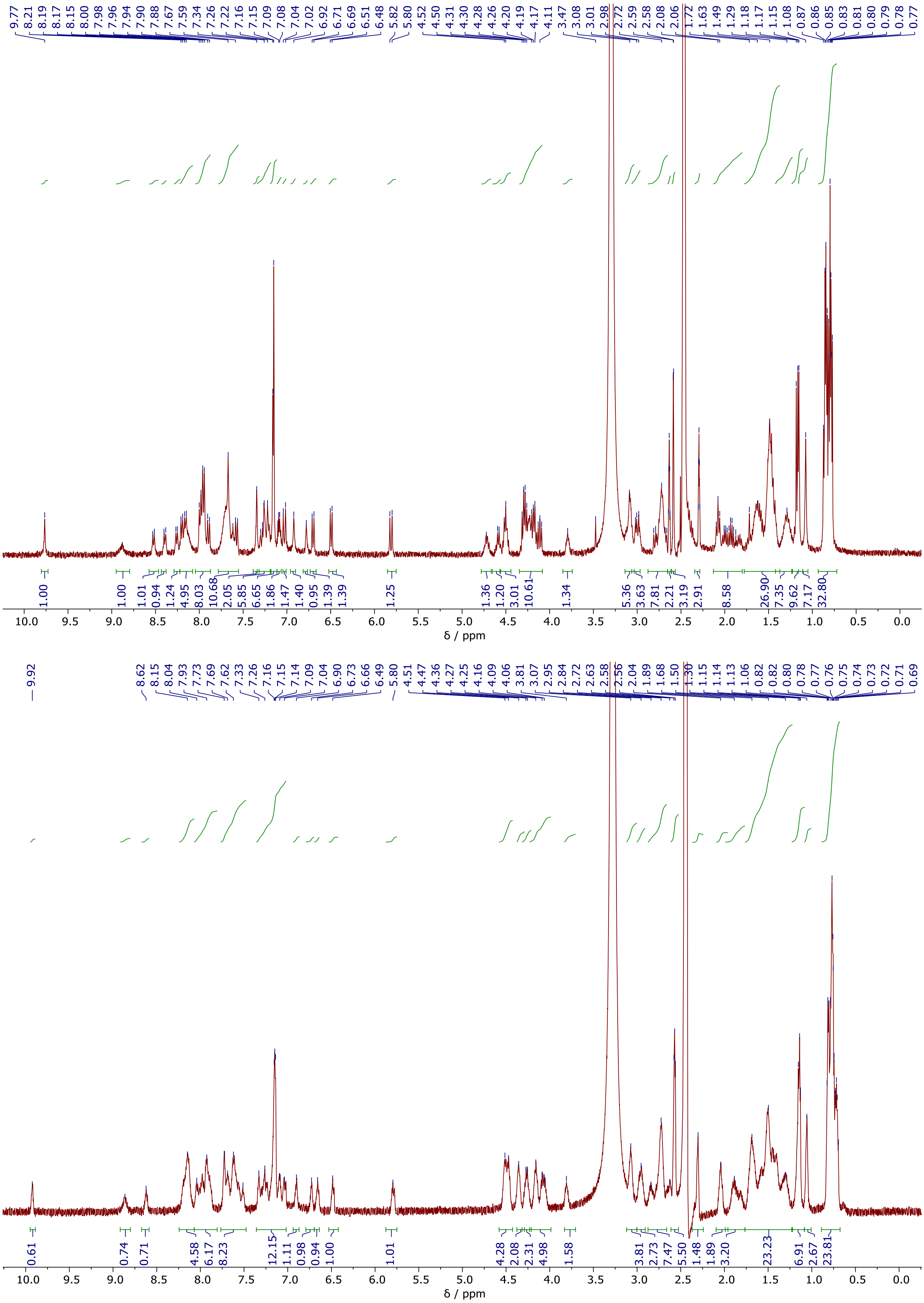

Figure S41: ^{1}H-NMR spectra (400 MHz, DMSO-d_6) of P1 (top, mixture of isomers) and P2 (bottom, mixture of isomers).

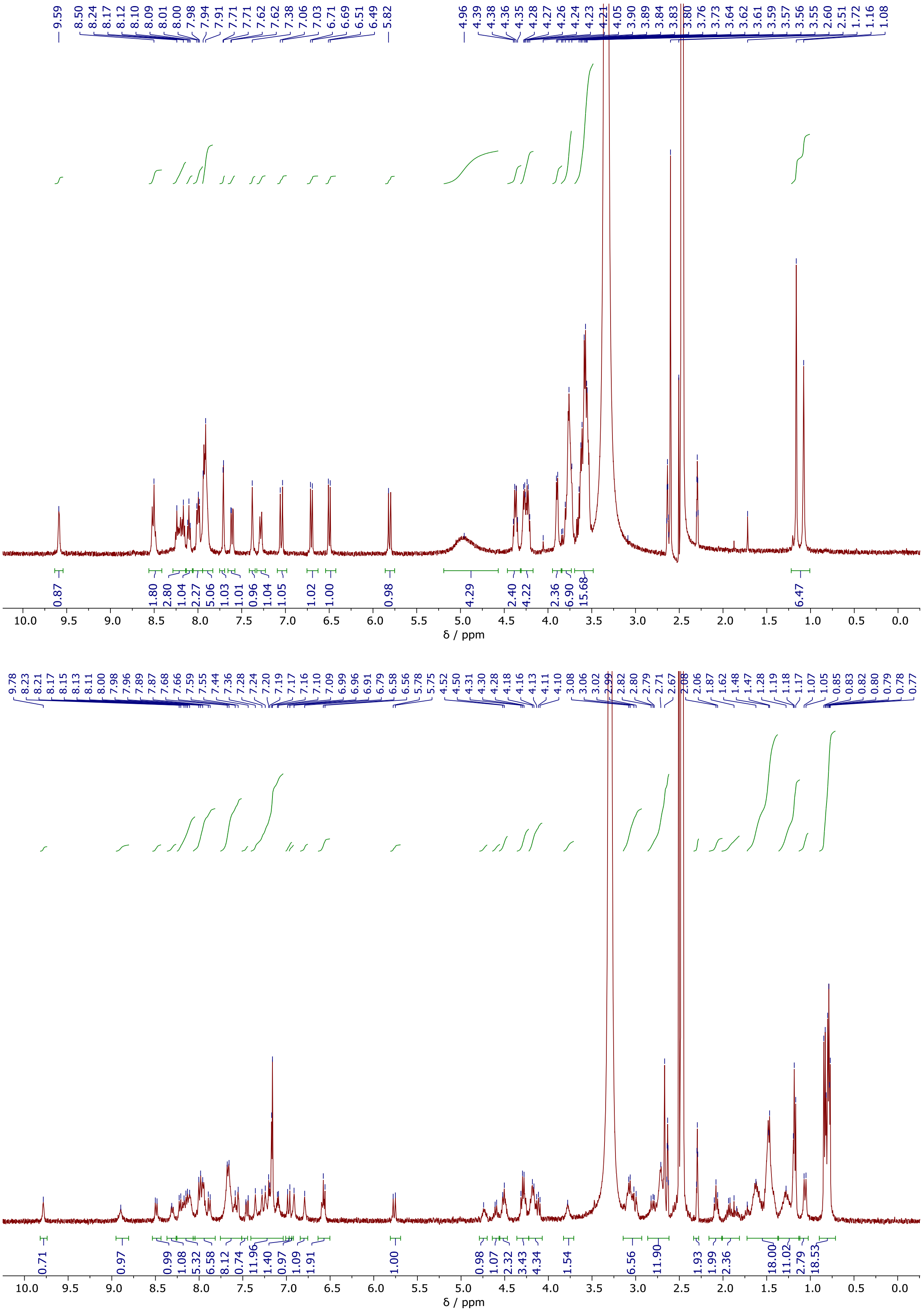

Figure S42: ^{1}H-NMR spectra (400 MHz, DMSO-d_6) of **P3** (top, mixture of isomers) and **P4** (bottom, mixture of isomers).

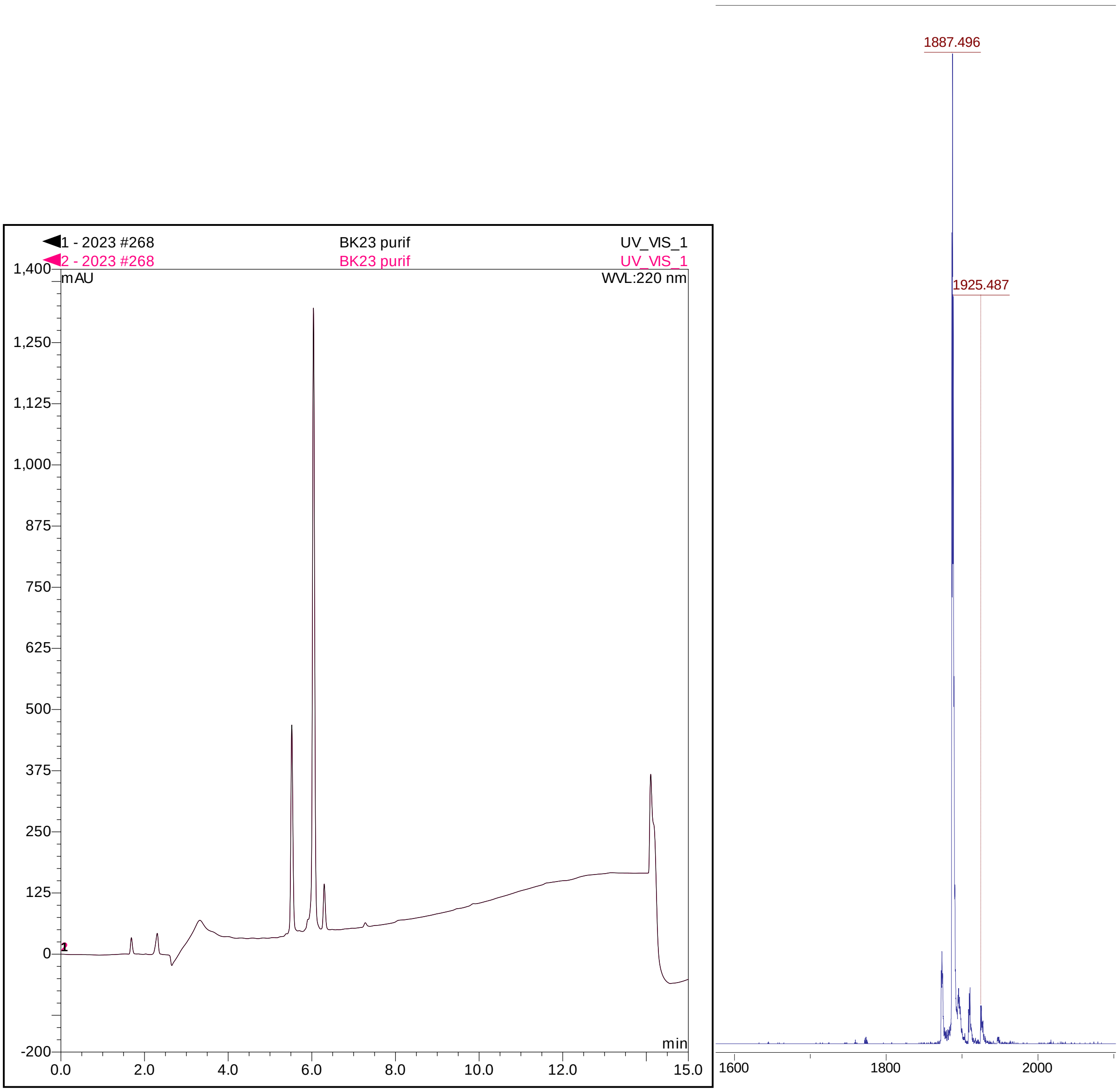

Figure S43: (**left**) HPLC-trace of P1 (MC- and SP-isomer between 5 and 7 min). (**right**) MALDI-spectrum of **P1** (*m/z* calculated: 1886.0435 found: 1887.496).

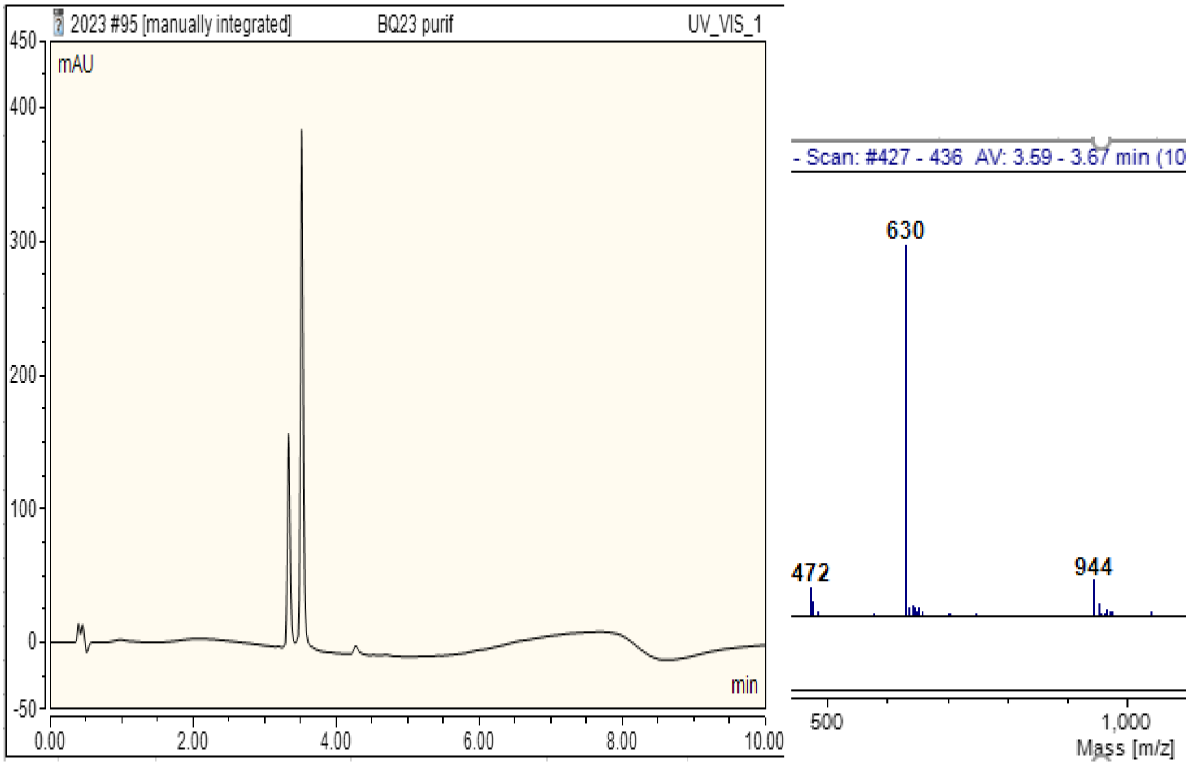

Figure S44: (**left**) HPLC-trace of P2 (MC- and SP-isomer between 3 and 4min). (**right**) LC-MS spectrum of **P2** (*m/z* calculated $[M+2H]^{2+}$: 945 found 945; calculated $[M+3H]^{3+}$: 630 found 630).

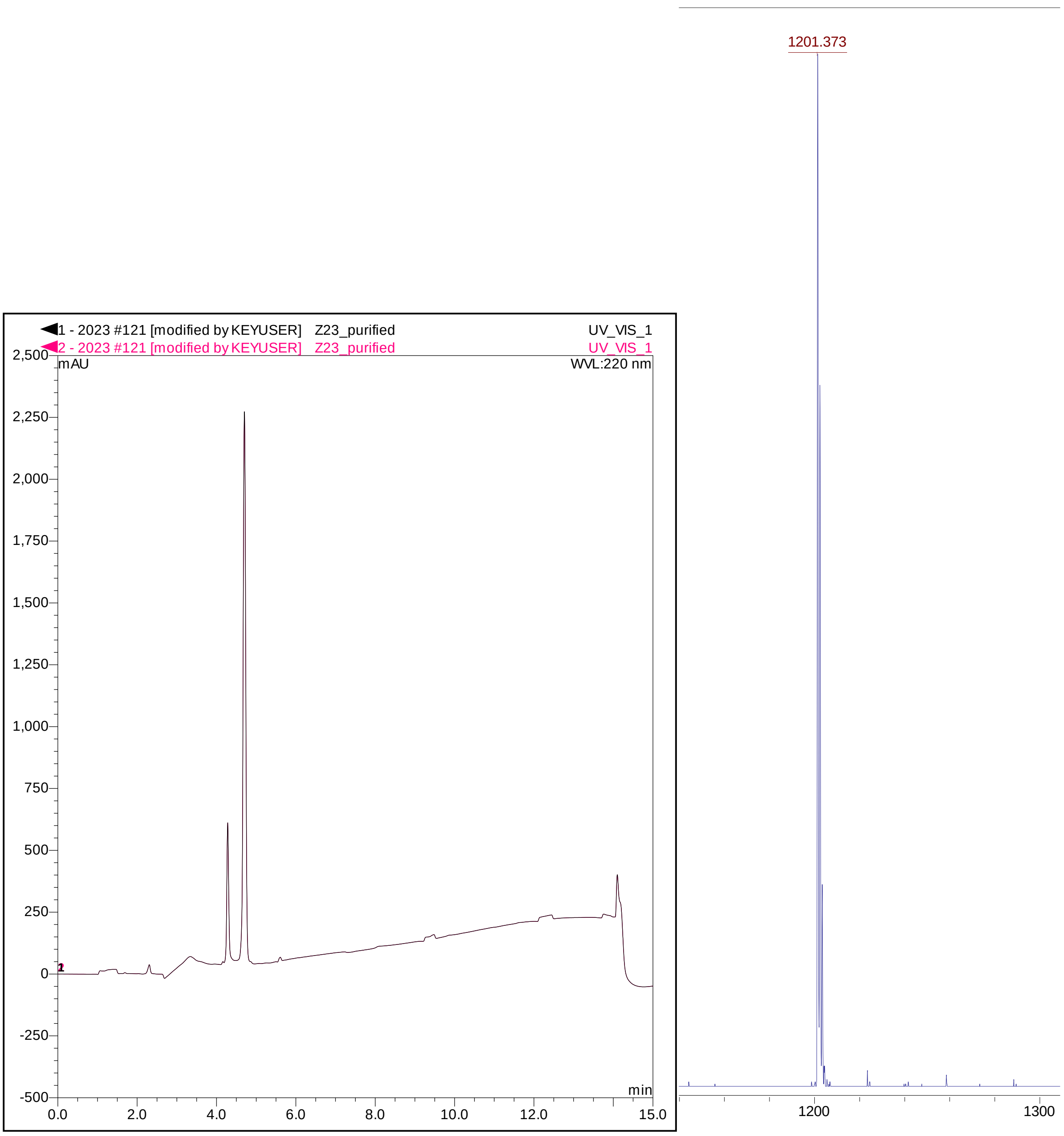

Figure S45: (**left**) HPLC-trace of P3 (MC- and SP-isomer between 4 and 5 min). (**right**) MALDI-spectrum of **P3** (*m/z* calculated: 1201.4756 found: 1201.373).

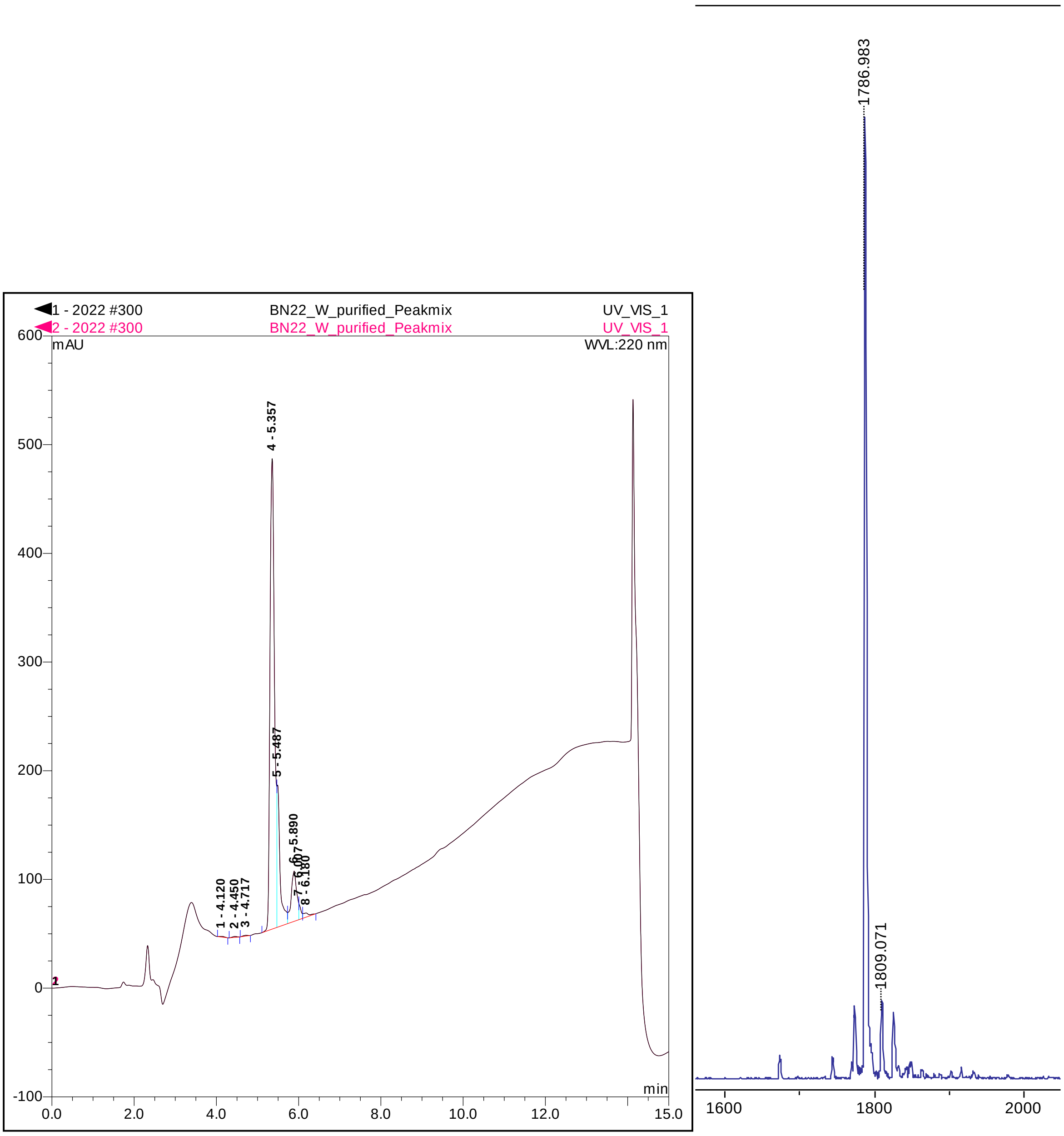

Figure S46: (**left**) HPLC-trace of P4 (MC- and SP-isomer between 6 and 7 min). (**right**) LC-MS spectrum of **P4** (*m/z* calculated $[M+H]^+$: 1787 found 1787).

(1) Sepehr, Z.; Nasr-Isfahani, H.; Mahdavian, A. R.; Amin, A. H. Synthesis, characterization, and UV–visible study of some new photochromic formyl-containing 1′, 3′, 3′-trimethylspiro [chromene-2, 2′-indoline] derivatives. *J. Iran. Chem. Soc.* **2021**, *18* (11), 3061-3067. DOI: 10.1007/s13738-021-02253-5.
(2) GALE, D. J.; WILSHIRE, J. F. Fibre–reactive Basic Dyes I–Polymethine Dyes Containing the N–Chloroacetyl Group. *J. Soc. Dye. Colour.* **1974**, *90* (3), 97-100. DOI: 10.1111/j.1478-4408.1974.tb03188.x.
(3) Tomasulo, M.; Kaanumal, S. L.; Sortino, S.; Raymo, F. M. Synthesis and properties of benzophenone− spiropyran and naphthalene− spiropyran conjugates. *J. Org. Chem.* **2007**, *72* (2), 595-605. DOI: 10.1021/jo062004d.
(4) Di Bella, S.; Consiglio, G.; Leonardi, N.; Failla, S.; Finocchiaro, P.; Fragalà, I. Film polymerization− a new route to the synthesis of insoluble polyimides containing functional nickel (II) schiff base units in the main chain. *Eur. J. Inorg. Chem.* **2004**, *2004* (13), 2701-2705. DOI: https://doi.org/10.1002/ejic.200300959.
